# Multitarget Approach to Drug Candidates against Alzheimer’s Disease Related to AChE, SERT, BACE1 and GSK3β Protein Targets

**DOI:** 10.3390/molecules25081846

**Published:** 2020-04-17

**Authors:** Larisa Ivanova, Mati Karelson, Dimitar A. Dobchev

**Affiliations:** Department of Chemistry, University of Tartu, Ravila 14a, 50411 Tartu, Estonia; larisa.ivanova@ut.ee (L.I.); mati.karelson@ut.ee (M.K.)

**Keywords:** Alzheimer’s disease, molecular docking, molecular dynamics, QSAR, neural networks, CADD, multifunctional drugs

## Abstract

Alzheimer’s disease is a neurodegenerative condition for which currently there are no drugs that can cure its devastating impact on human brain function. Although there are therapeutics that are being used in contemporary medicine for treatment against Alzheimer’s disease, new and more effective drugs are in great demand. In this work, we proposed three potential drug candidates which may act as multifunctional compounds simultaneously toward AChE, SERT, BACE1 and GSK3β protein targets. These candidates were discovered by using state-of-the-art methods as molecular calculations (molecular docking and molecular dynamics), artificial neural networks and multilinear regression models. These methods were used for virtual screening of the publicly available library containing more than twenty thousand compounds. The experimental testing enabled us to confirm a multitarget drug candidate active at low micromolar concentrations against two targets, e.g., AChE and BACE1.

## 1. Introduction

The leading cause of dementia worldwide is Alzheimer’s disease (AD). It is a devastating neurodegenerative condition responsible for the loss of memory and other cognitive abilities such as poor short-term memory, difficulty speaking, disorientation, mood and behavioral issues, and an ultimately fatal decline in bodily functions. Such detrimental condition is associated to several risk factors that may lead to considerable implications along with the colossal financial and emotional burden on the patients and their families. One of the terrible and devastating aspects of the AD is the loss of cognitive abilities that currently accounts for 50 million cases worldwide, predominantly affecting senior citizens. The forecasts indicate that this number will be more than triple to 152 million by 2050 [1]. The disease progresses symptomatically from mild to severe and found its place amongst the eight topmost health complications worldwide [2].

The key for developing new therapies against AD is hidden in the deeper understanding of the molecular mechanisms responsible for the disease [3]. The undergoing research on the discovery of novel therapeutics is targeting different molecular mechanisms and phenomena [1] including to amyloid deposition [4], astrogliosis [5], tau protein hyperphosphorylation and accumulation [6], neuronal dystrophy [7], oxidative stress [8], biometal dyshomeostasis, decline in acetylcholine (ACh) levels [9], etc. The rigorous research findings have revealed that amyloid beta (Aβ) and tau protein are the key constituents of the plaques and neurofibrillary tangles (NFTs) involved in the molecular pathogenesis of the disease. Tau protein is the major constituent of neurofibrillary tangles in Alzheimer’s disease [1]. It can directly interact with nucleoporins of the nuclear pore complex (NPC) and affect their structural and functional integrity. Pathological tau impairs nuclear import and export in tau-overexpressing transgenic mice and in human AD brain tissue. Furthermore, the nucleoporin Nup98 accumulates in the cell bodies of some tangle-bearing neurons and can facilitate tau aggregation in vitro. The work also showed that the NPC dysfunction contributes to tau-induced neurotoxicity in AD and tauopathies [10]. However, alternative mechanisms have been proposed as causes for AD. A study based on the analysis of various types of data from postmortem tests of brain tissue proposed that herpes viruses HHV-6A and HHV-7 could be involved in the development of the disease [11]. In another recent work, the main pathogen in chronic periodontitis, *P. gingivalis*, was identified in the brain of Alzheimer’s disease patients. Toxic proteases from the bacterium, called gingipains, were also identified in the brain of Alzheimer’s patients, and the levels of specific toxic proteases from this bacterium correlated with tau and ubiquitin pathology [12]. Nevertheless, the exact cause of AD is still unclear, and no curative therapy has been discovered to date. Current treatment strategies encompass the use of FDA approved medications like acetylcholinesterase (AChE) inhibitors and NMDA-receptor antagonists 3 [13]. Despite the amounted research carried out, no drug therapy has been proven effective in halting disease progression in those with more advanced Alzheimer’s and, therefore, the search for new pharmaceutical agents against this disorder is of high importance.

The standard drug discovery research is usually credited to a single target and/or related homologues and thus the scientific outcome is limited to a certain target. One promising approach in the development of novel therapeutic candidates against Alzheimer’s disease is the multitarget-directed approach [14]. This approach is focusing on chemical compounds that are active simultaneously to several biological targets related to the disease. Presumably, such candidates would be more effective as acting simultaneously against several adverse factors of AD.

Sharma et al. [15] developed several *N*-benzylpiperidine analogs which exhibited at the same time from moderate to excellent inhibition (IC_50_ from 0.90 µM to 0.22 µM) of AChE and BACE1 proteins. The discovery of these compounds proceeded from virtual screening of compound libraries using molecular mechanics/dynamics. The best structures were designed and synthesized as multifunctional inhibitors that were further tested for brain permeability (PAMPA-BBB tests). Two of the candidates showed high permeability. Moreover, improvement in Aβ_1-42_-induced cognitive impairment (rats model) was also observed experimentally, resulting in significant oral absorption characteristics ascertained by the pharmacokinetic studies.

Cong et al. have developed a promising series of hydroxylated chalcones that were designed and synthesized as dual-functional inhibitors to inhibit amyloid-β peptide (Aβ) aggregation as well as ferroptosis simultaneously [16]. The authors used computational methods to obtain the assessed ADMET properties for the selected candidates. Other researchers concentrated on the development of tetrahydroisoquinoline-benzimidazoles that act as multifunctional agents against AD [17]. The chemical structures were systematically constructed by assembling the fragments benzimidazole and tetrahydroisoquinoline. Among the synthesized compounds, some compounds exhibited great inhibition of neuroinflammation and BACE1, as well as a good neuro-protective effect and blood-brain barrier penetration. In silico molecular docking computations indicated that the best compound can inhibit BACE1 by interacting with the catalytic pocket in BACE1. The results were also confirmed by in vitro experiments.

Gonzalez-Naranjo et al. [18] showed that cannabinoids such as indazolylketones are promising multitarget agents for the development of new drug therapies against AD. The author’s group has developed a new family of indazolylketones with a multitarget profile embracing cholinesterase and BACE1 activity. They used molecular docking and dynamics to investigate the favorable characteristics of the ligands within the protein’s pockets. The best candidates were synthesized and experimentally tested. The final set included nine indazolylketones with significant multifunctional activity for the above targets.

Wang et al. discovered several novel isoflavone analogs as multitarget-directed ligands for Histamine 3 receptor (H3R) and acetylcholinesterase [19]. They have performed in vitro experiments which indicated significant inhibition toward AChE (the best candidate with IC_50_ = 0.081 µM). The authors also carried out a molecular modeling study to further elucidate the binding interactions between the best compound and AChE or H3R. Thus, they were able to indicate important region characteristics of the binding interface that would lead to higher activities of potential drug candidates.

In the current study, we tackled a challenging task to develop potential multifunctional drug candidates against AD that would be active against four protein targets, namely AChE, BACE1, GSK3β and serotonin transporter (SERT).

Acetylcholinesterase has proven to be the most viable therapeutic target for symptomatic improvement in Alzheimer’s disease because cholinergic deficit is a consistent and early finding in AD. Currently, available AChE inhibitors for AD treatment include galantamine, rivastigmine, and donepezil [20]. The glycogen synthase kinase-3 (GSK-3) is regarded as a critical molecular link between the two histopathological hallmarks of the Alzheimer’s disease, namely senile plaques and neurofibrillary tangles [21]. As regard to BACE1, according to the “Amyloid Cascade Hypothesis” the critical molecular event in the pathogenesis of AD is the accumulation of Aβ neurotoxic oligomers. Since the proteolytic processing of Amyloid Precursor Protein (APP) by β-secretase (beta-site APP cleaving enzyme 1, BACE1) is the rate-limiting step in the production of Aβ, this enzyme is considered also as a major therapeutic target and BACE1 inhibitors have the potential to be disease-modifying drugs for AD treatment [22]. Finally, most of the publications dealing with the role of serotonin receptors in AD focus on the possible interplay between the serotonergic system and the amyloid-mediated part of pathophysiology, i.e., the interplay is strongly related to the sodium dependent serotonin transporter (5-HT transporter) [23].

In the present work we used state-of-the-art computational techniques as combined molecular modeling (molecular docking and molecular dynamics), multilinear regression statistical analysis, artificial neural networks (ANN) methods as well as virtual screening procedures. Such robust in silico methods have been proven to be effective for computer-aided-drug-design (CADD) for many CNS areas including AD [24].

## 2. Results and Discussion

### 2.1. Computational Studies

**Figure 1:**
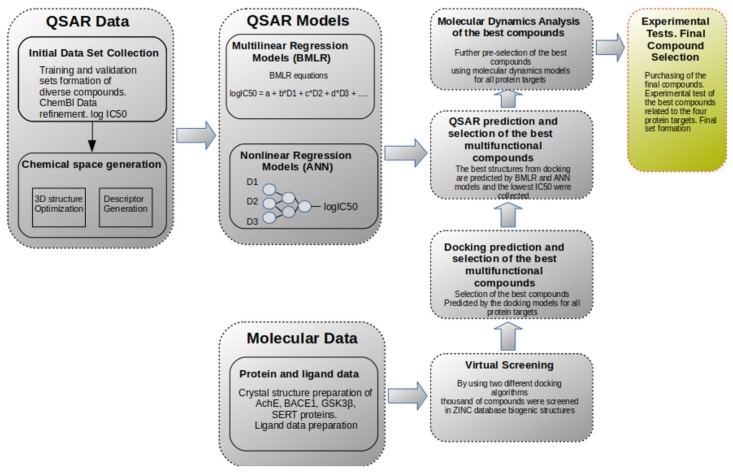
General workflow of the computational studies.

The workflow of the computational studies is summarized in Figure 1. It includes several stages related to the development of quantitative structure–activity relationships (QSAR) and molecular modeling, respectively. All stages start with the preparation and curation of the initial molecular data. By comparing the predictions from both types of computations, it is possible to generate compounds which would be active against all four protein targets (or at least simultaneously to some subset of them). The molecular dynamics were applied in the final set of the best candidates in order to elucidate important molecular characteristics related to the ligand-protein interface. Such molecular characteristics would provide us with valuable information for future development and selection of improved compounds. Lastly, the main logical goal of the two approaches is to converge for the selection of the best compound candidates before the experimental tests are carried out.

In short, the general workflow proceeded as follows (see Figure 1): first, the training data set was prepared for the development of QSAR models in Section 3.1.1 (also Section 3.5). The protein data preparation is described in Section 3.1.2 and Section 3.2. The second stage included the development of the QSAR models as described in Section 3.6 and Section 3.7. For virtual screening, 20,397 small compounds (MW < 600) were extracted from the ZINC database (subsection biogenic). The virtual screening involved high-throughput molecular docking followed by QSAR predictions. Next, molecular dynamics simulations were carried out for the best candidates for each of the proteins. Finally, the best candidates were experimentally tested for simultaneous activity against all targets and the best multifunctional compounds were selected.

### 2.2. BMLR Models

The main purpose of the BMLR models developed herein is their fast utilization in the predictions and easy interpretation of the models based on their molecular descriptors. The models are used together with the ANN models in Section 2.3 to predict and confirm the best multifunctional candidates obtained by the docking results in Section 2.5.

**Figure 2:**
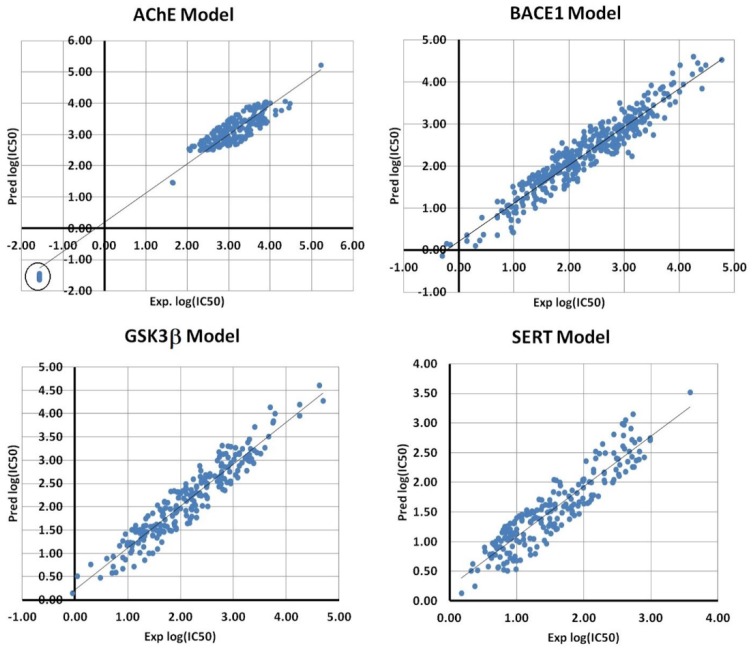
Scatter plots between experimental and predicted log (IC_50_) values for all BMLR models.

Four BMLR models were developed that are related to each protein set. In Table 1, the best equations and their statistical parameters are shown. As can be seen from Table 1, all models possessed significant quality as reflected in their coefficient of determination R^2^. The visual presentation of the linear plots between the measured and predicted log (IC_50_) values are shown in Figure 2 for all four sets.

The model with highest R^2^ adjacent to the SERT dataset has in R^2^ = 0.85 and includes only four independent variables describing a data set of N = 213 datapoints. The predictive stability of this model is reflected by the high correlation coefficients of the cross-validation statistics. Furthermore, the low correlation coefficients of the randomization tests indicate no chance descriptors were included.

It can be seen from Table 1 that the model with the highest overall R^2^ is related to the AChE dataset (R^2^ = 0.93). However, as can be seen from Figure 2, the correlation plot for this model indicates that 10 compounds (circled island) are apart from the main grouping. These compounds included specific fragment (sulfamoylcarbamic acid-like) that was available only for them and had very close IC_50_ values. Additionally, these compounds possessed very good measured inhibitory activity for AChE protein. Moreover, the descriptors of the AChE BMLR model are largely related to the atomic content of their specific fragment. Therefore, for the sake of generality and the reasons mentioned above, we did not exclude them from the data set.

The model for BACE1 had the largest training data N = 378 (see Table 1). The best multilinear regression equation developed had significant quality, with R^2^ = 0.91 fitting the data with just four molecular descriptors. It also had an excellent predictive quality indicated by R^2^_cv_ = 0.91 and R^2^_abc_ = 0.90. Similar statistical characteristics were observed also for the BMLR model of GSK3β data (R^2^ = 0.90, R^2^_cv_ = 0.90, R^2^_abc_ = 0.90). However, this model was built on a lesser number of data N = 229.

It should be mentioned that common characteristics for all models are high statistical quality, a large number of training datapoints and a low number of independent variables. It is an evidence for the power of the BMLR algorithm (see Section 3.4) to select significant descriptors chosen from a large space of variables. Therefore, all descriptors in Table 1 were included in the refined descriptor subspace for the ANN modeling.

By analyzing the Student’s t-statistics of the models in Table 1, the most statistically significant descriptor in the BMLR equation can be identified. In the case of the AChE model, the most significant descriptor is D_1_—the lowest resonance energy (AM1) for N–S bonds. This descriptor is derived by semi-empirical quantum mechanics calculations related to the N–S bonds. Its importance is related to the N–S containing fragments in the structures. It leads to a positive correlation with log (IC_50_). The stability of the compounds is related to D_3_—the final heat of formation per atom which the second statistically significant descriptor in the model for AChE. Descriptor D_4_ is the Highest e–e repulsion (AM1) for N–H bonds, suggesting that the amine group in the molecule is important and it has a negative correlation with the dependent variable. The last descriptor is D_2_—the maximum net atomic charge (AM1) for O atoms in this model indicates the importance of the oxygen-containing compounds and their charge as calculated by the AM1 scheme.

The most statistically significant descriptor in the QSAR model for BACE1 data set is D_2_—the Structural Information content (order 2). This descriptor accounts for the diversity of the atomic constitution and it is also related to molecular complexity from the viewpoint of the information theory. It has a negative correlation with log (IC_50_). This is similar to the descriptor D_4_—Balaban Index is attributed to the molecular shape. The remaining two descriptors are D_1_—the PPSA2 Total charge weighted PPSA (AM1) and D_3_—Average valency (AM1). The former accounts positive charged surface area and the latter is related to the valency of the atoms of the molecule. Apparently, most of the descriptors are related to the shape and the atomic constitution of the structures.

The molecular descriptors involved in the best QSAR model for the GSK3β data set are mostly related to the hydrogen bonding ability of the molecule (D_1_—HA dependent HDCA-2 (AM1) and D_3_—HACA-2 (Zefirov)) and reactivity of the compounds (D_2_—LUMO energy (AM1)). According the t-statistics of the independent variables, the statistical significance (|t|) order is as follows: D_1_ > D_2_ > D_3_ > D_4_. The descriptor D_1_ has a negative correlation with log (IC_50_) while the remaining descriptors have a positive correlation.

The descriptors for the best QSAR model for the SERT dataset resemble the descriptors for the BACE1 model. Again, here the nature of the most statistically significant descriptors is topological and related to the shape and atomic connectivity of the compound (D_1_—the Kier shape index (order 3) and D_3_—the Bonding Information content (order 1)). Their significance decreases as follows: D_1_ > D_3_ > D_4_ > D_2_. The last two descriptors are D_4_—the FHASA Fractional HASA (HASA/TMSA) and D_2_—the Lowest n–n repulsion (AM1). The former is addressed to the fractional hydrogen acceptor surface area and the latter is a quantum chemical in nature and related to the nuclear–nuclear repulsion of the atom as calculated by the AM1 method. Thus, these molecular features are important for hydrogen bonding interactions (D_4_) as well as rotational and conformational changes (D_2_) of the molecule.

### 2.3. ANN Models

The nonlinear QSAR models developed herein are based on backpropagation ANN as described in Section 3.5. Similarly, as it was in the case of the linear models in Section 2.2, we have built four network models regarding the protein sets for AChE, BACE1, GSK3β and SERT. In this exercise, it was stressed on building models that have high validation set R^2^_val_ values (low RMS_val_), rather than high R^2^_tr_ statistics (low RMS_tr_). The network prediction values of log(IC_50_) were averaged with the log(IC_50_) values calculated by the linear models of Section 2.2. Consequently, these averaged values were used for the final identification of the active compounds.

**Figure 3:**
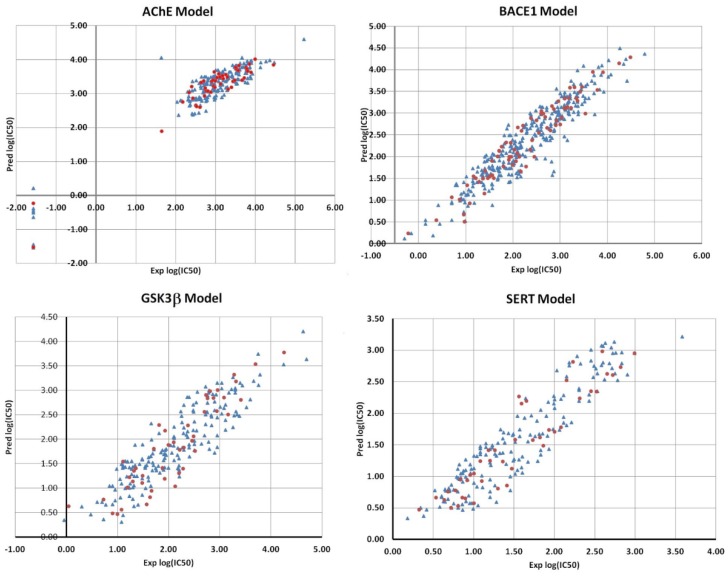
Scatter plots between predicted and experimental log (IC_50_) values for all ANN models. Legend: training datapoints—blue triangles; validation datapoints—red dots.

The first ANN model developed was for the AChE dataset. The initial set was divided into subsets of 192 and 47 datapoints for the training and validation data, respectively. The correlation plot between the ANN predicted and experimental log(IC_50_) values is presented in Figure 3. The correlation coefficients of the linear relationship between the predicted and experimental log (IC_50_) values reflect the good predictive ability of the model: R^2^_tr_ = 0.878 (RMS_tr_ = 0.126) and R^2^_val_ = 0.911 (RMS_val_ = 0.167).

The best model architecture was 4-4-1, i.e., four input descriptors, one hidden layer with four neurons and one neuron in the output layer related to the log (IC_50_). It should be mentioned that all ANN models (for all targets) have only one neuron in the output layer for log (IC_50_). The input descriptors for this net were Maximum net atomic charge (AM1) for O atoms, Final heat of formation (AM1)/# atoms, Highest e–e repulsion (AM1) for N–H bonds, and Average atom weight. The weight analysis of this model indicated that the descriptor Highest e–e repulsion (AM1) for N–H bonds is the most influential in the predictions. As can be noted from Table 1, all descriptors are the same as for the BMLR model with the exception of Average atom weight. Therefore, the same analysis of the physical meaning of the descriptors is still valid here. Regarding the deviating compounds (see Figure 3), the model is capable of predicting their values with good accuracy, as indicated by the validation points (red dots) in this region. It should be mentioned that the validation set was not included explicitly in the training of the weights. It was used as an external data set to control the stopping RMS_val_ values.

The next ANN model concerns the BACE1 data which were separated into 303 datapoints for the training set and 75 for the validation set. It should be noted that the model has excellent statistical parameters R^2^_tr_ = 0.874 (RMS_tr_ = 0.123) and R^2^_val_ = 0.913 (RMS_val_ = 0.341) bearing in mind the large number of datapoints. Moreover, the topology of the net resulted in only three input descriptors, i.e., 3-4-1. These descriptors are Complementary Information content (order 2), Total molecular 2-center exchange energy (AM1) and Balaban index. The Balaban index is the same descriptor as in the BMLR model. The weight values indicated that the Complementary Information content (order 2) is the most influential descriptor regarding future predictions. Figure 3 shows that the predictions for the validation data points are well within the main region without any significant outliers.

The best ANN algorithm resulted in two ANN models with three and four input descriptors, respectively, for the GSK3β set. However, we have chosen the model with three descriptors, thus 3-3-1 topology was further used. The final trained model 3-3-1 resulted in good predictions for both training (184 datapoints) and validation (45 datapoints) sets with R^2^_tr_ = 0.80 (RMS_tr_ = 0.181) and R^2^_val_ = 0.814 (RMS_val_ = 0.254). The correlation plot for the predictions is shown in Figure 3. The three descriptors for the model are HA dependent HDCA-1 (AM1) (all), LUMO energy (AM1), HACA-1 (Zefirov). The LUMO energy is the same descriptor as in the BMLR model for GSK3β, while the remaining two are similar to the BMLR descriptors. Therefore, it seems that the hydrogen-bonding ability of the compounds is an important factor for IC_50_. The weight magnitude in the hidden layer suggested that the HACA-1 (Zefirov) is the most influential.

The last model, derived by using the BeANN algorithm, was for the SERT dataset. The model obtained had 4-4-1 architecture. Its statistical parameters resulted in R^2^_tr_ = 0.86 (RMS_tr_ = 0.173) for the training set of 171 datapoints and R^2^_val_ = 0.86 (RMS_val_ = 0.233) for the validation set of 42 datapoints. The scatter plot between the predicted and experimental log(IC_50_) values are presented in Figure 3 (lower right corner). The four input descriptors that appear in the model are Kier shape index (order 3), Lowest n–n repulsion (AM1), Bonding Information content (order 1), FHASA Fractional HASA (HASA/TMSA) (AM1). Notably, these descriptors coincide with the descriptors for the respective BMLR model. Again, the Bonding Information content (order 1) is the most important variable according to the weight analysis.

In summary, all models developed herein possessed good statistical quality and predictability for the data. In these calculations, the large training sets allowed us also to develop models with greater number of input descriptors than the selected ones. However, we tried to keep the number of free parameters as low as possible in order not to overparameterize the nets. The ANN models are further used to predict the log (IC_50_) values of the docking results obtained in Section 2.5.

### 2.4. Virtual Screening with Glide VSW Module of the Schrödinger Suite

A virtual screening using the Glide VSW module of Schrödinger [25] was carried out for the compounds obtained from ZINC database. In result, around 500 compounds with the highest docking-free energies and/or ligand efficiencies were selected for each target protein. For these compounds, the binding energies and ligand efficiencies (LE) were as follows:

- AChE: 526 compounds; ∆G = −18.71…−10.97 kcal/mol; LE = 0.32…0.71
- BACE1: 457 compounds; ∆G = −12.69…−6.72 kcal/mol; LE = 0.19…0.48;
- GSK3β: 506 compounds; ∆G = −20.39…−12.27 kcal/mol; LE = 0.31…0.79;
- SERT: 526 compounds; ∆G = −15.47…−8.99 kcal/mol; LE = 0.24…0.53.

At first, the results of virtual screening for each target were compared among themselves to search for compounds included in the top/best compounds for each target. The main condition for the identification of such compounds was a ligand efficiency no less than 0.3. Unfortunately, it was not possible to find compounds having simultaneously good binding energy and ligand efficiency for four. Only two compounds were detected that that bind well to two targets (Table S1). The subsequent treatment of the top 60 compounds for each target using Glide docking module with an extra precision level did not reveal additional compounds with good binding toward several targets simultaneously and at the same time to satisfy Lipinski’s rule of three [26].

### 2.5. Virtual Screening with Autodock Vina 1.1.2

Since the Glide virtual screening within VSW module of Schrödinger and Glide molecular docking with extra precision level resulted only in two compounds with good binding toward two target proteins, the virtual screening of the ZINC library of compounds was also carried out using AutoDock Vina 1.1.2 [27]. The results of the screening for each target were filtered according to the following three conditions: (1) all compounds with a binding energy greater than −8.0 kcal/mol or with a ligand efficiency less than 0.35 were excluded from further analysis; (2) compounds satisfying at least three of the four conditions for Lipinski’s rule were selected; (3) compounds with a low solubility category were excluded from further analysis. The solubility category was calculated using JChem software [28] as a qualitative measure (low: if solubility is <0.01 mg/mL; moderate: if solubility is between 0.01 and 0.06 mg/mL, high: if solubility is >0.06 mg/mL). Finally, the following results were obtained for each target:

- AChE: 832 compounds; ∆G = −13.8…−8.1 kcal/mol; LE = 0.35…0.66;
- BACE1: 1633 compounds; ∆G = −12.9…−8.0 kcal/mol; LE = 0.35…0.53;
- GSK3β: 470 compounds; ∆G = −10.3…−8.0 kcal/mol; LE = 0.35…0.46;
- SERT: 1692 compounds; ∆G = −12.5…−8.0 kcal/mol; LE = 0.35…0.51.

The best 60 compounds from each group were selected for additional molecular docking with all other three targets. In result, we found 57 compounds with ligand efficiency for each target greater or equal to 0.4 (Table S2). In this set, 22 compounds satisfy all four conditions of Lipinski’s rules. This group also includes compound ZINC9169727 that was identified as potential multifunctional drug candidates for two targets by virtual screening with Glide VSW module of the Schrödinger suite (Section 2.4).

### 2.6. Selection of Active Compounds

The combination of QSAR methods, two different virtual screening algorithms and molecular docking made it possible to identify 57 potential multifunctional drug candidates with ligand efficiency for each target greater or equal to 0.4 and satisfying at least three of the four conditions of Lipinski’s. Further, the QSAR models were used for a prediction of the biological activity on the selected compounds, i.e., using linear (BMLR) and non-linear (ANN) models. Thus, it was possible to refine the selection of the compounds by using chemical inspection and predicted log (IC_50_) averaged by the QSAR predictions. The range of the predicted biological activities is given in Table 2. Finally, the last filtering of the compounds for future experimental determination of biological activity was carried out considering two conditions: (1) compounds that do not fit the applicability domain were excluded from further analysis; (2) the binding mode to the target protein and the presence of interactions with specific amino acid residues important for target protein activity were taken into account.

To identify the most significant interactions for the biological activity, molecular dynamics simulations for each target protein with known inhibitors were carried out. From Protein Data Bank for each target three crystal structures of the studied receptors in complex with an inhibitor were selected. Therefore, for AChE we used complexes with (−)-huperzine A (IC_50_ = 0.084 μM [29], ID: 4EY5 [30]), donepezil (IC_50_ = 0.013 μM [29], ID: 4EY7 [30]) and (−)-galantamine (IC_50_ = 0.54 μM [31], ID: 4EY6 [30]); for BACE1, complexes with CNP520 (IC_50_ = 0.011 μM, ID: 6EQM [32]), NVP-BXD552 (IC_50_ = 0.002 μM, ID: 4D8C [33]) and *N*-{*N*-[4-(acetylamino)-3.5-dichlorobenzyl]carbamimidoyl}-2- (1H-indol-1-yl)acetamide (VTI, IC_50_ = 1.01 μM, ID: 4IVT [34]); for GSK3β, complexes with BRD0209 (IC_50_ = 0.005 μM, ID: 5KPK [35]), PF-04802367 (IC_50_ = 0.009 μM, ID: 5K5N [36]) and *N*-[4-(isoquinolin-7-yl)pyridin-2-yl]cyclopropanecarboxamide (2WF, IC50 = 0.074 μM, ID: 4PTE [37]); for SERT, complexes with paroxetine (IC_50_ = 0.0021 μM [38], ID: 5I6X [39]), S-citalopram (IC_50_ = 0.01 μM [40], ID: 5I71 [39]) and sertraline (IC_50_ = 0.010 μM [41], ID: 6AWO [42]). According to the molecular dynamics results, it can be assumed that the interaction between the potential inhibitor and the amino acid residues of the catalytic triad of the AChE is not necessary, but the interactions with Trp86, Tyr133, Gly202 or Phe295 can have a significant effect on the activity of the potential inhibitor (Figure S1). For BACE1, the most significant interactions between inhibitors and receptor were interactions with the amino acid residues of catalytic dyad of BACE1 Asp32 and Asp228, and with Gly11, Tyr14, Thr72, Lys107, Phe108 amino acid residues (Figure S2). In the case of GSK3β, the amino acid residues Lys85, Val135 and Arg141 were identified as having an important effect on the activity and the selectivity of the inhibitor. The non-specific contacts with the amino acid residues of the hydrophobic pocket in the ATP-binding domain of GSK3β should also be considered when choosing potential drug candidates, since these amino acid residues are responsible for molecular recognition [43] (Figure S3). The analysis of trajectories of the molecular dynamics simulations of the SERT with selected known inhibitors showed that Tyr95, Asp98, Ile172 and Tyr176 are important determinants of binding at the central active site of SERT and the interaction of potential inhibitors with these amino acid residues should be considered when choosing potential active compounds (Figure S4).

**Figure 4:**
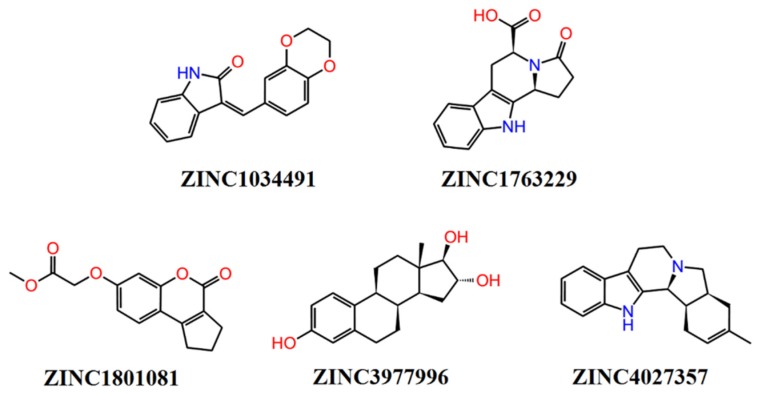
Structures of the selected compounds for biological experiments.

The selection of the potential active compounds was carried out on the basis of molecular docking results which considered only the presence of contact between the small molecule ligand and the key amino acid residue (residues) at the active center of the receptor. The nature of the interaction was later analyzed using molecular dynamics simulations. The structure of the best five compounds selected for the further molecular dynamics simulations study are shown in Figure 4.

**Figure 5:**
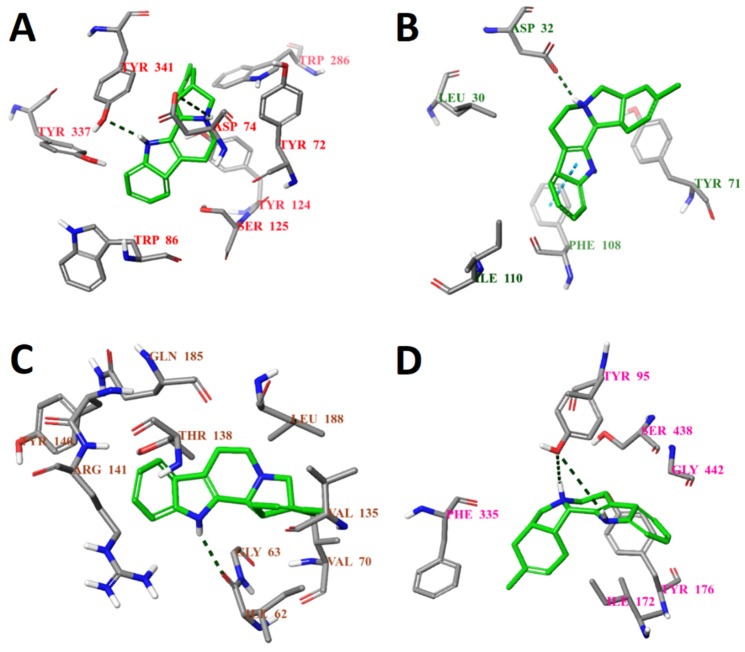
Calculated binding modes of compound ZINC4027357 (**A**) in the active site of AChE (ID: 4EY6); (**B**) in the active site of BACE1 (ID: 6EQM); (**C**) in the active site of GSK3β (ID: 1PYX); (**D**) in the central active site of SERT (ID: 5I6X). The amino acid residues of target proteins are colored as gray (carbon), blue (nitrogen), red (oxygen), and white (hydrogen). Hydrogen bonds formed between compound and residues of target proteins are represented by green dashed lines, and pi–pi stacking is represented by a blue dashed line.

According to the results of the molecular docking, all selected compounds form a contact with at least one key amino acid residue (regarding each target protein). The full analysis of the interactions between the target proteins and small molecule ligands is presented in Table S4 of the Supplementary Materials. The binding poses of compound ZINC4027357 with all protein targets are given in the Figure 5. The binding poses of compounds ZINC1034491 and ZINC3977996 are given in supplementary material (Figures S5 and S6). It should be noted that all selected compounds had the same or better binding energies. Moreover, the ligand efficiencies of the above compounds were comparable to that of the known inhibitors (Table 3).

In order to further analyze the ligand–protein interactions, the molecular dynamics simulations of 50 ns were carried out for the three best compounds with each of the four studied proteins. The dynamic stability of complexes of selected compounds with AChE, BACE1, GSK3β and SERT was evaluated by using the root mean square derivation (RMSD) of the atoms in ligand-protein complexes. The RMSD of all studied ligand-protein complexes was stable between 2.25 and 4.5 (Figures S7–S9).

**Figure 6:**
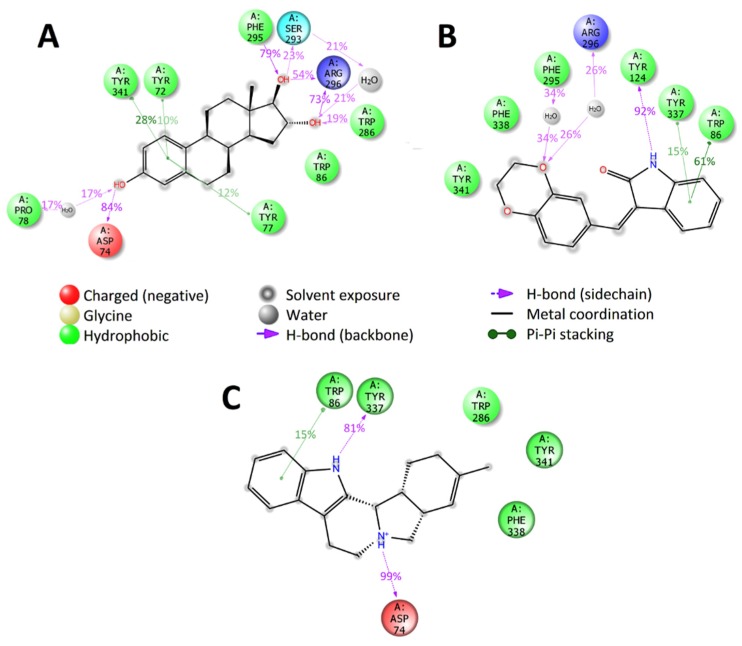
2D summary diagram of molecular dynamics calculated contacts between AChE and small-molecule ligands (**A**) ZINC3977996, (**B**) ZINC1034491 and (**C**) ZINC4027357. Interactions that occur more than 10% of the simulation time are shown.

The molecular dynamics simulations carried out at the active binding site of the studied target proteins indicated similarity in the binding of selected ligands with the binding of the known inhibitors. In the case of the AChE, e.g., all selected compounds bind with AChE at the anionic and peripheral anionic subsites. The compounds ZINC1034491 and ZINC3977996 also form long-term specific interactions with amino acids residues of acyl-binding site of AChE (hydrogen bonds and water bridge with backbone of Phe295), similarly to the most active AChE inhibitor donepezil (Figure 6).

**Figure 7:**
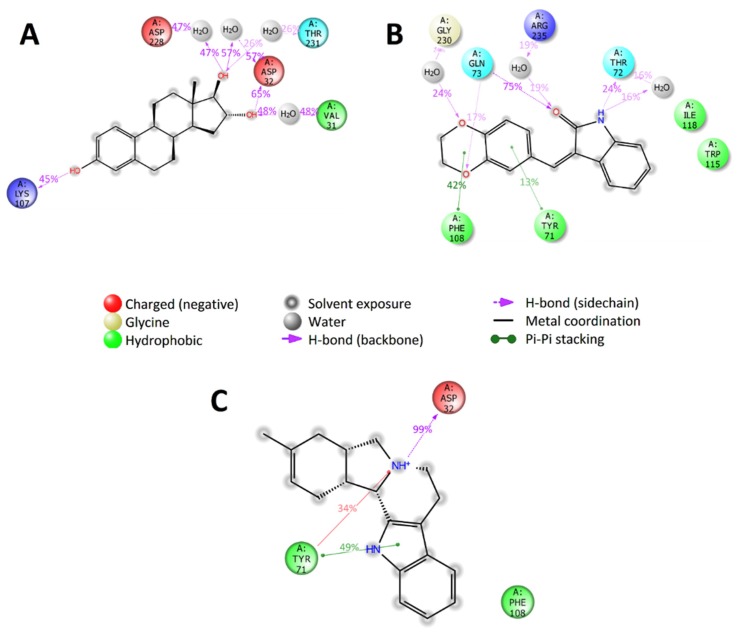
2D summary diagram of molecular dynamics calculated contacts between BACE1 and small-molecule ligands (**A**) ZINC3977996, (**B**) ZINC1034491 and (**C**) ZINC4027357. Interactions that occur more than 10% of the simulation time are shown.

Analysis of trajectories of the molecular dynamics of the complexes of BACE1 with selected compounds shows that compounds ZINC4027357 and ZINC3977996 had strong specific interactions with Asp32 and/or Asp228 amino acids residues of catalytic dyad of BACE1 (Figure 7), and with other important amino acids residues (hydrogen bond with Lys107, pi–pi and pi–cation stacking with Tyr71). The compound ZINC1034491 did not have any contacts with the catalytic dyad of BACE1 throughout of simulation, but had other long-term specific interactions with Tyr71, Phe108 and Gln73 amino acids residues (pi–pi stacking with Tyr71 and Phe108, two hydrogen bonds with a sidechain of Gln73).

**Figure 8:**
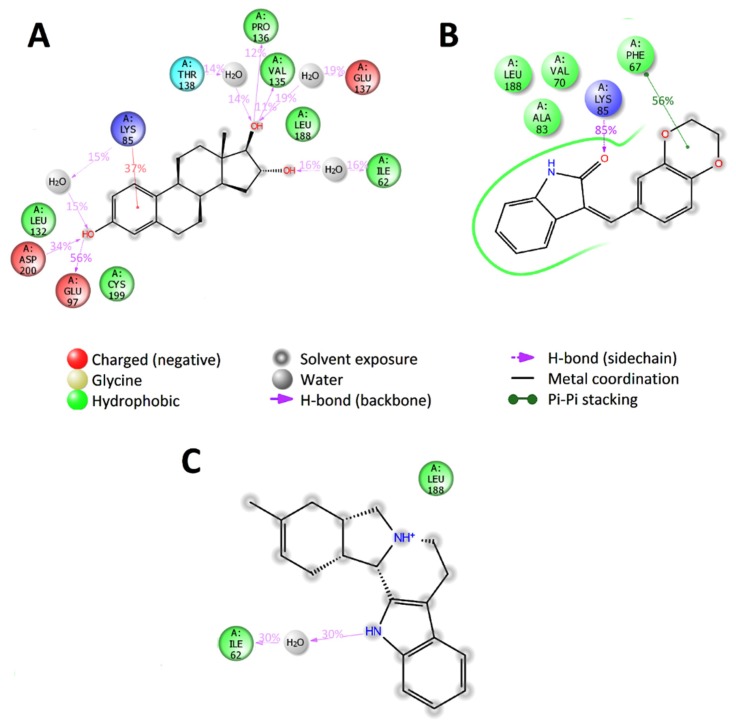
2D summary diagram of molecular dynamics calculated contacts between GSK3β and small-molecule ligands (**A**) ZINC3977996, (**B**) ZINC1034491 and (**C**) ZINC4027357. Interactions that occur more than 10% of the simulation time are shown.

Further, the behavior of the interactions between GSK3β and the three predicted compounds indicated that compounds ZINC1034491 and ZINC3977996 form specific strong interactions with Lys85 and Val135, which have an important effect on the activity and selectivity of inhibitors [43]. In addition, these two compounds have also long-term contacts with amino acids residues of the hydrophobic pocket of the ATP binding domain of GSK3β, which are responsible for molecular recognition [43]. According to the molecular dynamics results, the compound ZINC4027357 binds to GSK3β with the formation of water bridge with the backbone of Ile62 and non-specific hydrophobic contact with Leu188, but does not form any contacts with Lys85 or Val135, that may indicate that this potential inhibitor is not selective towards GSK3β (Figure 8).

**Figure 9:**
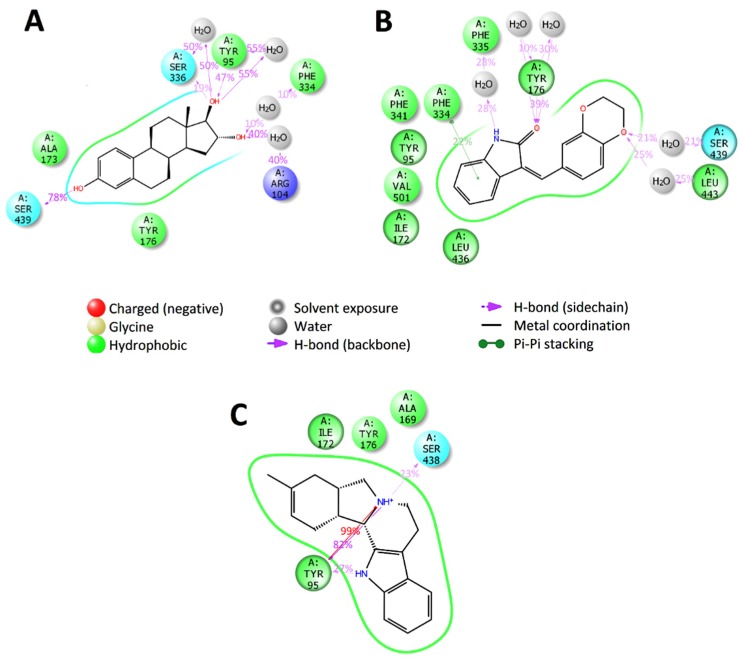
2D summary diagram of molecular dynamics calculated contacts between SERT and small-molecule ligands (**A**) ZINC3977996, (**B**) ZINC1034491 and (**C**) ZINC4027357. Interactions that occur more than 10% of the simulation time are shown.

In the case of SERT, the applicability domain for the model prediction was zero (out of domain) for all selected compounds. Despite this fact, the molecular dynamics results for all the three predicted compounds indicated binding to SERT similar to the known inhibitors (Figure 9). Thus, they form long-term specific contacts with the main binding determinants of the central active site of SERT (Tyr95, Ile172 and Tyr176).

Thus, based on the results of QSAR prediction and molecular modeling study, it can be assumed that compound ZINC3977996 can be tested as a potential multitarget inhibitor for all four target proteins, whilst the other four predicted compounds can be regarded as potential inhibitors of at least three targets. In other words, ZINC1034491 and ZINC1801081 are potential inhibitors for AChE/GSK3β/SERT, and ZINC4027357 is a potential inhibitor for AChE/BACE1/SERT. The compound ZINC1763229 can be regarded as potential inhibitor for GSK3β/SERT.

### 2.7. Enzymatic Assay Results

The ability of the selected compounds to inhibit the activity of the AChE, BACE1, and GSK3β were evaluated using commercially available screening kits (see for detail Section 3.9). Based on the QSAR predictions, the concentrations of compounds for all assays were selected in a range from 0.04 to 25 μM. Among the selected potential inhibitors, two compounds, ZINC4027357 and ZINC1801081, inhibit activity of the AChE (IC_50_ = 0.55 μM and 20.9 μM, respectively). The compound ZINC3977996 also demonstrates inhibitory activity against the AChE at 25 mM concentration and can be tested in a wider range of concentrations. The ability to inhibit the BACE1 activity demonstrates only compound ZINC4027357 (IC_50_ = 5.2 μM). All selected compounds did not inhibit the activity of GSK3β in the tested concentration range and, probably, the range of the tested concentrations should be increased. The obtained values of IC_50_ for compounds ZINC4027357 and ZINC1801081 are given in Supplementary Materials (Figure S10).

## 3. Materials and Methods

### 3.1. Data and Compound Libraries

#### 3.1.1. QSAR Modeling Datasets

In this study, we have developed two types of quantitative structure-activity relationships (QSAR) models based on data for the four receptors, namely AChE, BACE1, GSK3β, SERT. The two types of QSAR models were multilinear regression and nonlinear regression. Therefore, four sets of diverse compounds were extracted from the ChemBL database [44] related to the respective receptors with measured IC_50_ [nM] (see Supplementary Material 1, Table S0). The criteria applied for the selection of the data were: (i) the measured activity values (IC_50_) to fill into the range of at least three log units, (ii) the experimental data to be as recent as possible and, (iii) where possible, the experimental data coming from same laboratory or at least by using the same experimental methodology, (iv) diverse structural compounds with MW < 600 Da, v) compounds with clear structural connectivity, (vi) the IC_50_ values with strong/strange deviations were discarded based on distribution analysis. Thus, it was possible to collect 239, 378, 229 and 213 data points, for AChE, BACE1, GSK3β, and SERT training sets, respectively.

#### 3.1.2. Protein Structures

In order to perform molecular docking analysis for the related proteins and ligands, the three-dimensional structures of the recombinant human acetylcholinesterase (AChE, ID: 4EY6), the beta-secretase 1 (BACE1, ID: 6EQM), the ts3 human serotonin transporter (SERT, ID: 5I6X) and glycogen synthase kinase 3 beta (GSK3β, ID: 1PYX) were obtained from the protein data bank [45,46]. The crystal structures had been measured by X-ray diffraction and resolutions are following: AChE—2.39 Å [30], BACE1—1.35 [32], SERT—3.14 [39], GSK3β—2.4 [47].

### 3.2. Preparation of Protein Target Structures and Compounds Library

The preparation of the target crystal structures was carried out using Schrödinger’s Protein Preparation Wizard of Maestro 10.7 [48,49]. Hydrogen atoms were automatically added to the protein and water molecules were removed from the crystal structure. The two-dimensional structures of selected compounds based on QSAR modeling were downloaded from the ZINC15 database [50]. The three-dimensional coordinates for the ligands were generated using LigPrep from the Schrödinger suite [51]. LigPrep used the OPLS_2005 force field in all ligand preparation steps. All possible states generation and ionization states were enumerated for each ligand using Epik at a pH of 7.0 ± 2. Stereoisomers were determined from 3D structure. PDB files for molecular docking procedure were created from lowest energy conformers for each ligand. PDBQT-files for virtual screening with AutoDock Vina were created using AutoDock Tools 1.5.6 [52].

### 3.3. High-Throughput Virtual Molecular Docking Screening (HTVS)

The virtual screening was carried out based on molecular docking in order to find compounds from the ZINC database with the best docking scores. Two programs were used in this exercise, i.e., Glide Virtual Screening Workflow (VSW) module of the Schrödinger suite 2018 [25] and AutoDock Vina 1.1.2 [27]. The virtual screening with Glide VSW module of the Schrödinger suite was carried out at the three precision levels (HTVS, standard (SP) and extra precision (XP)). The binding interface between the co-crystallized ligand and receptor for each target was identified using Schrödinger’s Glide Grid Generation [53]. The active site of each target during virtual screening with VSW module of the Schrödinger and virtual screening with AutoDock Vina was surrounded with a grid-box sized 20 × 20× 20 Å. All compounds were docked flexibly, and five docking poses were generated for each ligand. Only the best scoring state was kept for the next step of the virtual screening. Thereafter, the virtual screening selected automatically the top 30% of ligands with the best docking score. Finally, the virtual screening resulted in the set of 500 compounds for each target.

The docking parameters of AutoDock Vina were used in their default values (1 CPU to use, exhaustiveness = 8, the number of output poses = 9). The results of the virtual screening with AutoDock Vina were automatically sorted from lowest to highest binding energy. In this way, the selected best compounds possessed binding energy lower than −8.0 kcal/mol.

### 3.4. Molecular Dynamics Simulations

The molecular dynamics calculations using Desmond package of Schrödinger LLC [54] were applied in order to investigate the mechanistic features of the binding of the best ligands selected from the vs. to the target proteins. The simulations were carried out in cubic SPC [55] water box using OPLS_2005 force field parameters [56]. Sodium and chloride ions were placed in the solvent to a concentration 0.15 M, and then, to achieve electro neutrality, additional ions were added to the system. The NPT ensemble with a temperature of 300 K and a pressure of 1 bar was applied in all runs. The simulation length was 50 ns with relaxation time 1 ps for each studied protein-protein complex conformation. The long-range electrostatic interactions were calculated using the particle mesh Ewald (PME) method. The cutoff radius in Coulomb interactions was 9.0 Å. The Martyna-Tuckerman-Klein chain coupling scheme [57] with a coupling constant of 2.0 ps was used for the pressure control and the Nosé-Hoover chain coupling scheme for the temperature control. The non-bonded forces were calculated using a RESPA integrator where the short-range forces were updated every step and the long-range forces were updated every three steps. The trajectories were saved at 50 ps intervals for further analysis. The first 10 ns of molecular dynamics simulations were excluded from further analysis. The behavior and interactions between the small molecule ligands and proteins were analyzed using the Simulation Interaction Diagram tool implemented in the Desmond molecular dynamics package.

### 3.5. Structure Optimizations and Molecular Descriptor Generation of the QSAR Datasets

To ensure the 3D coordinates of the structures of compounds from Section 2.1 were properly generated, several optimization steps were carried out: (1) 2D to 3D conversion using OpenBabel v.2.3 software [58], (2) preliminary structure optimization of the compounds resulted from the previous step by applying molecular mechanics MMFF94s [59] for the search of the best vacuum conformers using the OpenBabel program, (3) semi-empirical quantum mechanics optimization of the structures from the preceding step using AM1 method as encoded in Mopac 6.0 [60]. The obtained structures of the compounds were further submitted for generation of the molecular features (descriptors). All molecular descriptors were generated solely from the molecular structure using FQSARModel v1.0 program [61]. For each single molecule between 600 and 1000 molecular descriptors were calculated. According to the theory used for deriving the descriptors, these can be classified as: (i) constitutional, (ii) geometrical, (iii) topological, (iv) charge-related, (v) quantum chemical, and (vi) thermodynamic [62]. Therefore, it was feasible to generate a very large pool of molecular features containing the physic-chemical information in numerical form for the training sets of Section 3.1.1. The best few descriptors from the pool were used as independent variables in the QSAR equations.

### 3.6. Multilinear Models Based on the BMLR Method

By using the Best Multilinear Regression model (BMLR) [63,64], we were able to generate dozens of multilinear QSAR equations per training set (see Section 3.1.1). The equations related the dependent variable log (IC_50_) to the molecular descriptors as independent variables. In general, the BMLR method is a combination of best feature (descriptor) seeking procedure and simultaneous build of multilinear equations. In other words, the BMLR technique is able to select iteratively the best independent variables among a large pool of descriptors (see Section 3.3) based on R^2^ and F-statistics (see below) and thus to form the 2-descriptor, 3-descriptor etc. n-descriptor multilinear models with highest statistical quality. The final models are selected using criteria as: R^2^—coefficient of determination (squared Pearson’s correlation coefficient), R^2^_cv_ correlation coefficient—cross-validation leave-one-out, R^2^_abc_ correlation coefficient—cross-validation-leave-many-out, s^2^—squared standard deviation, F—Fisher’s criterion.

We used additional cross-validation procedures for the BMLR equations in order to ensure the predictive stability of the models, i.e., the leave-many-out cross-validation called ABC validation [65] and leave-one-out cross-validation. In addition, random scrambling validation of the models was also used.

In order to validate a multilinear model using ABC cross-validation, the data are first sorted in the ascending order according to the dependent variable, and three subsets (A, B, C) were then formed: the 1st, 4th, 7th, etc. data points comprise the first subset (A), the 2nd, 5th, 8th, etc. comprise the second subset (B), and the 3rd, 6th, 9th, etc. comprise the third subset (C). The three training sets were prepared as the combinations of any two subsets (A and B), (A and C), and (B and C), respectively. The tested BMLR model was then rebuilt for each of the training sets with the same descriptors but optimized regression coefficients and used to predict the property values for the respective C, B and A subsets. The prediction was assessed based on the R^2^_abc_ between the predicted and experimental property values. The final result is assessed by the averaged squared correlation coefficient by the three “external” sets A, B and C. The good BMLR models should lead to R^2^_abc_ as high as possible or as close as possible to the original R^2^.

In order to test the equations for “chance descriptors”, the XY-scrambling procedure was applied as follows: (1) y-scrambling: the dependent variable values were scrambled randomly while the descriptors were held unchanged and the new BMLR equation is developed to predict again the log (IC_50_) value with newly obtained regression coefficients, (2) x-scrambling: the dependent variable is held unchanged while randomly permutating the descriptors and the new BMLR equation is used to predict the new log (IC_50_) values and (3) xy-scrambling: the descriptors and dependent variable are changed randomly and on each trial the respective model is used to predict log (IC_50_). For all procedures (1–3), the number of trials was 10,000. Therefore, a low R^2^ from all results indicates a lack of chance correlations.

### 3.7. Nonlinear Models Based on ANN

The original nonlinear regression models were developed based on comprehensive in-house artificial neural network techniques programmed using C++ language. The ANNs possess certain unique attributes for developing QSARs: (1) can learn from examples and can adapt to the change in environmental parameters, (2) because of nonlinear signal processing in ANNs, they are capable of generating highly nonlinear decision boundaries in the multidimensional input space, and (3) they have fault tolerance, such that in case of failure of some of its neurons, the overall performance degrades gracefully [66].

All models developed herein were based on multilayer perceptron with backpropagation of the error using generalized delta rule for learning of the weights [67]. For each data set in Section 3.1.1, more than 20 ANN models were developed with different architectures. The procedure for building a model consisted of the following steps:

(1) *Training and validation sets*: the respective data sets in Section 3.1.1 were divided into training and validation sets and normalized within (−1,1) range. The validation set consists of each 5th data point selected by using the log IC_50_ values ordered in ascending way. This selected set is employed to evaluate the root mean squared error (RMS_val_) or the squared Pearson’s correlation coefficient R^2^_val_ during the training procedure. The RMS_val_ was used as a stopping criterion for the training algorithm, once it started to increase above a certain value.
(2) *Input variable selection:* a smaller pool of statistically significant descriptors was generated from the total pool of descriptors obtained in Section 3.5 by the following manner: (1) the first 10 to 20 descriptors that correlated best with the given activity together with (2) the descriptors obtained from the BMLR models in Section 3.6.
(3) *Network topology:* in order to follow the generality principle for predictability of an ANN model [68], we limited ourselves to architecture with not more than two hidden layers of the nets. By doing this, we also tried to keep the total number of weights as low as possible in order to avoid overparameterizing the network. Thus, networks with the following architectures were considered n-h1-h2-1 or n-h1-1, where n is the number of input descriptors, h1 is the number of neurons in the first hidden layer, h2 is the number of neurons in the second hidden layer and one is the single output neuron in the output layer corresponding to log IC_50_.
(4) *ANN training and best model development* (*BeANN*): we used the following ANN parameters for all models prior the sequential training procedure: learning rate η = 0.1 or 0.2, momentum α = 0.02 and number of training epochs (stopping criterion) not more than 700. For all nets the hidden and output neurons used tanh activation function confined within (−1,1). The initial set of the weights comprised of values between (−1,1) with the closest to zero total mean chosen among 20 random trials. The reason for this is the selection of “good” initial weights that would lead to faster convergence during training procedure.

In the development of a model, a special training procedure was used that attempts to select the best ANN model (BeANN) by selecting (e.g., with two hidden layers) the best 1-h1-h2-1, 2-h1-h2-1, 3-h1-h2-1 etc. n-h1-h2-1 models. This step-wise iterative technique selects networks with highest R^2^_sum_ = R^2^_tr_ + R^2^_val_ (or lowest RMS_val_ + RMS_tr_) within certain number of input descriptors. For example, the BeANN procedure will select the best ANN 1-descriptor model, e.g., 1-h1-h2-1 within a given pool of descriptors. Next, it will use this best input neuron (descriptor) and shuffle the remaining neurons within a given pool of descriptors in order to build the best two-descriptor model (2-h1-h2-1) using the highest R^2^_sum_. Further, these two best descriptors will be held as inputs while a third descriptor will be added iteratively as an input until all descriptors are shuffled within a certain descriptor pool. Thus, the best 3-h1-h2-1 model will be selected with the highest R^2^_sum_. This procedure continues until a certain n is achieved, i.e., the n-h1-h2-1 model is built. Therefore, such an ANN model would possess a statistically high R^2^_tr_ for the training set and a high R^2^_val_ for the validation set.

### 3.8. Virtual Screening Based on the QSAR Models

The QSAR models developed herein were used to predict/indicate logIC_50_ of the best selected compounds refined by the molecular docking models. In conjunction with the prediction of logIC_50_, we used also as a selection criterion the applicability domain (AD) of the QSAR models. The AD was defined by the minimum and maximum descriptor (min–max range) values of the models as extracted by the respective training sets. If any of its descriptor value for prediction of an external compound is out of this min–max range, then its prediction is discarded. However, in order to predict a large number of diverse compounds, we augmented the AD min–max range with 20% for each prediction. Therefore, only compounds that were within this AD were taken into consideration.

### 3.9. Experimental Enzymatic Assays

#### 3.9.1. Compounds

The studied compounds were purchased from MolPort Inc [69]. The 10 mM stock solutions were prepared by dissolving compounds in sterile DMSO (Sigma Aldrich, St. Louis, MO, USA) and stored at –20 °C until further use. All compounds were tested at five concentrations ranging from 0.04 to 25 μM with 5-fold dilution.

#### 3.9.2. Enzymes Inhibition Assays

The inhibitory activity of the selected compounds was evaluated using AChE inhibitor screening colorimetric kit (BioVision), human β-secretase (BACE1) inhibitor fluorometric screening kit (BioVision), and GSK3β assay kit (BPS Bioscience). The assays were carried out following the manufacturer’s protocols. All experiments were carried out in in three parallels in 96-well plates and the readings were obtained using Synergy M microplate reader (BioTek, Winooski, VT, USA) or a GloMax 96-microplate luminometer (Promega, Madison, WI, USA). The 1% DMSO solution was used in all experiments as solvent control; in calculations, the observed value for solvent control was used as 100% activity of enzymes. The AChE inhibitor screening colorimetric kit is based on the ability of an active *h*AChE enzyme to hydrolyze the provided colorimetric substrate, generating a yellow chromophore that can be detected by measuring absorbance at 412 nm. The absorbance was measured immediately in kinetic mode for 40 min at room temperature. In human β-secretase (BACE1) inhibitor fluorometric screening kit, *h*BACE1 cleaves a quenched specific for BACE1 substrate, generating a high fluorescence product, and fluorescence can be detected at Ex/Em = 345/500 nm. The fluorescence was measured in kinetic mode for 90 min at 37 °C. In the GSK3β assay kit, the activity of GSK3β can be measured using a Kinase-Glo assay system (Promega) as a detection reagent. The IC_50_ were calculated by generating dose-response curves using GraphPad Prism version 8.0 for Windows (GraphPad Software, La Jolla, CA, USA) [70].

## 4. Conclusions

In this work, we attempted to discover new potential inhibitors as multitarget compounds simultaneously against AChE, BACE1, SERT and GSK3β proteins. We employed a combination of several computational methods in order to perform this study. Molecular docking models were used to predict a large number of low molecular weight compounds and extract the best candidates. Additionally, artificial neural network and multilinear regression models with high statistical parameters were further used to refine the selected compounds. Thus, the final selection resulted in three new compounds that act as multitarget agents against all four or at least on two or three proteins. The final candidates were submitted to molecular dynamics to assess the most important molecular features related to the ligand-protein interactions.

The final stage of the current study was to experimentally evaluate the best predicted candidates. Notably, our earliest experimental enzymatic assays showed that compound ZINC4027357 was identified as potential inhibitor for at least two targets (AChE and BACE1). Therefore, it can be used for further development of multitarget ligands.

The present work can be used as a framework for future development and discovery of novel multitarget compounds against Alzheimer’s disease.

## Figures and Tables

**Table 1:** Statistical parameters of the BMLR models for all four data sets.

AChE BMLR Model—Log (IC_50_) = D_0_ + ∑(B_i_ ± ErrorsB_i_)*D_i_					
N = 239, R^2^ = 0.932026, R^2^_cv_ = 0.929713, R^2^_abc_ = 0.929519, s^2^ = 0.0808643, F = 802.121					
Descriptor D_i_	B_i_	Errors B_i_	t_i_-Statistics	Descriptors	
0	2.880	0.051	56.989	Intercept	
1	0.312	0.008	38.327	Lowest resonance energy (AM1) for N–S bonds	
2	−2.475	0.184	−13.462	Max net atomic charge (AM1) for O atoms	
3	0.174	0.009	19.494	Final heat of formation (AM1)/# atoms	
4	−0.014	0.001	−14.213	Highest e-e repulsion (AM1) for N–H bonds	
**ABC Crossvalidation**					
(AB,C): R^2^_ab_ =	0.923	R^2^_ab_cv_ =	0.919	R^2^_c_pred_ =	0.944
(BC,A): R^2^_bc_ =	0.932	R^2^_bc_cv_ =	0.929	R^2^_a_pred_ =	0.930
(CA,B): R^2^_ca_ =	0.938	R^2^_ca_cv_ =	0.935	R^2^_b_pred_ =	0.915
**XY-Randomizations**					
X-scrambling R^2^ =				0.0170677	
Y-scrambling R^2^ =				0.0170352	
XY-scrambling R^2^ =				0.0175535	
**BACE1 BMLR Model**					
**N = 378, R^2^ = 0.908694, R^2^_cv_ = 0.90621, R^2^_abc_ = 0.903581, s^2^ = 0.0761742, F = 928.037**					
0	23.461	0.735	31.898	Intercept	
1	0.001	0.000	10.494	PPSA2 Total charge weighted PPSA (AM1)	
2	−0.182	0.003	−55.132	Structural Information content (order 2)	
3	−4.527	0.217	−20.886	Average valency (AM1)	
4	−2.230	0.129	−17.320	Balaban index	
**ABC Crossvalidation**					
(AB,C): R^2^_ab_ =	0.905	R^2^_ab_cv_ =	0.901	R^2^_c_pred_ =	0.909
(BC,A): R^2^_bc_ =	0.912	R^2^_bc_cv_ =	0.908	R^2^_a_pred_ =	0.897
(CA,B): R^2^_ca_ =	0.908	R^2^_ca_cv_ =	0.904	R^2^_b_pred_ =	0.905
**XY-Randomizations**					
X-scrambling R^2^ =				0.0106888	
Y-scrambling R^2^ =				0.0106174	
XY-scrambling R^2^ =				0.0110244	
**GSK3β BMLR Model**					
**N = 229, R^2^ = 0.900783, R^2^_cv_ = 0.896404, R^2^_abc_ = 0.900, s^2^ = 0.0737752, F = 508.42**					
0	6.432	0.142	45.152	Intercept	
1	−0.593	0.017	−34.239	HA dependent HDCA-2 (AM1)	
2	1.569	0.058	27.210	LUMO energy (AM1)	
3	0.710	0.036	19.703	HACA-2 (Zefirov)	
4	11.621	0.694	16.746	Min net atomic charge (Zefirov) for any atom type	
**ABC Crossvalidation**					
(AB,C): R^2^_ab_ =	0.902	R^2^_ab_cv_ =	0.896	R^2^_c_pred_ =	0.899
(BC,A): R^2^_bc_ =	0.900	R^2^_bc_cv_ =	0.893	R^2^_a_pred_ =	0.896
(CA,B): R^2^_ca_ =	0.894	R^2^_ca_cv_ =	0.887	R^2^_b_pred_ =	0.907
**XY-Randomizations**					
X-scrambling R^2^ =				0.0181116	
Y-scrambling R^2^ =				0.0171723	
XY-scrambling R^2^ =				0.0173102	
**SERT BMLR Model**					
**N = 213, R^2^ = 0.846334, R^2^_cv_ = 0.838471, R^2^_abc_ = 0.844291, s^2^ = 0.0767979, F = 286.397**					
0	154.961	10.036	15.440	Intercept	
1	0.598	0.021	27.881	Kier shape index (order 3)	
2	−3.882	0.255	−15.202	Lowest n-n repulsion (AM1)	
3	−0.119	0.005	−22.512	Bonding Information content (order 1)	
4	7.575	0.452	16.740	FHASA Fractional HASA (HASA/TMSA) (AM1)	
**ABC Crossvalidation**					
(AB,C): R^2^_ab_ =	0.839	R^2^_ab_cv_ =	0.826	R^2^_c_pred_ =	0.857
(BC,A): R^2^_bc_ =	0.839	R^2^_bc_cv_ =	0.827	R^2^_a_pred_ =	0.833
(CA,B): R^2^_ca_ =	0.852	R^2^_ca_cv_ =	0.840	R^2^_b_pred_ =	0.843
**XY-Randomizations**					
X-scrambling R^2^ =				0.018852	
Y-scrambling R^2^ =				0.0190767	
XY-scrambling R^2^ =				0.0183609	

**Table 2:** The range of the predicted log (IC_50_) of the selected compounds for all target proteins.

Target	Predicted Log (IC_50_) (nM)			
	ANN		MLR	
	min	max	min	max
AChE	1.975	3.882	3.407	4.082
BACE1	3.149	3.958	2.452	5.280
GSK3β	2.385	4.166	1.548	9.760
SERT	0.567	1.737	−0.770	3.799

**Table 3:** Calculated binding energies (kcal/mol) and binding modes of selected small-molecule ligands and known inhibitors to target proteins (AChE, BACE1, GSK3β and SERT).

Compound	Binding Energy, ∆G, kcal/mol	Ligand Efficiency	Compound	Binding Energy, ∆G, kcal/mol	Ligand Efficiency
**AChE**			**GSK3β**		
(−)-huperzine A	−7.9	0.44	2WF	−7.1	0.32
galantamine	−4.9	0.23	BRD0209	−6.9	0.27
donepezil (DPZ)	−10.2	0.36	PF-04802367	−6.9	0.28
ZINC1034491	−9.8	0.47	ZINC1034491	−8.7	0.41
ZINC4027357	−9.3	0.44	ZINC4027357	−8.6	0.41
ZINC3977996	−9.2	0.44	ZINC3977996	−9.1	0.43
ZINC1763229	−9.2	0.46	ZINC1763229	−8.0	0.40
ZINC1801081	−9.1	0.46	ZINC1801081	−8.1	0.41
**BACE1**			**SERT**		
CNP520	−10.7	0.31	Paroxetine	−10.8	0.45
VTI	−9.9	0.34	S-citalopram	−9.5	0.39
NVP-BXD552	−9.2	0.23	sertraline	−9.1	0.46
ZINC1034491	−10.0	0.48	ZINC1034491	−10.3	0.49
ZINC4027357	−10.0	0.48	ZINC4027357	−10.3	0.49
ZINC3977996	−10.2	0.49	ZINC3977996	−10.1	0.48
ZINC1763229	−8.9	0.45	ZINC1763229	−8.9	0.44
ZINC1801081	−9.6	0.48	ZINC1801081	−8.8	0.44

## Supplementary Materials

The following are available online. Table S0. Experimental and predicted log (IC50) values for all four sets according to the QSAR models Table S1. The results of the virtual screening with VSW module of the Scrödinger suite, Table S2. The results of the virtual screening with AutoDock Vina 1.1.2, Table S3. Predicted log (IC50) values of selected compounds for ANN and BMLR models, Table S4. The analysis of the interactions between receptors and small molecule ligands, Figure S1. 2D summary diagram of molecular dynamics calculated contacts between AChE and inhibitors (A) (-)-huperzine A, (B) (-)-galantamine and (C) donepezil, Figure S2. 2D summary diagram of molecular dynamics calculated contacts between BACE1 and inhibitors (A) NVP-BXD552, (B) CNP520 and (C) VTI, Figure S3. 2D summary diagram of molecular dynamics calculated contacts between GSK3β and inhibitors (A) N-[4-(isoquinolin-7-yl)pyridin-2-yl]cyclopropanecarboxamide, (B) BRD0209 and (C) PF-04802367, Figure S4. 2D summary diagram of molecular dynamics calculated contacts between SERT and inhibitors (A) paroxetine, (B) s-citalopram and (C) sertraline, Figure S5. Calculated binding modes of compound ZINC1034491 (A) in the active site of AChE (ID: 4EY6); (B) in the active site of BACE1 (ID: 6EQM); (C) in the active site of GSK3β (ID: 1PYX); (D) in the central active site of SERT (ID: 5I6X), Figure S6. Calculated binding modes of compound ZINC3977996 (A) in the active site of AChE (ID: 4EY6); (B) in the active site of BACE1 (ID: 6EQM); (C) in the active site of GSK3β (ID: 1PYX); (D) in the central active site of SERT (ID: 5I6X), Figure S7. RMSD of the atomic positions for the compounds ZINC4027357 (in red) and the target proteins (in blue): (A) in the active site of AChE (ID: 4EY6); (B) in the active site of BACE1 (ID: 6EQM); (C) in the active site of GSK3β (ID: 1PYX); (D) in the central active site of SERT (ID: 5I6X) of the 50 ns molecular dynamics simulations, Figure S8. RMSD of the atomic positions for the compounds ZINC1034491 (in red) and the target proteins (in blue): (A) in the active site of AChE (ID: 4EY6); (B) in the active site of BACE1 (ID: 6EQM); (C) in the active site of GSK3β (ID: 1PYX); (D) in the central active site of SERT (ID: 5I6X) of the 50 ns molecular dynamics simulations, Figure S9. RMSD of the atomic positions for the compounds ZINC3977996 (in red) and the target proteins (in blue): (A) in the active site of AChE (ID: 4EY6); (B) in the active site of BACE1 (ID: 6EQM); (C) in the active site of GSK3β (ID: 1PYX); (D) in the central active site of SERT (ID: 5I6X) of the 50 ns molecular dynamics simulations. Figure S10. Inhibition of (A) AChE and (B) BACE1 by ZINC4027357 (IC50 = 0.55 μM and 5.2 μM, respectively); (C) inhibition of AChE by ZINC1801081 (IC_50_ = 20.9 μM).
