# Supplementary material for: Multitarget Approach to Drug Candidates against Alzheimer’s Disease Related to AChE, SERT, BACE1 and GSK3β Protein Targets
Source: Molecules. 2020 Apr 17;25(8):1846. doi: 10.3390/molecules25081846 (PMC7221701; doi:10.3390/molecules25081846)
Supplement: Supplementary file 1 [file molecules-25-01846-s001.pdf]

Supplimentary Material

Table S0. Experimental and predicted log(IC50) for BMLR and ANN QSAR models

BMLR Models											
BACE1			AChE			GSK3b			SERT		
ChemBL ID	Exp log(IC50) [nM]	Pred log(IC50) [nM]	ChemBL ID	Exp log(IC50) [nM]	Pred log(IC50) [nM]	ChemBL ID	Exp log(IC50) [nM]	Pred log(IC50) [nM]	ChemBL ID	Exp log(IC50) [nM]	Pred log(IC50) [nM]
CHEMBL 3695730	-0.301	-0.142	CHEMBL 3585777	-1.571	-1.635	CHEMBL 362558	-0.046	0.136	CHEMBL 3673148	0.176	0.131
CHEMBL 3695751	-0.222	0.156	CHEMBL 3585778	-1.571	-1.533	CHEMBL 3410089	0.041	0.502	CHEMBL 3673174	0.322	0.501
CHEMBL 3695752	-0.155	0.124	CHEMBL 3585779	-1.571	-1.637	CHEMBL 2048685	0.301	0.755	CHEMBL 3673176	0.342	0.625
CHEMBL 3695744	0.146	0.357	CHEMBL 3585775	-1.571	-1.553	CHEMBL 3647955	0.477	0.472	CHEMBL 3673177	0.380	0.241
CHEMBL 3695747	0.146	0.212	CHEMBL 3585776	-1.571	-1.533	CHEMBL 2048659	0.602	0.883	CHEMBL 3673141	0.415	0.512
CHEMBL 3695753	0.301	0.100	CHEMBL 3585782	-1.571	-1.592	CHEMBL 3647956	0.699	0.574	CHEMBL 2333627	0.519	0.896
CHEMBL 3639946	0.380	0.222	CHEMBL 3585783	-1.571	-1.578	CHEMBL 2386106	0.724	0.930	CHEMBL 3673152	0.531	0.824
CHEMBL 3953199	0.415	0.768	CHEMBL 3585784	-1.571	-1.574	CHEMBL 2386112	0.724	0.718	CHEMBL 3673161	0.580	0.580
CHEMBL 3695749	0.447	0.364	CHEMBL 3585780	-1.571	-1.435	CHEMBL 3410110	0.771	0.582	CHEMBL 2338046	0.600	0.780
CHEMBL 3985433	0.699	0.355	CHEMBL 3585781	-1.571	-1.492	CHEMBL 2048683	0.845	1.164	CHEMBL 2333626	0.602	0.776
CHEMBL 3910332	0.699	0.804	CHEMBL 3884988	1.639	1.477	CHEMBL 2048665	0.903	0.912	CHEMBL 3673157	0.623	0.719
CHEMBL 3960603	0.699	1.148	CHEMBL 603622	1.653	1.448	CHEMBL 3647954	0.903	0.671	CHEMBL 3673154	0.633	0.757
CHEMBL 3906568	0.699	0.772	CHEMBL 3827432	2.037	2.552	CHEMBL 2048679	0.903	1.267	CHEMBL 3673175	0.643	0.795
CHEMBL 3394212	0.778	0.931	CHEMBL 3416998	2.072	2.498	CHEMBL 2048672	0.954	0.859	CHEMBL 3323184	0.643	0.977

CHEMBL 3971416	0.799	1.147	CHEMBL 3786937	2.121	2.637	CHEMBL 2048673	0.954	1.413	CHEMBL 3323183	0.653	0.951
CHEMBL 3394217	0.813	0.897	CHEMBL 3410955	2.157	2.620	CHEMBL 3735176	0.982	1.165	CHEMBL 3673167	0.681	0.774
CHEMBL 3394040	0.869	1.055	CHEMBL 3632990	2.176	2.636	CHEMBL 2048669	1.000	1.231	CHEMBL 3703728	0.681	0.925
CHEMBL 3673214	0.875	0.908	CHEMBL 3754672	2.301	2.706	CHEMBL 2386273	1.049	1.232	CHEMBL 3703738	0.690	0.997
CHEMBL 3980437	0.903	0.855	CHEMBL 1677	2.312	2.489	CHEMBL 2386089	1.061	1.010	CHEMBL 3323182	0.690	0.949
CHEMBL 3897568	0.903	0.823	CHEMBL 3632988	2.322	2.665	CHEMBL 2048671	1.079	1.051	CHEMBL 2338053	0.700	0.882
CHEMBL 3964240	0.903	1.062	CHEMBL 3828696	2.330	2.664	CHEMBL 3091536	1.079	1.143	CHEMBL 2338050	0.700	0.909
CHEMBL 3892262	0.954	0.525	CHEMBL 3586207	2.342	2.808	CHEMBL 2048661	1.079	1.205	CHEMBL 3673146	0.716	0.563
CHEMBL 3920257	0.954	0.715	CHEMBL 3752384	2.346	2.710	CHEMBL 3401116	1.079	1.498	CHEMBL 3673143	0.724	0.568
CHEMBL 3905872	0.954	1.023	CHEMBL 3416999	2.346	2.531	CHEMBL 1809110	1.100	1.217	CHEMBL 3323174	0.724	1.050
CHEMBL 3957841	0.954	0.714	CHEMBL 3410956	2.355	2.613	CHEMBL 3410087	1.114	0.708	CHEMBL 3673136	0.748	0.737
CHEMBL 3677269	0.954	1.031	CHEMBL 3819083	2.369	2.531	CHEMBL 3091537	1.114	1.516	CHEMBL 2333608	0.748	0.910
CHEMBL 3695738	0.968	0.445	CHEMBL 3577522	2.398	2.816	CHEMBL 3652540	1.114	1.505	CHEMBL 3673164	0.771	1.008
CHEMBL 3741314	0.978	1.512	CHEMBL 3828054	2.407	2.766	CHEMBL 2048666	1.146	1.596	CHEMBL 3703736	0.785	1.154
CHEMBL 3944362	0.987	0.420	CHEMBL 3415573	2.412	2.628	CHEMBL 2048658	1.146	1.511	CHEMBL 2333648	0.799	1.195
CHEMBL 3980315	1.000	0.902	CHEMBL 3632987	2.415	2.650	CHEMBL 3401120	1.146	1.454	CHEMBL 2338051	0.800	0.872
CHEMBL 3977154	1.013	1.363	CHEMBL 3582232	2.425	2.836	CHEMBL 2386097	1.176	1.473	CHEMBL 3673169	0.806	0.624
CHEMBL 3905497	1.029	1.172	CHEMBL 3415568	2.428	2.781	CHEMBL 3647950	1.204	1.221	CHEMBL 3673150	0.806	0.621
CHEMBL 3916731	1.041	0.887	CHEMBL 3415643	2.431	2.527	CHEMBL 3401125	1.204	1.487	CHEMBL 3323102	0.806	1.167

CHEMBL 3921481	1.041	0.997	CHEMBL 3586577	2.477	2.505	CHEMBL 2046466	1.230	1.009	CHEMBL 2333625	0.806	0.524
CHEMBL 3896933	1.041	1.028	CHEMBL 3415560	2.481	2.922	CHEMBL 2048664	1.230	1.470	CHEMBL 2333624	0.820	1.064
CHEMBL 3688816	1.064	1.229	CHEMBL 3753706	2.489	2.592	CHEMBL 3401115	1.230	1.499	CHEMBL 2333647	0.820	1.255
CHEMBL 3891061	1.079	0.700	CHEMBL 3819231	2.507	2.545	CHEMBL 2391102	1.230	1.435	CHEMBL 2333610	0.833	1.057
CHEMBL 3683816	1.111	1.562	CHEMBL 3417000	2.507	2.544	CHEMBL 2386108	1.246	1.306	CHEMBL 2333604	0.833	1.071
CHEMBL 3673211	1.146	1.448	CHEMBL 3754480	2.509	2.572	CHEMBL 3401119	1.279	1.393	CHEMBL 3703735	0.839	1.197
CHEMBL 3896538	1.146	1.136	CHEMBL 3415565	2.517	2.770	CHEMBL 2391101	1.279	1.475	CHEMBL 3323109	0.839	1.154
CHEMBL 3683804	1.152	1.604	CHEMBL 3582201	2.522	2.859	CHEMBL 3401126	1.279	1.512	CHEMBL 3673170	0.857	0.650
CHEMBL 3394045	1.155	1.529	CHEMBL 3819383	2.530	2.524	CHEMBL 2386102	1.297	1.285	CHEMBL 3673151	0.857	0.805
CHEMBL 3688819	1.158	1.441	CHEMBL 3415575	2.581	2.625	CHEMBL 1809109	1.300	1.583	CHEMBL 3673149	0.863	0.501
CHEMBL 3912215	1.164	1.059	CHEMBL 3597057	2.591	3.039	CHEMBL 3652541	1.301	1.339	CHEMBL 3673142	0.863	1.260
CHEMBL 3973523	1.164	1.212	CHEMBL 3827064	2.591	2.599	CHEMBL 3652550	1.301	1.137	CHEMBL 2333617	0.863	1.025
CHEMBL 3913223	1.176	0.897	CHEMBL 3818089	2.591	2.682	CHEMBL 3647951	1.322	0.850	CHEMBL 2333639	0.869	1.170
CHEMBL 3688660	1.215	1.568	CHEMBL 85251	2.605	2.529	CHEMBL 3736035	1.322	1.378	CHEMBL 3673138	0.892	0.797
CHEMBL 3688820	1.230	1.341	CHEMBL 3754146	2.619	2.510	CHEMBL 489422	1.324	1.372	CHEMBL 2338045	0.900	1.206
CHEMBL 3394054	1.241	1.240	CHEMBL 3752451	2.627	2.602	CHEMBL 2386095	1.330	1.457	CHEMBL 2338031	0.900	1.093
CHEMBL 3688652	1.265	1.591	CHEMBL 3577517	2.633	2.867	CHEMBL 3410105	1.342	1.743	CHEMBL 2337596	0.900	1.303
CHEMBL 3673202	1.279	1.631	CHEMBL 3415570	2.640	2.952	CHEMBL 3091529	1.342	1.598	CHEMBL 3673178	0.903	0.724
CHEMBL 3683911	1.283	1.705	CHEMBL 3586583	2.643	3.123	CHEMBL 3401121	1.362	1.455	CHEMBL 2333615	0.919	1.050

CHEMBL 3683883	1.330	1.523	CHEMBL 3582225	2.653	3.081	CHEMBL 3940448	1.380	1.795	CHEMBL 3323176	0.924	1.128
CHEMBL 3688678	1.338	1.781	CHEMBL 3410957	2.656	2.618	CHEMBL 2386107	1.401	1.003	CHEMBL 3673159	0.934	0.918
CHEMBL 3427250	1.342	1.696	CHEMBL 3905695	2.690	2.752	CHEMBL 3091547	1.431	1.574	CHEMBL 3673173	0.949	0.679
CHEMBL 3683884	1.362	1.522	CHEMBL 3582228	2.693	2.793	CHEMBL 3410109	1.477	1.378	CHEMBL 3681359	0.954	1.301
CHEMBL 3394228	1.362	1.105	CHEMBL 3582224	2.695	2.838	CHEMBL 3647952	1.477	1.387	CHEMBL 2333642	0.954	1.117
CHEMBL 3943188	1.362	1.346	CHEMBL 3415561	2.695	2.901	CHEMBL 3652547	1.477	1.004	CHEMBL 2333614	0.959	1.146
CHEMBL 3947671	1.362	1.357	CHEMBL 3577510	2.699	2.865	CHEMBL 3661031	1.477	1.851	CHEMBL 3673160	0.968	1.334
CHEMBL 3683920	1.367	1.437	CHEMBL 3582206	2.705	2.808	CHEMBL 3401122	1.477	1.514	CHEMBL 3673129	0.973	0.750
CHEMBL 3937729	1.380	1.282	CHEMBL 3597068	2.708	2.749	CHEMBL 3735967	1.491	1.580	CHEMBL 3673162	0.991	0.535
CHEMBL 3695734	1.386	0.999	CHEMBL 3818689	2.721	2.669	CHEMBL 1834324	1.491	1.531	CHEMBL 3673179	1.000	0.677
CHEMBL 3688736	1.394	1.758	CHEMBL 3628184	2.724	3.216	CHEMBL 3401130	1.544	1.494	CHEMBL 3703730	1.000	1.398
CHEMBL 3688815	1.398	1.607	CHEMBL 470867	2.732	2.713	CHEMBL 2391100	1.544	1.620	CHEMBL 2333635	1.000	1.203
CHEMBL 3688812	1.400	1.202	CHEMBL 3752555	2.736	2.634	CHEMBL 2386105	1.553	1.328	CHEMBL 2333645	1.000	1.018
CHEMBL 3394052	1.403	1.604	CHEMBL 3415566	2.736	2.760	CHEMBL 1940906	1.568	2.023	CHEMBL 2337602	1.000	1.144
CHEMBL 3394215	1.415	1.214	CHEMBL 3597056	2.740	2.899	CHEMBL 3410088	1.568	1.087	CHEMBL 2337605	1.000	1.135
CHEMBL 3683833	1.428	1.801	CHEMBL 3416995	2.761	2.605	CHEMBL 2386103	1.599	1.408	CHEMBL 3639720	1.037	0.992
CHEMBL 3688636	1.438	1.655	CHEMBL 3415576	2.762	2.624	CHEMBL 2064536	1.613	1.939	CHEMBL 3770663	1.041	1.428
CHEMBL 3683866	1.439	1.626	CHEMBL 3623561	2.762	3.109	CHEMBL 3401124	1.613	1.611	CHEMBL 3673130	1.053	0.680
CHEMBL 3683802	1.442	1.838	CHEMBL 3628186	2.763	3.221	CHEMBL 2386099	1.614	1.514	CHEMBL 3681356	1.079	1.437

CHEMBL 3688738	1.464	1.723	CHEMBL 3623553	2.772	2.599	CHEMBL 2048667	1.643	1.238	CHEMBL 3681366	1.079	1.350
CHEMBL 3683844	1.468	1.722	CHEMBL 395280	2.778	3.222	CHEMBL 3922494	1.646	1.899	CHEMBL 2333636	1.079	1.378
CHEMBL 3427252	1.477	1.531	CHEMBL 3764305	2.785	3.066	CHEMBL 1834117	1.653	1.803	CHEMBL 2333633	1.079	1.447
CHEMBL 3804970	1.491	1.184	CHEMBL 3410953	2.792	2.638	CHEMBL 3891139	1.663	1.927	CHEMBL 2407334	1.079	1.461
CHEMBL 3688708	1.491	1.608	CHEMBL 3916769	2.799	2.651	CHEMBL 2048657	1.672	1.837	CHEMBL 2338034	1.100	1.112
CHEMBL 3683843	1.509	1.744	CHEMBL 3415580	2.814	2.893	CHEMBL 1801633	1.672	1.467	CHEMBL 2337591	1.100	1.039
CHEMBL 3688627	1.511	1.604	CHEMBL 435071	2.814	3.134	CHEMBL 3652544	1.681	1.454	CHEMBL 2333605	1.114	0.947
CHEMBL 3688822	1.525	1.441	CHEMBL 3582226	2.819	3.005	CHEMBL 3960209	1.690	2.106	CHEMBL 3703733	1.146	1.529
CHEMBL 3634126	1.531	1.882	CHEMBL 3415581	2.836	2.888	CHEMBL 1834326	1.690	2.099	CHEMBL 2333616	1.146	1.452
CHEMBL 3673230	1.544	1.899	CHEMBL 3828443	2.846	2.683	CHEMBL 3661029	1.699	1.595	CHEMBL 3703747	1.149	1.499
CHEMBL 3639876	1.547	1.735	CHEMBL 3818512	2.875	2.760	CHEMBL 3091546	1.708	1.718	CHEMBL 3681358	1.176	1.468
CHEMBL 3683856	1.551	1.457	CHEMBL 3818801	2.878	3.306	CHEMBL 3950215	1.713	1.973	CHEMBL 1852422	1.199	1.467
CHEMBL 3797680	1.556	1.793	CHEMBL 129837	2.881	2.993	CHEMBL 2391098	1.748	1.567	CHEMBL 2338048	1.200	0.869
CHEMBL 3673204	1.556	1.159	CHEMBL 416	2.881	2.719	CHEMBL 1834329	1.756	2.081	CHEMBL 2338035	1.200	1.052
CHEMBL 3969309	1.556	1.741	CHEMBL 3582229	2.890	2.794	CHEMBL 1809107	1.800	1.494	CHEMBL 3703742	1.201	1.322
CHEMBL 3683889	1.559	1.463	CHEMBL 3586576	2.898	3.145	CHEMBL 3736223	1.806	1.952	CHEMBL 1852617	1.204	1.332
CHEMBL 3683915	1.563	1.652	CHEMBL 3623555	2.904	3.025	CHEMBL 1834123	1.813	2.322	CHEMBL 2333607	1.204	0.774
CHEMBL 3683919	1.563	1.439	CHEMBL 3900401	2.911	3.128	CHEMBL 3401114	1.826	1.476	CHEMBL 3673139	1.212	1.343
CHEMBL 3980733	1.568	1.868	CHEMBL 3785861	2.930	2.867	CHEMBL 3736086	1.845	1.799	CHEMBL 1852466	1.225	1.496

CHEMBL 3688665	1.576	1.853	CHEMBL 3819531	2.931	3.243	CHEMBL 2391119	1.875	2.332	CHEMBL 1852359	1.230	1.511
CHEMBL 3688813	1.592	1.368	CHEMBL 3921061	2.940	3.129	CHEMBL 1834325	1.875	1.642	CHEMBL 3769872	1.255	1.482
CHEMBL 3683878	1.597	1.757	CHEMBL 3582227	2.942	2.893	CHEMBL 3091530	1.886	1.595	CHEMBL 3323177	1.255	1.043
CHEMBL 3798544	1.602	1.699	CHEMBL 3597061	2.944	3.241	CHEMBL 1809113	1.900	1.677	CHEMBL 2407326	1.255	1.382
CHEMBL 3688619	1.602	1.941	CHEMBL 3944652	2.946	3.196	CHEMBL 3938281	1.903	1.873	CHEMBL 3673165	1.276	0.790
CHEMBL 3688737	1.669	1.774	CHEMBL 3582209	2.949	2.702	CHEMBL 567958	1.906	1.949	CHEMBL 1852387	1.281	1.406
CHEMBL 3688656	1.671	1.782	CHEMBL 3577520	2.954	2.820	CHEMBL 2386104	1.915	1.516	CHEMBL 2337598	1.300	0.912
CHEMBL 3688643	1.671	1.809	CHEMBL 3586199	2.959	3.010	CHEMBL 3652543	1.919	1.541	CHEMBL 3703734	1.301	1.530
CHEMBL 3673222	1.672	1.297	CHEMBL 3586594	2.959	3.305	CHEMBL 2391099	1.919	2.021	CHEMBL 2407325	1.322	1.388
CHEMBL 3693230	1.681	1.695	CHEMBL 3827220	2.965	2.766	CHEMBL 2391132	1.924	1.898	CHEMBL 3703726	1.330	1.708
CHEMBL 3394042	1.695	1.627	CHEMBL 3414597	2.972	2.665	CHEMBL 3926696	1.928	1.886	CHEMBL 3310489	1.362	1.540
CHEMBL 3746637	1.699	2.190	CHEMBL 3764100	2.973	3.426	CHEMBL 2064518	1.934	2.316	CHEMBL 3331479	1.362	1.316
CHEMBL 3683872	1.699	1.874	CHEMBL 3415564	2.974	2.777	CHEMBL 2391120	1.934	1.952	CHEMBL 2333641	1.362	1.373
CHEMBL 3688824	1.701	1.776	CHEMBL 3577508	2.978	2.938	CHEMBL 3410103	1.954	2.315	CHEMBL 3952992	1.371	1.703
CHEMBL 3683847	1.704	1.476	CHEMBL 3410952	2.978	2.644	CHEMBL 2391129	1.954	2.193	CHEMBL 3323089	1.380	1.594
CHEMBL 3683879	1.706	1.825	CHEMBL 3586592	2.978	3.322	CHEMBL 1834119	2.000	2.280	CHEMBL 2338040	1.400	1.242
CHEMBL 3798199	1.708	2.352	CHEMBL 3910830	2.991	2.743	CHEMBL 1809118	2.000	1.961	CHEMBL 3703729	1.415	1.022
CHEMBL 3693236	1.712	1.989	CHEMBL 3950130	2.991	3.192	CHEMBL 1834121	2.041	2.337	CHEMBL 3817915	1.431	1.623
CHEMBL 3688728	1.718	1.911	CHEMBL 3965783	2.996	2.676	CHEMBL 2386275	2.057	2.177	CHEMBL 3703749	1.446	1.166

CHEMBL 3683805	1.744	1.472	CHEMBL 227075	3.000	3.196	CHEMBL 1738953	2.100	1.915	CHEMBL 3323178	1.447	0.964
CHEMBL 3683826	1.754	2.128	CHEMBL 3586600	3.000	3.280	CHEMBL 1738951	2.100	2.408	CHEMBL 3323093	1.462	1.497
CHEMBL 3640259	1.763	2.245	CHEMBL 3586574	3.000	3.158	CHEMBL 2391126	2.104	2.486	CHEMBL 3703740	1.473	1.258
CHEMBL 3673198	1.778	1.931	CHEMBL 3819320	3.004	2.826	CHEMBL 2064539	2.111	2.275	CHEMBL 3681357	1.477	1.608
CHEMBL 3427249	1.778	1.942	CHEMBL 3763203	3.009	3.482	CHEMBL 2064517	2.114	2.115	CHEMBL 3323094	1.491	1.388
CHEMBL 3688617	1.785	1.902	CHEMBL 3582231	3.016	2.873	CHEMBL 3093051	2.114	2.381	CHEMBL 3323105	1.491	1.701
CHEMBL 3936217	1.790	2.170	CHEMBL 3827284	3.019	2.667	CHEMBL 2062563	2.114	1.807	CHEMBL 2337594	1.500	1.304
CHEMBL 3683811	1.797	1.830	CHEMBL 3763232	3.029	3.210	CHEMBL 3699443	2.127	1.643	CHEMBL 3323106	1.505	1.618
CHEMBL 3688632	1.805	2.006	CHEMBL 3945423	3.037	2.739	CHEMBL 2386274	2.170	1.652	CHEMBL 2333613	1.505	1.133
CHEMBL 3673196	1.806	2.026	CHEMBL 3892044	3.049	2.841	CHEMBL 3093052	2.176	2.250	CHEMBL 2407331	1.505	1.515
CHEMBL 3673231	1.813	2.309	CHEMBL 3765697	3.061	3.411	CHEMBL 2390958	2.185	1.735	CHEMBL 3593274	1.544	1.834
CHEMBL 3673206	1.833	1.846	CHEMBL 3828281	3.070	2.775	CHEMBL 1809120	2.200	1.957	CHEMBL 3323097	1.544	1.247
CHEMBL 3427247	1.845	2.004	CHEMBL 3402718	3.075	3.528	CHEMBL 1738952	2.200	2.363	CHEMBL 3593396	1.556	2.043
CHEMBL 3746261	1.845	2.226	CHEMBL 3582230	3.084	2.842	CHEMBL 1834316	2.204	2.164	CHEMBL 3310485	1.563	1.292
CHEMBL 3683845	1.864	1.975	CHEMBL 3763778	3.097	3.441	CHEMBL 1834328	2.204	2.209	CHEMBL 2407327	1.568	1.627
CHEMBL 3673226	1.869	2.547	CHEMBL 611941	3.100	3.110	CHEMBL 2391133	2.215	1.784	CHEMBL 3310487	1.574	1.560
CHEMBL 3672718	1.898	2.302	CHEMBL 3827011	3.114	3.412	CHEMBL 2391128	2.215	1.937	CHEMBL 3310490	1.585	1.204
CHEMBL 3683876	1.906	2.013	CHEMBL 3628065	3.114	3.213	CHEMBL 2064519	2.220	2.003	CHEMBL 3703750	1.591	2.036
CHEMBL 3688821	1.908	1.456	CHEMBL 3402719	3.115	3.518	CHEMBL 355496	2.241	2.268	CHEMBL 3798035	1.602	1.383

CHEMBL 3683917	1.909	1.534	CHEMBL 1254269	3.117	3.054	CHEMBL 2064531	2.246	2.585	CHEMBL 3323096	1.602	1.216
CHEMBL 3673203	1.914	1.656	CHEMBL 19215	3.128	3.058	CHEMBL 3933780	2.255	2.119	CHEMBL 1852373	1.629	1.808
CHEMBL 3673228	1.919	2.072	CHEMBL 3930722	3.130	2.792	CHEMBL 2064535	2.267	2.044	CHEMBL 3770651	1.643	2.000
CHEMBL 3683873	1.921	2.013	CHEMBL 3577509	3.134	2.942	CHEMBL 1834120	2.279	2.032	CHEMBL 3331498	1.651	1.940
CHEMBL 3683877	1.922	1.815	CHEMBL 3765093	3.143	3.350	CHEMBL 2391117	2.290	2.303	CHEMBL 3310484	1.666	1.275
CHEMBL 3683881	1.927	2.227	CHEMBL 3818992	3.161	2.683	CHEMBL 3736164	2.301	2.444	CHEMBL 3310483	1.673	1.487
CHEMBL 3683916	1.938	1.698	CHEMBL 3958208	3.170	2.865	CHEMBL 3093047	2.301	2.418	CHEMBL 3317694	1.699	1.597
CHEMBL 3683858	1.941	2.230	CHEMBL 3961103	3.176	3.641	CHEMBL 3661030	2.301	1.910	CHEMBL 1852774	1.714	1.824
CHEMBL 3933659	1.949	2.257	CHEMBL 3818230	3.176	3.448	CHEMBL 3652546	2.301	1.760	CHEMBL 3703725	1.731	1.630
CHEMBL 3918935	1.949	2.409	CHEMBL 3586614	3.176	2.936	CHEMBL 2391130	2.352	2.250	CHEMBL 3310486	1.764	1.554
CHEMBL 3688654	1.952	2.297	CHEMBL 3818167	3.179	3.307	CHEMBL 3734956	2.362	2.869	CHEMBL 3797621	1.778	1.762
CHEMBL 3688719	1.956	2.324	CHEMBL 3763820	3.188	3.370	CHEMBL 2064537	2.367	2.291	CHEMBL 3703745	1.786	1.373
CHEMBL 3688640	1.958	1.790	CHEMBL 3824239	3.188	3.296	CHEMBL 2391123	2.369	2.643	CHEMBL 2380972	1.806	1.722
CHEMBL 3688612	1.963	2.069	CHEMBL 3818415	3.188	3.278	CHEMBL 3410099	2.380	2.776	CHEMBL 1852516	1.820	1.590
CHEMBL 3640275	1.968	1.546	CHEMBL 3819155	3.188	3.281	CHEMBL 1940904	2.398	2.514	CHEMBL 3818471	1.826	1.476
CHEMBL 3634124	1.968	2.195	CHEMBL 3787116	3.207	2.703	CHEMBL 3735962	2.398	2.555	CHEMBL 3323092	1.826	1.510
CHEMBL 3688695	1.971	1.713	CHEMBL 2163795	3.228	3.642	CHEMBL 1940908	2.398	2.230	CHEMBL 1852361	1.827	1.864
CHEMBL 3683808	1.987	2.306	CHEMBL 481	3.262	3.136	CHEMBL 3735613	2.415	2.021	CHEMBL 1852777	1.852	1.628
CHEMBL 3683855	1.987	1.907	CHEMBL 3402708	3.263	3.558	CHEMBL 1834115	2.462	2.614	CHEMBL 3310478	1.857	1.441

CHEMBL 3688722	1.996	1.655	CHEMBL 489354	3.265	2.940	CHEMBL 3661032	2.477	2.243	CHEMBL 2407335	1.898	1.813
CHEMBL 3683874	1.996	1.951	CHEMBL 171048	3.281	3.200	CHEMBL 2022413	2.477	2.523	CHEMBL 1852472	1.914	1.602
CHEMBL 3688615	1.999	2.144	CHEMBL 524100	3.288	2.790	CHEMBL 2022415	2.477	2.685	CHEMBL 1852659	1.924	1.965
CHEMBL 3688664	2.017	1.956	CHEMBL 3973337	3.297	3.231	CHEMBL 3734985	2.491	2.414	CHEMBL 3818953	1.929	1.466
CHEMBL 3688616	2.028	2.291	CHEMBL 3402713	3.299	3.406	CHEMBL 3647953	2.491	2.428	CHEMBL 2430680	1.932	1.664
CHEMBL 3673197	2.029	2.068	CHEMBL 3577524	3.301	2.809	CHEMBL 2064515	2.500	2.203	CHEMBL 3323107	1.982	1.732
CHEMBL 3650826	2.037	2.162	CHEMBL 3415579	3.303	2.906	CHEMBL 1809117	2.500	2.179	CHEMBL 3593395	1.987	2.046
CHEMBL 3427253	2.041	2.284	CHEMBL 2071425	3.303	3.225	CHEMBL 1809122	2.500	2.494	CHEMBL 2380979	1.987	2.019
CHEMBL 3903848	2.059	2.352	CHEMBL 3828028	3.322	3.416	CHEMBL 1809121	2.500	2.484	CHEMBL 2430681	1.997	1.909
CHEMBL 3693233	2.062	2.066	CHEMBL 3415559	3.323	2.934	CHEMBL 3735529	2.519	2.003	CHEMBL 3331507	2.000	1.642
CHEMBL 3422244	2.064	2.188	CHEMBL 3597006	3.332	3.202	CHEMBL 3736462	2.544	2.646	CHEMBL 3703741	2.003	2.053
CHEMBL 3688729	2.070	1.800	CHEMBL 3819646	3.334	3.435	CHEMBL 3943352	2.551	2.654	CHEMBL 3612833	2.033	2.353
CHEMBL 3683789	2.070	2.089	CHEMBL 221753	3.351	3.752	CHEMBL 3734911	2.556	2.708	CHEMBL 3331484	2.057	1.647
CHEMBL 3688620	2.077	1.692	CHEMBL 3582212	3.384	3.470	CHEMBL 1834113	2.643	2.659	CHEMBL 3593394	2.079	2.010
CHEMBL 3747766	2.079	2.109	CHEMBL 3632852	3.398	2.863	CHEMBL 3093042	2.699	2.991	CHEMBL 2407330	2.079	1.661
CHEMBL 3683857	2.085	2.180	CHEMBL 3623566	3.398	3.441	CHEMBL 2024372	2.699	2.546	CHEMBL 3126963	2.114	1.999
CHEMBL 3688709	2.088	2.160	CHEMBL 2071424	3.418	3.431	CHEMBL 3735772	2.724	2.434	CHEMBL 3331505	2.114	1.779
CHEMBL 3688626	2.094	2.224	CHEMBL 3582210	3.430	2.968	CHEMBL 454662	2.724	3.172	CHEMBL 2407332	2.114	1.705
CHEMBL 3683892	2.097	2.114	CHEMBL 3628059	3.431	3.877	CHEMBL 3735095	2.724	2.638	CHEMBL 2333622	2.146	2.209

CHEMBL 3688663	2.099	2.585	CHEMBL 334255	3.440	3.133	CHEMBL 3735445	2.733	3.068	CHEMBL 2347692	2.146	2.169
CHEMBL 3683904	2.106	2.100	CHEMBL 3597060	3.446	3.187	CHEMBL 3736122	2.748	2.445	CHEMBL 2380974	2.146	1.780
CHEMBL 3650831	2.107	1.926	CHEMBL 3926911	3.456	3.099	CHEMBL 3736196	2.748	2.550	CHEMBL 3593400	2.176	2.522
CHEMBL 3693228	2.109	1.996	CHEMBL 496	3.479	2.994	CHEMBL 3736396	2.763	2.681	CHEMBL 2347855	2.176	2.406
CHEMBL 3683885	2.116	1.824	CHEMBL 636	3.481	3.263	CHEMBL 3736418	2.763	2.489	CHEMBL 3681361	2.215	1.768
CHEMBL 3683801	2.129	2.066	CHEMBL 145361	3.493	3.147	CHEMBL 1801635	2.763	2.870	CHEMBL 2347856	2.230	2.484
CHEMBL 3703303	2.137	1.923	CHEMBL 3976003	3.502	3.886	CHEMBL 1834110	2.778	2.753	CHEMBL 3126977	2.279	2.641
CHEMBL 3650849	2.140	2.360	CHEMBL 1555	3.505	3.134	CHEMBL 2022418	2.778	2.924	CHEMBL 3764774	2.301	2.029
CHEMBL 3688724	2.151	1.898	CHEMBL 3628056	3.505	3.796	CHEMBL 3410102	2.785	3.311	CHEMBL 3593392	2.301	2.147
CHEMBL 3688625	2.154	2.275	CHEMBL 3984034	3.508	3.843	CHEMBL 1809115	2.800	2.370	CHEMBL 3593275	2.301	2.166
CHEMBL 3703285	2.155	1.635	CHEMBL 3765569	3.511	3.481	CHEMBL 3735755	2.806	3.126	CHEMBL 1201066	2.310	1.994
CHEMBL 3683905	2.155	1.690	CHEMBL 3763609	3.517	3.362	CHEMBL 3736404	2.826	2.604	CHEMBL 3126961	2.322	2.490
CHEMBL 3693229	2.161	2.005	CHEMBL 3972214	3.519	3.855	CHEMBL 3735293	2.839	2.353	CHEMBL 3331468	2.430	2.000
CHEMBL 3688635	2.163	1.943	CHEMBL 3818332	3.519	3.145	CHEMBL 3408213	2.839	2.848	CHEMBL 3799356	2.431	1.998
CHEMBL 3673205	2.176	1.934	CHEMBL 3628062	3.519	3.346	CHEMBL 3410098	2.863	3.142	CHEMBL 3799596	2.447	2.494
CHEMBL 3960008	2.176	2.522	CHEMBL 3765476	3.528	3.458	CHEMBL 3901767	2.876	2.515	CHEMBL 3799668	2.447	2.039
CHEMBL 3688621	2.179	2.271	CHEMBL 3628058	3.531	3.797	CHEMBL 1682842	2.903	2.847	CHEMBL 3126967	2.447	2.811
CHEMBL 3703233	2.188	2.318	CHEMBL 3949439	3.543	3.013	CHEMBL 483841	2.903	3.062	CHEMBL 3799795	2.477	2.217
CHEMBL 3910646	2.204	2.218	CHEMBL 3763973	3.549	3.498	CHEMBL 2022417	2.903	3.297	CHEMBL 3906576	2.486	2.398

CHEMBL 3959813	2.204	2.399	CHEMBL 3822890	3.551	3.363	CHEMBL 3673435	2.933	2.748	CHEMBL 3612835	2.509	2.394
CHEMBL 3654232	2.207	2.471	CHEMBL 3763482	3.569	3.541	CHEMBL 3735497	2.940	2.544	CHEMBL 3593391	2.519	2.129
CHEMBL 3688659	2.217	2.530	CHEMBL 3822559	3.580	3.340	CHEMBL 1834331	2.954	2.500	CHEMBL 3334801	2.528	2.106
CHEMBL 3654216	2.262	2.640	CHEMBL 3628063	3.580	3.467	CHEMBL 3763944	2.954	3.284	CHEMBL 2407321	2.531	2.291
CHEMBL 3688721	2.264	2.052	CHEMBL 3764679	3.587	3.519	CHEMBL 2022419	2.954	2.928	CHEMBL 3334797	2.578	2.983
CHEMBL 3688609	2.267	2.809	CHEMBL 3586605	3.591	3.380	CHEMBL 2022421	2.954	2.543	CHEMBL 3818345	2.580	2.611
CHEMBL 3683882	2.267	1.912	CHEMBL 3628057	3.591	3.796	CHEMBL 3673434	2.956	2.938	CHEMBL 3334776	2.590	2.787
CHEMBL 3683922	2.280	2.702	CHEMBL 3765496	3.598	3.597	CHEMBL 3951486	3.013	3.011	CHEMBL 3334799	2.601	2.964
CHEMBL 3683898	2.289	2.608	CHEMBL 3892118	3.613	3.744	CHEMBL 2022420	3.041	3.113	CHEMBL 3334794	2.603	2.564
CHEMBL 3688707	2.291	2.188	CHEMBL 3628055	3.623	3.950	CHEMBL 2024374	3.041	2.678	CHEMBL 3798279	2.623	2.173
CHEMBL 3654219	2.297	2.486	CHEMBL 3770206	3.663	3.525	CHEMBL 2022423	3.041	3.268	CHEMBL 3612825	2.625	3.050
CHEMBL 3986498	2.362	2.278	CHEMBL 3823464	3.666	3.318	CHEMBL 3735382	3.079	2.620	CHEMBL 3334793	2.652	2.345
CHEMBL 3427246	2.362	2.329	CHEMBL 1262	3.667	3.847	CHEMBL 2024371	3.079	2.771	CHEMBL 3593404	2.681	2.521
CHEMBL 3586197	2.362	2.459	CHEMBL 3765162	3.686	3.589	CHEMBL 3093040	3.114	3.145	CHEMBL 3334800	2.700	2.906
CHEMBL 3683903	2.380	2.235	CHEMBL 3822670	3.694	3.307	CHEMBL 3093036	3.114	2.872	CHEMBL 3331504	2.714	2.431
CHEMBL 3673217	2.384	2.935	CHEMBL 577016	3.708	3.972	CHEMBL 2024359	3.114	3.123	CHEMBL 3126959	2.716	2.716
CHEMBL 3688752	2.385	2.818	CHEMBL 3623565	3.715	3.396	CHEMBL 3911190	3.161	2.946	CHEMBL 3126972	2.724	2.261
CHEMBL 3688690	2.390	2.625	CHEMBL 653	3.749	3.486	CHEMBL 151	3.176	3.246	CHEMBL 3334798	2.737	3.151
CHEMBL 3650822	2.394	2.020	CHEMBL 3752227	3.752	3.210	CHEMBL 2312290	3.225	3.031	CHEMBL 3799481	2.748	2.258

CHEMBL 3984088	2.398	2.169	CHEMBL 3628060	3.756	3.873	CHEMBL 3787112	3.255	3.070	CHEMBL 2347691	2.756	2.676
CHEMBL 3683860	2.401	2.621	CHEMBL 3770356	3.763	3.336	CHEMBL 2022422	3.255	2.774	CHEMBL 3963788	2.778	2.369
CHEMBL 3693257	2.402	2.484	CHEMBL 64894	3.767	3.637	CHEMBL 28	3.279	3.376	CHEMBL 2347695	2.820	2.380
CHEMBL 3634338	2.410	2.465	CHEMBL 1801816	3.771	3.514	CHEMBL 3093044	3.279	3.006	CHEMBL 3593405	2.833	2.522
CHEMBL 3654221	2.415	2.847	CHEMBL 3763417	3.775	3.512	CHEMBL 50	3.301	3.444	CHEMBL 3126965	2.833	2.725
CHEMBL 3688717	2.433	2.219	CHEMBL 3763832	3.777	3.429	CHEMBL 2024361	3.301	2.973	CHEMBL 3763998	2.903	2.422
CHEMBL 3703234	2.441	2.709	CHEMBL 3586602	3.778	3.429	CHEMBL 3765061	3.303	3.120	CHEMBL 3126969	2.987	2.706
CHEMBL 3688662	2.452	2.044	CHEMBL 1327885	3.792	3.554	CHEMBL 3763318	3.320	3.239	CHEMBL 2347703	2.987	2.746
CHEMBL 3688745	2.463	2.361	CHEMBL 239618	3.799	3.547	CHEMBL 3093037	3.322	2.973	CHEMBL 281594	3.584	3.520
CHEMBL 3688716	2.478	2.407	CHEMBL 3764065	3.804	3.562	CHEMBL 3093046	3.342	3.025			
CHEMBL 3654215	2.479	2.878	CHEMBL 3957099	3.806	3.700	CHEMBL 3093038	3.380	2.926			
CHEMBL 3688718	2.496	2.099	CHEMBL 1079905	3.806	3.367	CHEMBL 3763728	3.396	3.212			
CHEMBL 3654223	2.500	2.754	CHEMBL 3770071	3.806	3.374	CHEMBL 464381	3.410	3.712			
CHEMBL 3703255	2.500	2.442	CHEMBL 1200623	3.813	3.357	CHEMBL 3763815	3.444	3.165			
CHEMBL 3673191	2.505	2.436	CHEMBL 3764900	3.819	3.461	CHEMBL 2313853	3.519	3.138			
CHEMBL 3688747	2.530	2.556	CHEMBL 3764488	3.827	3.500	CHEMBL 2313850	3.602	3.259			
CHEMBL 3703281	2.542	2.694	CHEMBL 3764433	3.850	3.518	CHEMBL 3298402	3.664	3.504			
CHEMBL 3650825	2.543	2.631	CHEMBL 240047	3.851	3.935	CHEMBL 102953	3.699	4.136			
CHEMBL 3677240	2.568	3.000	CHEMBL 113	3.860	3.826	CHEMBL 3765319	3.745	3.801			

CHEMBL 3673201	2.575	2.368	CHEMBL 394753	3.869	4.029	CHEMBL 219346	3.760	3.832			
CHEMBL 3650833	2.589	2.475	CHEMBL 305660	3.874	3.493	CHEMBL 218301	3.790	3.993			
CHEMBL 3703266	2.589	2.439	CHEMBL 3770516	3.875	3.969	CHEMBL 140	4.254	3.954			
CHEMBL 3650772	2.590	2.922	CHEMBL 3764714	3.887	3.399	CHEMBL 1668411	4.255	4.190			
CHEMBL 3765050	2.591	2.790	CHEMBL 3824057	3.895	3.367	CHEMBL 2334786	4.630	4.596			
CHEMBL 3654228	2.591	2.486	CHEMBL 3770866	3.898	3.927	CHEMBL 105712	4.699	4.265			
CHEMBL 3693239	2.598	2.727	CHEMBL 239400	3.903	4.046						
CHEMBL 3764532	2.602	2.554	CHEMBL 3586604	3.929	3.409						
CHEMBL 3650774	2.603	2.915	CHEMBL 1801815	3.996	4.004						
CHEMBL 3672721	2.614	3.041	CHEMBL 3786666	4.130	3.638						
CHEMBL 3673227	2.616	2.844	CHEMBL 808	4.136	3.779						
CHEMBL 3752926	2.633	2.550	CHEMBL 91	4.271	3.767						
CHEMBL 3969436	2.639	2.402	CHEMBL 3628061	4.360	4.055						
CHEMBL 3654226	2.642	2.794	CHEMBL 3628054	4.456	3.862						
CHEMBL 3654229	2.647	2.838	CHEMBL 3628053	4.484	4.001						
CHEMBL 3654230	2.669	2.726	CHEMBL 426113	5.223	5.226						
CHEMBL 3654218	2.675	2.354									
CHEMBL 3746896	2.681	2.618									
CHEMBL 3677236	2.681	2.778									

CHEMBL 3693253	2.699	2.483									
CHEMBL 3890737	2.708	2.348									
CHEMBL 3422235	2.708	2.869									
CHEMBL 3672708	2.713	3.053									
CHEMBL 3688691	2.720	2.650									
CHEMBL 3650801	2.769	2.293									
CHEMBL 3422239	2.777	2.757									
CHEMBL 3900541	2.778	2.497									
CHEMBL 3693256	2.781	2.472									
CHEMBL 3677231	2.785	2.506									
CHEMBL 3672414	2.799	2.556									
CHEMBL 3650768	2.803	2.550									
CHEMBL 3747445	2.806	2.659									
CHEMBL 3703288	2.817	2.286									
CHEMBL 3745922	2.820	3.114									
CHEMBL 3650820	2.820	2.889									
CHEMBL 3747444	2.826	2.432									
CHEMBL 3747381	2.857	2.333									
CHEMBL 3747247	2.857	2.558									

CHEMBL 3765711	2.863	2.936									
CHEMBL 3677226	2.869	2.475									
CHEMBL 3703290	2.869	2.431									
CHEMBL 3650782	2.880	2.841									
CHEMBL 3650769	2.888	2.634									
CHEMBL 3672413	2.898	2.496									
CHEMBL 3422240	2.903	2.800									
CHEMBL 3672410	2.908	2.911									
CHEMBL 3688668	2.914	2.979									
CHEMBL 3691360	2.917	3.498									
CHEMBL 3703272	2.918	2.648									
CHEMBL 3677217	2.919	2.702									
CHEMBL 3650798	2.939	2.955									
CHEMBL 3688611	2.940	2.893									
CHEMBL 3650814	2.944	2.919									
CHEMBL 3691362	2.961	3.340									
CHEMBL 3677208	2.964	2.836									
CHEMBL 3650790	2.968	2.652									
CHEMBL 3650777	2.970	2.947									

CHEMBL 3763944	2.987	2.818									
CHEMBL 3650807	2.997	2.528									
CHEMBL 3703220	3.004	3.275									
CHEMBL 3764465	3.017	3.075									
CHEMBL 3703236	3.025	2.769									
CHEMBL 3703301	3.025	3.132									
CHEMBL 3650795	3.027	2.740									
CHEMBL 3763264	3.033	2.799									
CHEMBL 3672419	3.041	2.838									
CHEMBL 3672442	3.079	2.317									
CHEMBL 3672436	3.079	3.048									
CHEMBL 3672408	3.079	2.900									
CHEMBL 3672401	3.079	3.005									
CHEMBL 3677228	3.079	3.102									
CHEMBL 3672426	3.079	3.180									
CHEMBL 3672425	3.079	2.679									
CHEMBL 3691376	3.093	3.381									
CHEMBL 3703239	3.093	3.169									
CHEMBL 3650775	3.100	2.901									

CHEMBL 3672417	3.114	2.904									
CHEMBL 3891320	3.114	3.005									
CHEMBL 3752467	3.130	3.161									
CHEMBL 3414701	3.143	3.362									
CHEMBL 3672439	3.146	2.986									
CHEMBL 3672404	3.146	3.154									
CHEMBL 3677218	3.146	2.231									
CHEMBL 3672440	3.146	2.980									
CHEMBL 3677242	3.146	3.020									
CHEMBL 3650813	3.161	3.078									
CHEMBL 3703259	3.170	2.876									
CHEMBL 3703261	3.170	2.851									
CHEMBL 3677220	3.176	2.984									
CHEMBL 3672411	3.176	2.949									
CHEMBL 3703209	3.193	2.718									
CHEMBL 3672407	3.204	3.111									
CHEMBL 3672447	3.204	3.263									
CHEMBL 3672415	3.204	3.312									
CHEMBL 3703296	3.207	2.996									

CHEMBL 3928706	3.220	3.026										
CHEMBL 3650770	3.223	2.881										
CHEMBL 3672406	3.230	3.481										
CHEMBL 3677244	3.230	3.034										
CHEMBL 3703300	3.262	2.793										
CHEMBL 3677227	3.279	3.332										
CHEMBL 3672421	3.279	3.050										
CHEMBL 3703279	3.281	2.760										
CHEMBL 3672443	3.301	3.225										
CHEMBL 3672418	3.301	3.195										
CHEMBL 3672402	3.322	3.040										
CHEMBL 3688803	3.322	3.495										
CHEMBL 3703297	3.340	3.128										
CHEMBL 3672416	3.342	3.110										
CHEMBL 3691383	3.356	3.393										
CHEMBL 3763815	3.358	3.368										
CHEMBL 3677222	3.362	3.677										
CHEMBL 3672424	3.362	3.078										
CHEMBL 3650757	3.364	3.076										

CHEMBL 3703251	3.367	3.107										
CHEMBL 3672433	3.380	2.848										
CHEMBL 3677234	3.380	2.838										
CHEMBL 3672432	3.415	3.347										
CHEMBL 3703294	3.418	3.162										
CHEMBL 3765061	3.430	3.569										
CHEMBL 3677238	3.431	3.296										
CHEMBL 3688795	3.438	3.736										
CHEMBL 1405147	3.462	3.004										
CHEMBL 3703241	3.462	3.267										
CHEMBL 3672431	3.477	3.288										
CHEMBL 3677219	3.491	3.913										
CHEMBL 3650767	3.515	3.238										
CHEMBL 3703315	3.530	3.450										
CHEMBL 3677229	3.531	2.739										
CHEMBL 3672405	3.531	3.191										
CHEMBL 3691365	3.572	3.719										
CHEMBL 3672430	3.602	3.188										
CHEMBL 3703221	3.660	3.829										

CHEMBL 3672717	3.688	3.862									
CHEMBL 3414706	3.708	3.877									
CHEMBL 3688688	3.713	3.574									
CHEMBL 3691370	3.728	3.727									
CHEMBL 3688672	3.730	3.142									
CHEMBL 3765319	3.781	3.714									
CHEMBL 50	3.813	3.648									
CHEMBL 3917763	3.827	3.461									
CHEMBL 3703215	3.858	3.935									
CHEMBL 3691363	3.876	4.205									
CHEMBL 3691364	3.900	3.974									
CHEMBL 3691379	3.925	3.524									
CHEMBL 3691369	4.009	3.766									
CHEMBL 3691366	4.017	4.389									
CHEMBL 3703210	4.068	3.939									
CHEMBL 3414705	4.236	4.182									
CHEMBL 3752684	4.256	4.602									
CHEMBL 3586138	4.336	4.446									
CHEMBL 3703245	4.394	4.293									

CHEMBL 3703240	4.415	3.843									
CHEMBL 3703222	4.480	4.396									
CHEMBL 3703206	4.775	4.519									
**ANN Models**											
CHEMBL 3695730	-0.301	0.119	CHEMBL 3585777	-1.570	-0.507	CHEMBL 362558	-0.046	0.345	CHEMBL 3673148	0.176	0.332
CHEMBL 3695753	0.301	0.187	CHEMBL 3585782	-1.570	-1.446	CHEMBL 3647956	0.699	0.714	CHEMBL 2333627	0.519	0.776
CHEMBL 3910332	0.699	0.925	CHEMBL 3884988	1.640	4.062	CHEMBL 2048665	0.903	0.672	CHEMBL 3673157	0.623	0.600
CHEMBL 3394217	0.813	1.337	CHEMBL 3410955	2.160	2.790	CHEMBL 3735176	0.982	1.392	CHEMBL 3673167	0.681	0.640
CHEMBL 3964240	0.903	1.142	CHEMBL 3828696	2.330	2.927	CHEMBL 3091536	1.080	1.093	CHEMBL 2338050	0.700	0.788
CHEMBL 3677269	0.954	1.409	CHEMBL 3819083	2.370	2.676	CHEMBL 3091537	1.110	1.463	CHEMBL 2333608	0.748	0.786
CHEMBL 3977154	1.010	1.542	CHEMBL 3582232	2.420	3.364	CHEMBL 2386097	1.180	1.243	CHEMBL 3673169	0.806	0.537
CHEMBL 3688816	1.060	1.235	CHEMBL 3753706	2.490	2.854	CHEMBL 3401115	1.230	1.424	CHEMBL 2333647	0.820	1.185
CHEMBL 3683804	1.150	1.732	CHEMBL 3582201	2.520	3.384	CHEMBL 3401126	1.280	1.555	CHEMBL 3673170	0.857	0.552
CHEMBL 3913223	1.180	0.939	CHEMBL 3818089	2.590	2.904	CHEMBL 3647951	1.320	0.633	CHEMBL 2333639	0.869	1.082
CHEMBL 3673202	1.280	1.432	CHEMBL 3415570	2.640	3.170	CHEMBL 3091529	1.340	1.472	CHEMBL 3673178	0.903	0.600
CHEMBL 3683884	1.360	1.636	CHEMBL 3582228	2.690	3.319	CHEMBL 3410109	1.480	0.863	CHEMBL 3681359	0.954	1.185
CHEMBL 3937729	1.380	1.361	CHEMBL 3597068	2.710	3.064	CHEMBL 3735967	1.490	1.898	CHEMBL 3673162	0.991	0.485
CHEMBL 3394052	1.400	1.994	CHEMBL 3415566	2.740	2.865	CHEMBL 1940906	1.570	1.473	CHEMBL 2337602	1.000	1.054
CHEMBL 3683802	1.440	1.636	CHEMBL 3628186	2.760	3.502	CHEMBL 2386099	1.610	1.153	CHEMBL 3681356	1.080	1.350
CHEMBL	1.490	1.761	CHEMBL	2.800	2.895	CHEMBL	1.670	1.992	CHEMBL	1.100	1.010

3688708			3916769			2048657			2338034		
CHEMBL 3673230	1.540	1.984	CHEMBL 3828443	2.850	2.955	CHEMBL 3661029	1.700	1.468	CHEMBL 3703747	1.150	1.344
CHEMBL 3969309	1.560	1.790	CHEMBL 3582229	2.890	3.321	CHEMBL 1809107	1.800	1.455	CHEMBL 3703742	1.200	1.213
CHEMBL 3688665	1.580	1.708	CHEMBL 3819531	2.930	3.422	CHEMBL 2391119	1.880	1.776	CHEMBL 1852359	1.230	1.488
CHEMBL 3688737	1.670	1.752	CHEMBL 3582209	2.950	3.242	CHEMBL 567958	1.910	1.696	CHEMBL 1852387	1.280	1.326
CHEMBL 3394042	1.690	1.694	CHEMBL 3414597	2.970	2.887	CHEMBL 3926696	1.930	1.781	CHEMBL 3310489	1.360	1.595
CHEMBL 3683879	1.710	1.664	CHEMBL 3586592	2.980	3.522	CHEMBL 1834119	2.000	1.415	CHEMBL 2338040	1.400	1.194
CHEMBL 3683826	1.750	2.054	CHEMBL 3586600	3.000	3.526	CHEMBL 1738951	2.100	2.044	CHEMBL 3323093	1.460	1.389
CHEMBL 3936217	1.790	2.232	CHEMBL 3827284	3.020	2.950	CHEMBL 2062563	2.110	1.575	CHEMBL 2337594	1.500	1.267
CHEMBL 3673206	1.830	1.749	CHEMBL 3828281	3.070	3.098	CHEMBL 1809120	2.200	1.466	CHEMBL 3323097	1.540	1.095
CHEMBL 3672718	1.900	2.734	CHEMBL 3827011	3.110	3.463	CHEMBL 2391128	2.210	1.345	CHEMBL 3310490	1.590	1.140
CHEMBL 3673228	1.920	2.492	CHEMBL 3930722	3.130	3.183	CHEMBL 2064535	2.270	1.833	CHEMBL 3770651	1.640	2.237
CHEMBL 3683858	1.940	2.237	CHEMBL 3961103	3.180	3.653	CHEMBL 3661030	2.300	1.790	CHEMBL 1852774	1.710	1.959
CHEMBL 3688640	1.960	1.735	CHEMBL 3824239	3.190	3.393	CHEMBL 2391123	2.370	2.160	CHEMBL 2380972	1.810	1.671
CHEMBL 3683808	1.990	2.382	CHEMBL 481	3.260	3.363	CHEMBL 3735613	2.410	1.647	CHEMBL 1852777	1.850	1.674
CHEMBL 3688664	2.020	1.838	CHEMBL 3973337	3.300	3.503	CHEMBL 3734985	2.490	2.566	CHEMBL 3818953	1.930	1.432
CHEMBL 3903848	2.060	2.508	CHEMBL 3828028	3.320	3.472	CHEMBL 1809121	2.500	1.973	CHEMBL 2430681	2.000	2.079
CHEMBL 3688620	2.080	1.518	CHEMBL 3582212	3.380	3.642	CHEMBL 1834113	2.640	1.909	CHEMBL 3593394	2.080	2.226
CHEMBL 3683892	2.100	2.078	CHEMBL 3628059	3.430	3.846	CHEMBL 3735095	2.720	2.309	CHEMBL 2333622	2.150	2.582
CHEMBL	2.120	1.602	CHEMBL	3.480	3.379	CHEMBL	2.760	2.751	CHEMBL	2.210	1.852

3683885			636			3736418			3681361		
CHEMBL 3688625	2.150	2.208	CHEMBL 3984034	3.510	3.738	CHEMBL 1809115	2.800	1.799	CHEMBL 3593275	2.300	2.389
CHEMBL 3673205	2.180	1.803	CHEMBL 3628062	3.520	3.603	CHEMBL 3410098	2.860	2.923	CHEMBL 3799596	2.450	2.834
CHEMBL 3959813	2.200	2.432	CHEMBL 3822890	3.550	3.435	CHEMBL 3673435	2.930	2.914	CHEMBL 3612835	2.510	2.707
CHEMBL 3688609	2.270	2.747	CHEMBL 3586605	3.590	3.561	CHEMBL 2022421	2.950	2.451	CHEMBL 3818345	2.580	2.762
CHEMBL 3654219	2.300	2.634	CHEMBL 3770206	3.660	3.774	CHEMBL 2022423	3.040	2.524	CHEMBL 3612825	2.630	3.113
CHEMBL 3673217	2.380	2.967	CHEMBL 577016	3.710	3.894	CHEMBL 2024359	3.110	2.195	CHEMBL 3126959	2.720	2.951
CHEMBL 3683860	2.400	2.714	CHEMBL 3770356	3.760	3.604	CHEMBL 2022422	3.260	2.252	CHEMBL 3963788	2.780	2.524
CHEMBL 3703234	2.440	2.795	CHEMBL 3586602	3.780	3.611	CHEMBL 3765061	3.300	3.048	CHEMBL 3126969	2.990	2.949
CHEMBL 3688718	2.500	1.953	CHEMBL 1079905	3.810	3.454	CHEMBL 3763728	3.400	3.155	CHEMBL 3673176	0.342	0.498
CHEMBL 3703281	2.540	2.905	CHEMBL 3764433	3.850	3.769	CHEMBL 3298402	3.660	3.193	CHEMBL 3673161	0.580	0.509
CHEMBL 3703266	2.590	2.511	CHEMBL 3770516	3.880	3.962	CHEMBL 140	4.250	3.531	CHEMBL 3673175	0.643	0.594
CHEMBL 3764532	2.600	2.751	CHEMBL 3586604	3.930	3.588	CHEMBL 2048685	0.301	0.625	CHEMBL 3703738	0.690	0.812
CHEMBL 3969436	2.640	2.440	CHEMBL 3628061	4.360	3.988	CHEMBL 2386112	0.724	0.362	CHEMBL 3673143	0.724	0.502
CHEMBL 3746896	2.680	1.954	CHEMBL 3585779	-1.570	-0.387	CHEMBL 2048679	0.903	1.046	CHEMBL 3703736	0.785	0.987
CHEMBL 3672708	2.710	3.131	CHEMBL 3585784	-1.570	-1.530	CHEMBL 2386273	1.050	1.184	CHEMBL 3323102	0.806	0.973
CHEMBL 3693256	2.780	2.381	CHEMBL 3827432	2.040	2.762	CHEMBL 3401116	1.080	1.495	CHEMBL 2333604	0.833	0.958
CHEMBL 3703288	2.820	2.428	CHEMBL 3754672	2.300	3.052	CHEMBL 2048666	1.150	1.555	CHEMBL 3673149	0.863	0.467
CHEMBL 3747247	2.860	2.584	CHEMBL 3752384	2.350	3.081	CHEMBL 3401125	1.200	1.548	CHEMBL 2338045	0.900	1.099
CHEMBL	2.890	2.771	CHEMBL	2.410	3.086	CHEMBL	1.250	1.093	CHEMBL	0.924	0.948

3650769			3828054			2386108			3323176		
CHEMBL 3691360	2.920	3.558	CHEMBL 3415643	2.430	2.384	CHEMBL 1809109	1.300	0.778	CHEMBL 2333614	0.959	1.051
CHEMBL 3650814	2.940	3.133	CHEMBL 3417000	2.510	2.672	CHEMBL 489422	1.320	1.055	CHEMBL 3703730	1.000	1.321
CHEMBL 3763944	2.990	2.954	CHEMBL 3415575	2.580	2.441	CHEMBL 3940448	1.380	1.251	CHEMBL 3639720	1.040	0.835
CHEMBL 3703301	3.030	3.385	CHEMBL 3754146	2.620	2.657	CHEMBL 3652547	1.480	1.200	CHEMBL 2333636	1.080	1.382
CHEMBL 3672436	3.080	3.191	CHEMBL 3582225	2.650	3.501	CHEMBL 3401130	1.540	1.447	CHEMBL 2333605	1.110	0.824
CHEMBL 3672425	3.080	2.863	CHEMBL 3415561	2.700	3.098	CHEMBL 2386103	1.600	1.231	CHEMBL 1852422	1.200	1.432
CHEMBL 3891320	3.110	3.078	CHEMBL 3628184	2.720	3.495	CHEMBL 3922494	1.650	1.658	CHEMBL 2333607	1.200	0.669
CHEMBL 3677218	3.150	2.375	CHEMBL 3416995	2.760	2.780	CHEMBL 3652544	1.680	1.366	CHEMBL 3323177	1.260	0.853
CHEMBL 3703261	3.170	2.995	CHEMBL 395280	2.780	3.349	CHEMBL 3950215	1.710	1.794	CHEMBL 3703734	1.300	1.524
CHEMBL 3672447	3.200	3.430	CHEMBL 435071	2.810	3.389	CHEMBL 1834123	1.810	2.125	CHEMBL 2333641	1.360	1.373
CHEMBL 3672406	3.230	3.744	CHEMBL 3818801	2.880	3.484	CHEMBL 3091530	1.890	1.412	CHEMBL 3817915	1.430	1.683
CHEMBL 3703279	3.280	3.024	CHEMBL 3623555	2.900	3.320	CHEMBL 3652543	1.920	1.424	CHEMBL 3681357	1.480	1.649
CHEMBL 3703297	3.340	3.391	CHEMBL 3582227	2.940	3.401	CHEMBL 2391120	1.930	1.703	CHEMBL 2333613	1.510	1.036
CHEMBL 3672424	3.360	3.261	CHEMBL 3586199	2.960	3.160	CHEMBL 1834121	2.040	1.878	CHEMBL 3310485	1.560	1.265
CHEMBL 3672432	3.410	3.494	CHEMBL 3415564	2.970	2.894	CHEMBL 2064539	2.110	2.055	CHEMBL 3798035	1.600	1.310
CHEMBL 1405147	3.460	3.208	CHEMBL 3950130	2.990	3.453	CHEMBL 2386274	2.170	1.677	CHEMBL 3310484	1.670	1.225
CHEMBL 3703315	3.530	3.622	CHEMBL 3819320	3.000	3.087	CHEMBL 1834316	2.200	1.608	CHEMBL 3310486	1.760	1.632
CHEMBL 3703221	3.660	3.742	CHEMBL 3945423	3.040	3.076	CHEMBL 355496	2.240	2.574	CHEMBL 3818471	1.830	1.343
CHEMBL	3.730	2.976	CHEMBL	3.080	3.369	CHEMBL	2.290	2.122	CHEMBL	1.900	1.959

3688672			3582230			2391117			2407335		
CHEMBL 3691363	3.880	4.099	CHEMBL 3402719	3.110	3.572	CHEMBL 2391130	2.350	2.097	CHEMBL 3323107	1.980	1.746
CHEMBL 3703210	4.070	3.889	CHEMBL 3765093	3.140	3.560	CHEMBL 1940904	2.400	1.829	CHEMBL 3703741	2.000	2.220
CHEMBL 3703240	4.410	3.741	CHEMBL 3586614	3.180	3.271	CHEMBL 3661032	2.480	2.185	CHEMBL 3126963	2.110	2.283
CHEMBL 3695752	-0.155	0.238	CHEMBL 3819155	3.190	3.439	CHEMBL 2064515	2.500	2.364	CHEMBL 2380974	2.150	1.809
CHEMBL 3953199	0.415	0.885	CHEMBL 489354	3.260	3.183	CHEMBL 3736462	2.540	2.753	CHEMBL 3126977	2.280	2.903
CHEMBL 3906568	0.699	0.953	CHEMBL 3577524	3.300	3.195	CHEMBL 2024372	2.700	2.079	CHEMBL 3126961	2.320	2.784
CHEMBL 3673214	0.875	0.988	CHEMBL 3597006	3.330	3.461	CHEMBL 3736122	2.750	2.560	CHEMBL 3126967	2.450	3.004
CHEMBL 3920257	0.954	0.731	CHEMBL 3623566	3.400	3.516	CHEMBL 1834110	2.780	1.946	CHEMBL 3334801	2.530	2.350
CHEMBL 3741314	0.978	0.940	CHEMBL 3597060	3.450	3.401	CHEMBL 3736404	2.830	2.492	CHEMBL 3334799	2.600	3.066
CHEMBL 3916731	1.040	0.864	CHEMBL 3976003	3.500	3.785	CHEMBL 1682842	2.900	2.775	CHEMBL 3593404	2.680	2.791
CHEMBL 3683816	1.110	1.206	CHEMBL 3763609	3.520	3.643	CHEMBL 1834331	2.950	1.838	CHEMBL 3334798	2.740	3.130
CHEMBL 3688819	1.160	1.516	CHEMBL 3628058	3.530	3.829	CHEMBL 3951486	3.010	2.665	CHEMBL 3593405	2.830	2.792
CHEMBL 3688820	1.230	1.412	CHEMBL 3822559	3.580	3.427	CHEMBL 2024371	3.080	2.642	CHEMBL 281594	3.580	3.216
CHEMBL 3683883	1.330	1.580	CHEMBL 3765496	3.600	3.735	CHEMBL 151	3.180	2.958	CHEMBL 3673141	0.415	0.473
CHEMBL 3943188	1.360	1.560	CHEMBL 1262	3.670	4.065	CHEMBL 3093044	3.280	2.486	CHEMBL 2333626	0.602	0.670
CHEMBL 3688736	1.390	1.889	CHEMBL 653	3.750	3.607	CHEMBL 3093037	3.320	2.572	CHEMBL 3323183	0.653	0.766
CHEMBL 3683833	1.430	2.035	CHEMBL 1801816	3.770	3.769	CHEMBL 3763815	3.440	3.105	CHEMBL 2338053	0.700	0.767
CHEMBL 3683844	1.470	1.940	CHEMBL 239618	3.800	3.731	CHEMBL 3765319	3.750	3.748	CHEMBL 3673136	0.748	0.567
CHEMBL	1.510	1.732	CHEMBL	3.810	3.327	CHEMBL	4.630	4.207	CHEMBL	0.800	0.757

3688627			1200623			2334786			2338051		
CHEMBL 3683856	1.550	1.372	CHEMBL 113	3.860	3.784	CHEMBL 3647955	0.477	0.464	CHEMBL 2333624	0.820	0.952
CHEMBL 3683915	1.560	1.357	CHEMBL 3824057	3.890	3.453	CHEMBL 3410110	0.771	0.665	CHEMBL 3323109	0.839	0.977
CHEMBL 3683878	1.600	2.039	CHEMBL 3786666	4.130	3.771	CHEMBL 2048672	0.954	0.747	CHEMBL 2333617	0.863	0.910
CHEMBL 3688643	1.670	1.738	CHEMBL 3628053	4.480	3.918	CHEMBL 2386089	1.060	0.810	CHEMBL 2337596	0.900	1.265
CHEMBL 3683872	1.700	1.666	CHEMBL 3585775	-1.570	0.209	CHEMBL 1809110	1.100	1.416	CHEMBL 3673173	0.949	0.571
CHEMBL 3693236	1.710	1.914	CHEMBL 3585780	-1.570	-0.444	CHEMBL 2048658	1.150	1.002	CHEMBL 3673129	0.973	0.573
CHEMBL 3673198	1.780	2.133	CHEMBL 3416998	2.070	2.371	CHEMBL 2046466	1.230	0.838	CHEMBL 2333645	1.000	0.896
CHEMBL 3688632	1.800	1.875	CHEMBL 1677	2.310	2.826	CHEMBL 3401119	1.280	1.398	CHEMBL 3673130	1.050	0.537
CHEMBL 3746261	1.850	2.082	CHEMBL 3416999	2.350	2.646	CHEMBL 3652541	1.300	1.016	CHEMBL 2407334	1.080	1.472
CHEMBL 3688821	1.910	1.594	CHEMBL 3415573	2.410	2.418	CHEMBL 2386095	1.330	1.251	CHEMBL 2333616	1.150	1.461
CHEMBL 3683877	1.920	1.733	CHEMBL 3586577	2.480	2.419	CHEMBL 2386107	1.400	0.662	CHEMBL 2338035	1.200	0.945
CHEMBL 3918935	1.950	2.487	CHEMBL 3754480	2.510	2.822	CHEMBL 3661031	1.480	1.588	CHEMBL 1852466	1.230	1.452
CHEMBL 3640275	1.970	1.586	CHEMBL 3597057	2.590	3.307	CHEMBL 2391100	1.540	1.416	CHEMBL 3673165	1.280	0.591
CHEMBL 3688722	2.000	1.810	CHEMBL 3752451	2.630	2.871	CHEMBL 2064536	1.610	1.636	CHEMBL 3703726	1.330	1.741
CHEMBL 3673197	2.030	2.301	CHEMBL 3410957	2.660	2.794	CHEMBL 1834117	1.650	1.049	CHEMBL 3323089	1.380	1.533
CHEMBL 3422244	2.060	2.241	CHEMBL 3577510	2.700	3.241	CHEMBL 3960209	1.690	1.706	CHEMBL 3323178	1.450	0.775
CHEMBL 3683857	2.080	2.204	CHEMBL 470867	2.730	3.182	CHEMBL 2391098	1.750	1.414	CHEMBL 3323105	1.490	1.667
CHEMBL 3683904	2.110	2.037	CHEMBL 3415576	2.760	2.491	CHEMBL 3401114	1.830	1.317	CHEMBL 3593274	1.540	1.958
CHEMBL	2.140	2.042	CHEMBL	2.790	3.290	CHEMBL	1.900	1.377	CHEMBL	1.570	1.636

3703303			3764305			1809113			3310487		
CHEMBL 3683905	2.160	1.542	CHEMBL 3582226	2.820	3.459	CHEMBL 2391099	1.920	1.696	CHEMBL 1852373	1.630	1.927
CHEMBL 3688621	2.180	2.225	CHEMBL 129837	2.880	3.272	CHEMBL 3410103	1.950	1.801	CHEMBL 3317694	1.700	1.655
CHEMBL 3688659	2.220	2.667	CHEMBL 3900401	2.910	3.344	CHEMBL 2386275	2.060	1.726	CHEMBL 3703745	1.790	1.238
CHEMBL 3683922	2.280	2.543	CHEMBL 3597061	2.940	3.512	CHEMBL 2064517	2.110	2.258	CHEMBL 1852361	1.830	2.049
CHEMBL 3427246	2.360	2.650	CHEMBL 3586594	2.960	3.501	CHEMBL 3093052	2.180	1.823	CHEMBL 1852659	1.920	2.174
CHEMBL 3688690	2.390	2.769	CHEMBL 3577508	2.980	3.288	CHEMBL 1834328	2.200	1.384	CHEMBL 2380979	1.990	2.154
CHEMBL 3634338	2.410	2.431	CHEMBL 3965783	3.000	2.874	CHEMBL 2064531	2.250	2.475	CHEMBL 3331484	2.060	1.737
CHEMBL 3688745	2.460	2.514	CHEMBL 3763203	3.010	3.698	CHEMBL 3736164	2.300	2.703	CHEMBL 2407332	2.110	1.851
CHEMBL 3703255	2.500	2.710	CHEMBL 3892044	3.050	3.101	CHEMBL 3734956	2.360	2.862	CHEMBL 2347855	2.180	2.754
CHEMBL 3677240	2.570	3.151	CHEMBL 3763778	3.100	3.626	CHEMBL 3735962	2.400	2.867	CHEMBL 3593392	2.300	2.366
CHEMBL 3765050	2.590	2.974	CHEMBL 1254269	3.120	3.257	CHEMBL 2022413	2.480	2.597	CHEMBL 3799356	2.430	2.294
CHEMBL 3672721	2.610	3.140	CHEMBL 3818992	3.160	2.886	CHEMBL 1809117	2.500	1.776	CHEMBL 3906576	2.490	2.752
CHEMBL 3654229	2.650	2.798	CHEMBL 3818167	3.180	3.477	CHEMBL 3943352	2.550	2.181	CHEMBL 3334797	2.580	3.074
CHEMBL 3693253	2.700	2.430	CHEMBL 3787116	3.210	2.964	CHEMBL 3735772	2.720	2.507	CHEMBL 3798279	2.620	2.537
CHEMBL 3650801	2.770	2.632	CHEMBL 171048	3.280	3.415	CHEMBL 3736196	2.750	2.555	CHEMBL 3331504	2.710	2.716
CHEMBL 3672414	2.800	2.746	CHEMBL 3415579	3.300	3.097	CHEMBL 2022418	2.780	2.955	CHEMBL 2347691	2.760	2.958
CHEMBL 3650820	2.820	2.991	CHEMBL 3819646	3.330	3.528	CHEMBL 3735293	2.840	2.284	CHEMBL 3763998	2.900	2.615
CHEMBL 3677226	2.870	2.617	CHEMBL 2071424	3.420	3.543	CHEMBL 483841	2.900	1.721	CHEMBL 3673177	0.380	0.371
CHEMBL	2.900	2.780	CHEMBL	3.460	3.370	CHEMBL	2.950	3.153	CHEMBL	0.600	0.685

3422240			3926911			3763944			2338046		
CHEMBL 3677217	2.920	3.026	CHEMBL 1555	3.510	3.348	CHEMBL 2022420	3.040	3.158	CHEMBL 3323184	0.643	0.790
CHEMBL 3677208	2.960	3.058	CHEMBL 3972214	3.520	3.793	CHEMBL 3093040	3.110	2.713	CHEMBL 3323182	0.690	0.764
CHEMBL 3703220	3.000	3.452	CHEMBL 3949439	3.540	3.298	CHEMBL 2312290	3.230	3.025	CHEMBL 3323174	0.724	0.862
CHEMBL 3763264	3.030	2.921	CHEMBL 3628063	3.580	3.653	CHEMBL 50	3.300	2.954	CHEMBL 2333648	0.799	1.112
CHEMBL 3672401	3.080	3.146	CHEMBL 3892118	3.610	3.705	CHEMBL 3093046	3.340	2.508	CHEMBL 2333625	0.806	0.500
CHEMBL 3703239	3.090	3.476	CHEMBL 3765162	3.690	3.786	CHEMBL 2313853	3.520	2.658	CHEMBL 3703735	0.839	1.040
CHEMBL 3414701	3.140	3.384	CHEMBL 3752227	3.750	3.332	CHEMBL 219346	3.760	3.111	CHEMBL 3673142	0.863	1.048
CHEMBL 3677242	3.150	3.228	CHEMBL 3763417	3.780	3.711	CHEMBL 105712	4.700	3.642	CHEMBL 2338031	0.900	0.988
CHEMBL 3672411	3.180	3.275	CHEMBL 3764065	3.800	3.960	CHEMBL 2048659	0.602	0.715	CHEMBL 3673159	0.934	0.690
CHEMBL 3703296	3.210	3.332	CHEMBL 3764900	3.820	3.706	CHEMBL 2048683	0.845	1.052	CHEMBL 3673160	0.968	1.113
CHEMBL 3703300	3.260	2.850	CHEMBL 394753	3.870	3.934	CHEMBL 2048673	0.954	0.970	CHEMBL 2333635	1.000	1.127
CHEMBL 3672418	3.300	3.478	CHEMBL 3770866	3.900	3.913	CHEMBL 2048671	1.080	0.313	CHEMBL 3770663	1.040	1.320
CHEMBL 3691383	3.360	3.391	CHEMBL 808	4.140	3.914	CHEMBL 3410087	1.110	0.447	CHEMBL 2333633	1.080	1.443
CHEMBL 3703251	3.370	3.162	CHEMBL 426113	5.220	4.602	CHEMBL 3401120	1.150	1.431	CHEMBL 3703733	1.150	1.520
CHEMBL 3765061	3.430	3.669	CHEMBL 3585776	-1.570	0.215	CHEMBL 2048664	1.230	1.013	CHEMBL 2338048	1.200	0.754
CHEMBL 3672431	3.480	3.394	CHEMBL 3585781	-1.570	-0.633	CHEMBL 2391101	1.280	1.290	CHEMBL 3673139	1.210	1.174
CHEMBL 3672405	3.530	3.346	CHEMBL 3786937	2.120	2.802	CHEMBL 3652550	1.300	1.437	CHEMBL 2407326	1.260	1.356
CHEMBL 3414706	3.710	3.868	CHEMBL 3632988	2.320	2.834	CHEMBL 3410105	1.340	1.887	CHEMBL 2407325	1.320	1.364
CHEMBL	3.810	4.060	CHEMBL	2.360	2.783	CHEMBL	1.430	1.497	CHEMBL	1.370	1.695

50			3410956			3091547			3952992		
CHEMBL 3691379	3.920	3.494	CHEMBL 3632987	2.410	2.827	CHEMBL 3401122	1.480	1.602	CHEMBL 3703749	1.450	0.998
CHEMBL 3752684	4.260	4.493	CHEMBL 3415560	2.480	3.127	CHEMBL 2386105	1.550	0.999	CHEMBL 3323094	1.490	1.243
CHEMBL 3703206	4.780	4.365	CHEMBL 3415565	2.520	2.871	CHEMBL 3401124	1.610	1.549	CHEMBL 2407331	1.510	1.543
CHEMBL 3695744	0.146	0.455	CHEMBL 3827064	2.590	2.840	CHEMBL 3891139	1.660	2.322	CHEMBL 2407327	1.570	1.739
CHEMBL 3695749	0.447	0.455	CHEMBL 3577517	2.630	3.225	CHEMBL 1834326	1.690	1.525	CHEMBL 3323096	1.600	1.053
CHEMBL 3394212	0.778	1.343	CHEMBL 3905695	2.690	3.037	CHEMBL 1834329	1.760	1.366	CHEMBL 3310483	1.670	1.550
CHEMBL 3980437	0.903	1.011	CHEMBL 3582206	2.710	3.327	CHEMBL 3736086	1.850	1.734	CHEMBL 3797621	1.780	1.880
CHEMBL 3905872	0.954	1.176	CHEMBL 3752555	2.740	2.936	CHEMBL 3938281	1.900	1.573	CHEMBL 3323092	1.830	1.412
CHEMBL 3944362	0.987	0.522	CHEMBL 3623561	2.760	3.276	CHEMBL 2391132	1.920	1.615	CHEMBL 1852472	1.910	1.642
CHEMBL 3921481	1.040	1.145	CHEMBL 3410953	2.790	2.812	CHEMBL 2391129	1.950	1.678	CHEMBL 3593395	1.990	2.272
CHEMBL 3673211	1.150	1.434	CHEMBL 3415581	2.840	3.072	CHEMBL 1738953	2.100	1.868	CHEMBL 3612833	2.030	2.679
CHEMBL 3912215	1.160	1.114	CHEMBL 416	2.880	3.279	CHEMBL 3093051	2.110	2.022	CHEMBL 3331505	2.110	1.939
CHEMBL 3394054	1.240	1.648	CHEMBL 3785861	2.930	3.150	CHEMBL 2390958	2.180	1.407	CHEMBL 3593400	2.180	2.803
CHEMBL 3688678	1.340	1.410	CHEMBL 3944652	2.950	3.428	CHEMBL 2391133	2.210	1.238	CHEMBL 3764774	2.300	2.197
CHEMBL 3947671	1.360	1.515	CHEMBL 3827220	2.960	3.089	CHEMBL 3933780	2.260	2.347	CHEMBL 3331468	2.430	2.194
CHEMBL 3688815	1.400	1.731	CHEMBL 3410952	2.980	2.823	CHEMBL 3093047	2.300	2.242	CHEMBL 3799795	2.480	2.571
CHEMBL 3688636	1.440	1.571	CHEMBL 227075	3.000	3.470	CHEMBL 2064537	2.370	2.048	CHEMBL 2407321	2.530	2.630
CHEMBL 3427252	1.480	1.567	CHEMBL 3582231	3.020	3.396	CHEMBL 1940908	2.400	1.893	CHEMBL 3334794	2.600	2.801
CHEMBL	1.530	1.605	CHEMBL	3.060	3.698	CHEMBL	2.480	2.707	CHEMBL	2.700	3.039

3688822			3765697			2022415			3334800		
CHEMBL 3797680	1.560	1.713	CHEMBL 611941	3.100	3.466	CHEMBL 1809122	2.500	1.903	CHEMBL 3799481	2.750	2.629
CHEMBL 3683919	1.560	1.269	CHEMBL 19215	3.130	3.294	CHEMBL 3734911	2.560	2.969	CHEMBL 3126965	2.830	2.964
CHEMBL 3798544	1.600	1.682	CHEMBL 3958208	3.170	3.098	CHEMBL 454662	2.720	3.082	CHEMBL 3673174	0.322	0.469
CHEMBL 3673222	1.670	1.466	CHEMBL 3763820	3.190	3.626	CHEMBL 3736396	2.760	2.899	CHEMBL 3673152	0.531	0.662
CHEMBL 3688824	1.700	2.007	CHEMBL 2163795	3.230	3.715	CHEMBL 3410102	2.790	3.000	CHEMBL 3673154	0.633	0.626
CHEMBL 3688728	1.720	1.743	CHEMBL 524100	3.290	3.100	CHEMBL 3408213	2.840	2.464	CHEMBL 3703728	0.681	0.754
CHEMBL 3427249	1.780	2.024	CHEMBL 2071425	3.300	3.428	CHEMBL 2022417	2.900	2.592	CHEMBL 3673146	0.716	0.500
CHEMBL 3673196	1.810	2.295	CHEMBL 221753	3.350	3.638	CHEMBL 2022419	2.950	2.075	CHEMBL 3673164	0.771	0.777
CHEMBL 3683845	1.860	1.731	CHEMBL 3582210	3.430	3.490	CHEMBL 2024374	3.040	3.042	CHEMBL 3673150	0.806	0.532
CHEMBL 3683917	1.910	1.407	CHEMBL 496	3.480	3.783	CHEMBL 3093036	3.110	2.396	CHEMBL 2333610	0.833	0.946
CHEMBL 3683881	1.930	2.124	CHEMBL 3628056	3.510	3.828	CHEMBL 3787112	3.260	3.269	CHEMBL 3673151	0.857	0.661
CHEMBL 3688654	1.950	2.240	CHEMBL 3818332	3.520	3.292	CHEMBL 2024361	3.300	3.017	CHEMBL 3673138	0.892	0.652
CHEMBL 3634124	1.970	2.189	CHEMBL 3763973	3.550	3.680	CHEMBL 3093038	3.380	2.480	CHEMBL 2333615	0.919	0.934
CHEMBL 3683874	2.000	1.908	CHEMBL 3764679	3.590	3.718	CHEMBL 2313850	3.600	2.814	CHEMBL 2333642	0.954	1.018
CHEMBL 3650826	2.040	2.029	CHEMBL 3628055	3.620	3.901	CHEMBL 218301	3.790	3.327	CHEMBL 3673179	1.000	0.571
CHEMBL 3688729	2.070	1.617	CHEMBL 3822670	3.690	3.397	CHEMBL 3410089	0.041	0.633	CHEMBL 2337605	1.000	1.044
CHEMBL 3688709	2.090	2.131	CHEMBL 3628060	3.760	3.845	CHEMBL 2386106	0.724	0.772	CHEMBL 3681366	1.080	1.237
CHEMBL 3650831	2.110	2.191	CHEMBL 3763832	3.780	3.791	CHEMBL 3647954	0.903	0.484	CHEMBL 2337591	1.100	0.925
CHEMBL	2.140	2.325	CHEMBL	3.810	3.888	CHEMBL	1.000	0.469	CHEMBL	1.180	1.411

3650849			3957099			2048669			3681358		
CHEMBL 3693229	2.160	1.650	CHEMBL 3764488	3.830	3.882	CHEMBL 2048661	1.080	0.564	CHEMBL 1852617	1.200	1.248
CHEMBL 3703233	2.190	2.357	CHEMBL 305660	3.870	3.508	CHEMBL 3652540	1.110	1.546	CHEMBL 3769872	1.260	1.415
CHEMBL 3654216	2.260	2.676	CHEMBL 239400	3.900	3.976	CHEMBL 3647950	1.200	1.021	CHEMBL 2337598	1.300	0.805
CHEMBL 3683898	2.290	2.572	CHEMBL 91	4.270	3.956	CHEMBL 2391102	1.230	1.225	CHEMBL 3331479	1.360	1.232
CHEMBL 3586197	2.360	2.995	CHEMBL 3585778	-1.570	-0.232	CHEMBL 2386102	1.300	1.137	CHEMBL 3703729	1.410	0.854
CHEMBL 3650822	2.390	2.131	CHEMBL 3585783	-1.570	-1.521	CHEMBL 3736035	1.320	1.355	CHEMBL 3703740	1.470	1.123
CHEMBL 3654221	2.410	2.850	CHEMBL 603622	1.650	1.893	CHEMBL 3401121	1.360	1.410	CHEMBL 3323106	1.510	1.582
CHEMBL 3688716	2.480	2.478	CHEMBL 3632990	2.180	2.761	CHEMBL 3647952	1.480	1.107	CHEMBL 3593396	1.560	2.268
CHEMBL 3673191	2.510	2.613	CHEMBL 3586207	2.340	3.033	CHEMBL 1834324	1.490	1.258	CHEMBL 3703750	1.590	2.155
CHEMBL 3673201	2.580	2.275	CHEMBL 3577522	2.400	3.212	CHEMBL 3410088	1.570	0.669	CHEMBL 3331498	1.650	2.193
CHEMBL 3654228	2.590	2.685	CHEMBL 3415568	2.430	2.870	CHEMBL 2048667	1.640	0.814	CHEMBL 3703725	1.730	1.575
CHEMBL 3673227	2.620	3.139	CHEMBL 3819231	2.510	2.657	CHEMBL 1801633	1.670	0.950	CHEMBL 1852516	1.820	1.618
CHEMBL 3654230	2.670	2.882	CHEMBL 3819383	2.530	2.621	CHEMBL 3091546	1.710	1.813	CHEMBL 3310478	1.860	1.485
CHEMBL 3890737	2.710	2.374	CHEMBL 85251	2.610	2.603	CHEMBL 3736223	1.810	2.295	CHEMBL 2430680	1.930	1.737
CHEMBL 3422239	2.780	2.890	CHEMBL 3586583	2.640	3.329	CHEMBL 1834325	1.880	1.418	CHEMBL 3331507	2.000	1.707
CHEMBL 3650768	2.800	2.699	CHEMBL 3582224	2.700	3.381	CHEMBL 2386104	1.920	1.195	CHEMBL 2407330	2.080	1.778
CHEMBL 3747444	2.830	1.578	CHEMBL 3818689	2.720	2.940	CHEMBL 2064518	1.930	2.181	CHEMBL 2347692	2.150	2.523
CHEMBL 3703290	2.870	2.607	CHEMBL 3597056	2.740	3.172	CHEMBL 1809118	2.000	1.883	CHEMBL 2347856	2.230	2.818
CHEMBL	2.910	3.253	CHEMBL	2.770	3.084	CHEMBL	2.100	1.942	CHEMBL	2.310	2.236

3672410			3623553			2391126			1201066		
CHEMBL 3650798	2.940	2.955	CHEMBL 3415580	2.810	3.078	CHEMBL 3699443	2.130	1.037	CHEMBL 3799668	2.450	2.350
CHEMBL 3650790	2.970	2.867	CHEMBL 3818512	2.880	3.043	CHEMBL 1738952	2.200	1.307	CHEMBL 3593391	2.520	2.345
CHEMBL 3764465	3.020	3.137	CHEMBL 3586576	2.900	3.363	CHEMBL 2064519	2.220	1.790	CHEMBL 3334776	2.590	2.982
CHEMBL 3672419	3.040	2.937	CHEMBL 3921061	2.940	3.395	CHEMBL 1834120	2.280	1.402	CHEMBL 3334793	2.650	2.622
CHEMBL 3677228	3.080	3.354	CHEMBL 3577520	2.950	3.220	CHEMBL 3652546	2.300	1.830	CHEMBL 3126972	2.720	2.607
CHEMBL 3650775	3.100	3.096	CHEMBL 3764100	2.970	3.642	CHEMBL 3410099	2.380	2.287	CHEMBL 2347695	2.820	2.733
CHEMBL 3672439	3.150	3.126	CHEMBL 3910830	2.990	3.189	CHEMBL 1834115	2.460	1.973	CHEMBL 2347703	2.990	2.951
CHEMBL 3650813	3.160	3.251	CHEMBL 3586574	3.000	3.380	CHEMBL 3647953	2.490	2.067			
CHEMBL 3703209	3.190	2.969	CHEMBL 3763232	3.030	3.476	CHEMBL 3735529	2.520	1.761			
CHEMBL 3928706	3.220	3.102	CHEMBL 3402718	3.080	3.589	CHEMBL 3093042	2.700	2.564			
CHEMBL 3677227	3.280	3.666	CHEMBL 3628065	3.110	3.492	CHEMBL 3735445	2.730	2.908			
CHEMBL 3672402	3.320	3.168	CHEMBL 3577509	3.130	3.285	CHEMBL 1801635	2.760	2.841			
CHEMBL 3763815	3.360	3.464	CHEMBL 3818230	3.180	3.534	CHEMBL 3735755	2.810	2.987			
CHEMBL 3672433	3.380	3.031	CHEMBL 3818415	3.190	3.439	CHEMBL 3901767	2.880	2.845			
CHEMBL 3677238	3.430	3.538	CHEMBL 3402708	3.260	3.563	CHEMBL 3735497	2.940	2.579			
CHEMBL 3677219	3.490	4.083	CHEMBL 3402713	3.300	3.488	CHEMBL 3673434	2.960	3.009			
CHEMBL 3691365	3.570	3.665	CHEMBL 3415559	3.320	3.137	CHEMBL 3735382	3.080	2.852			
CHEMBL 3688688	3.710	3.456	CHEMBL 3632852	3.400	3.184	CHEMBL 3911190	3.160	2.507			
CHEMBL	3.830	3.539	CHEMBL	3.440	3.362	CHEMBL	3.280	3.328			

3917763			334255			28					
CHEMBL 3691369	4.010	3.833	CHEMBL 145361	3.490	3.397	CHEMBL 3763318	3.320	3.183			
CHEMBL 3586138	4.340	4.134	CHEMBL 3765569	3.510	3.773	CHEMBL 464381	3.410	2.812			
CHEMBL 3695747	0.146	0.540	CHEMBL 3765476	3.530	3.706	CHEMBL 102953	3.700	3.542			
CHEMBL 3985433	0.699	0.542	CHEMBL 3763482	3.570	3.690	CHEMBL 1668411	4.260	3.781			
CHEMBL 3971416	0.799	1.374	CHEMBL 3628057	3.590	3.830						
CHEMBL 3897568	0.903	1.051	CHEMBL 3823464	3.670	3.403						
CHEMBL 3957841	0.954	1.012	CHEMBL 3623565	3.720	3.463						
CHEMBL 3980315	1.000	1.115	CHEMBL 64894	3.770	3.777						
CHEMBL 3896933	1.040	1.128	CHEMBL 1327885	3.790	3.734						
CHEMBL 3896538	1.150	1.316	CHEMBL 3770071	3.810	3.665						
CHEMBL 3973523	1.160	1.394	CHEMBL 240047	3.850	3.871						
CHEMBL 3688652	1.260	1.764	CHEMBL 3764714	3.890	3.627						
CHEMBL 3427250	1.340	1.683	CHEMBL 1801815	4.000	4.013						
CHEMBL 3683920	1.370	1.341	CHEMBL 3628054	4.460	3.853						
CHEMBL 3688812	1.400	1.262									
CHEMBL 3683866	1.440	1.906									
CHEMBL 3804970	1.490	1.215									
CHEMBL 3634126	1.530	1.980									
CHEMBL	1.560	0.887									

3673204											
CHEMBL 3980733	1.570	2.065									
CHEMBL 3688619	1.600	1.903									
CHEMBL 3693230	1.680	1.507									
CHEMBL 3683847	1.700	1.570									
CHEMBL 3683805	1.740	1.567									
CHEMBL 3688617	1.790	1.805									
CHEMBL 3673231	1.810	2.489									
CHEMBL 3673226	1.870	2.647									
CHEMBL 3673203	1.910	1.861									
CHEMBL 3683916	1.940	1.425									
CHEMBL 3688719	1.960	2.258									
CHEMBL 3688695	1.970	1.855									
CHEMBL 3688615	2.000	2.104									
CHEMBL 3427253	2.040	2.355									
CHEMBL 3683789	2.070	1.978									
CHEMBL 3688626	2.090	2.128									
CHEMBL 3693228	2.110	1.671									
CHEMBL 3688724	2.150	1.673									
CHEMBL	2.160	2.263									

3688635												
CHEMBL 3910646	2.200	2.047										
CHEMBL 3688721	2.260	2.009										
CHEMBL 3688707	2.290	2.158										
CHEMBL 3683903	2.380	2.167										
CHEMBL 3984088	2.400	2.227										
CHEMBL 3688717	2.430	2.168										
CHEMBL 3654215	2.480	3.063										
CHEMBL 3688747	2.530	2.635										
CHEMBL 3650833	2.590	2.614										
CHEMBL 3693239	2.600	2.753										
CHEMBL 3752926	2.630	2.535										
CHEMBL 3654218	2.670	2.396										
CHEMBL 3422235	2.710	3.064										
CHEMBL 3900541	2.780	2.471										
CHEMBL 3747445	2.810	1.885										
CHEMBL 3747381	2.860	1.662										
CHEMBL 3650782	2.880	3.048										
CHEMBL 3688668	2.910	2.878										
CHEMBL	2.940	2.825										

3688611											
CHEMBL 3650777	2.970	3.006									
CHEMBL 3703236	3.030	2.877									
CHEMBL 3672442	3.080	2.465									
CHEMBL 3672426	3.080	3.406									
CHEMBL 3672417	3.110	3.122									
CHEMBL 3672404	3.150	3.369									
CHEMBL 3703259	3.170	3.038									
CHEMBL 3672407	3.200	3.384									
CHEMBL 3650770	3.220	3.124									
CHEMBL 3672421	3.280	3.247									
CHEMBL 3688803	3.320	3.359									
CHEMBL 3677222	3.360	3.987									
CHEMBL 3677234	3.380	2.970									
CHEMBL 3688795	3.440	3.551									
CHEMBL 3650767	3.510	3.269									
CHEMBL 3672430	3.600	3.343									
CHEMBL 3691370	3.730	3.693									
CHEMBL 3703215	3.860	3.878									
CHEMBL	4.020	4.217									

3691366											
CHEMBL 3703245	4.390	4.241									
CHEMBL 3695751	-0.222	0.236									
CHEMBL 3639946	0.380	0.540									
CHEMBL 3960603	0.699	1.062									
CHEMBL 3394040	0.869	1.016									
CHEMBL 3892262	0.954	0.664									
CHEMBL 3695738	0.968	0.503									
CHEMBL 3905497	1.030	1.341									
CHEMBL 3891061	1.080	0.923									
CHEMBL 3394045	1.160	1.546									
CHEMBL 3688660	1.210	1.504									
CHEMBL 3683911	1.280	1.423									
CHEMBL 3394228	1.360	1.496									
CHEMBL 3695734	1.390	1.155									
CHEMBL 3394215	1.410	1.673									
CHEMBL 3688738	1.460	1.503									
CHEMBL 3683843	1.510	1.544									
CHEMBL 3639876	1.550	1.904									
CHEMBL	1.560	1.572									

3683889											
CHEMBL 3688813	1.590	1.508									
CHEMBL 3688656	1.670	2.008									
CHEMBL 3746637	1.700	2.135									
CHEMBL 3798199	1.710	2.127									
CHEMBL 3640259	1.760	2.226									
CHEMBL 3683811	1.800	1.774									
CHEMBL 3427247	1.850	2.323									
CHEMBL 3683876	1.910	1.926									
CHEMBL 3683873	1.920	1.996									
CHEMBL 3933659	1.950	2.320									
CHEMBL 3688612	1.960	1.999									
CHEMBL 3683855	1.990	1.806									
CHEMBL 3688616	2.030	2.162									
CHEMBL 3693233	2.060	1.895									
CHEMBL 3747766	2.080	2.073									
CHEMBL 3688663	2.100	2.673									
CHEMBL 3683801	2.130	1.997									
CHEMBL 3703285	2.160	1.658									
CHEMBL	2.180	2.596									

3960008											
CHEMBL 3654232	2.210	2.717									
CHEMBL 3683882	2.270	1.774									
CHEMBL 3986498	2.360	2.157									
CHEMBL 3688752	2.380	2.886									
CHEMBL 3693257	2.400	2.381									
CHEMBL 3688662	2.450	1.996									
CHEMBL 3654223	2.500	2.833									
CHEMBL 3650825	2.540	2.549									
CHEMBL 3650772	2.590	2.994									
CHEMBL 3650774	2.600	3.054									
CHEMBL 3654226	2.640	2.992									
CHEMBL 3677236	2.680	2.914									
CHEMBL 3688691	2.720	2.660									
CHEMBL 3677231	2.790	2.601									
CHEMBL 3745922	2.820	3.166									
CHEMBL 3765711	2.860	3.091									
CHEMBL 3672413	2.900	2.720									
CHEMBL 3703272	2.920	2.839									
CHEMBL	2.960	3.278									

3691362												
CHEMBL 3650807	3.000	2.742										
CHEMBL 3650795	3.030	2.898										
CHEMBL 3672408	3.080	3.111										
CHEMBL 3691376	3.090	3.335										
CHEMBL 3752467	3.130	3.120										
CHEMBL 3672440	3.150	3.146										
CHEMBL 3677220	3.180	3.343										
CHEMBL 3672415	3.200	3.583										
CHEMBL 3677244	3.230	3.122										
CHEMBL 3672443	3.300	3.597										
CHEMBL 3672416	3.340	3.346										
CHEMBL 3650757	3.360	3.286										
CHEMBL 3703294	3.420	3.487										
CHEMBL 3703241	3.460	3.607										
CHEMBL 3677229	3.530	2.992										
CHEMBL 3672717	3.690	3.945										
CHEMBL 3765319	3.780	3.536										
CHEMBL 3691364	3.900	3.940										
CHEMBL	4.240	4.148										

3414705											
CHEMBL 3703222	4.480	4.286									

Table S1. The results of the virtual screening with VSW module of the Scrödinger suite.

ZINC code	MW[1]	HBD[2]	HBA[3]	NROT[4]	logP[5]	Targets							
						AChE		BACE1		GSK3β		SERT	
						Binding energy, kcal/mol	LE	Binding energy, kcal/mol	LE	Binding energy, kcal/mol	LE	Binding energy, kcal/mol	LE
ZINC323331	308.3	3	3	2	2.562	-	-	-	-	-12.82	0.56	-9.12	0.40
ZINC49169727	268.3	2	3	3	0.949	-11.07	0.55	-6.76	0.34	-	-	-	-

Table S2. The results of the virtual screening with AutoDock Vina 1.1.2.

ZINC code	MW	HBD	HBA	NROT	logP	Solubility category[6]	Targets							
							AChE		BACE1		GSK3β		SERT	
							Binding energy, kcal/mol	LE	Binding energy, kcal/mol	LE	Binding energy, kcal/mol	LE	Binding energy, kcal/mol	LE
ZINC1219	268.2	2	3	0	2.397	Moderate	-9.7	0.49	-10.0	0.50	-9.1	0.46	-9.8	0.49
ZINC116738	273.3	1	3	2	1.234	High	-8.2	0.41	-9.3	0.47	-8.4	0.42	-8.7	0.44
ZINC1034491	279.3	1	3	1	2.778	Moderate	-9.8	0.47	-10.0	0.48	-8.7	0.41	-10.3	0.49
ZINC1763229	270.2	2	3	1	1.177	High	-9.2	0.46	-8.9	0.45	-8.0	0.40	-8.9	0.44
ZINC2129833	301.4	1	3	2	2.112	High	-9.8	0.45	-9.7	0.44	-8.9	0.40	-9.1	0.41
ZINC2132617	292.4	1	1	0	2.702	Moderate	-9.2	0.42	-10.5	0.48	-9.5	0.43	-10.4	0.47

[1] MW – molecular weight;

[2] HBD – the number of the hydrogen bonds donors;

[3] HBA – the number of the hydrogen bonds acceptors;

[4] NROT – the number of rotatable bonds;

[5] logP - logarithm of the octanol-water partition coefficient;

[6] low: if solubility is < 0.01 mg/ml; moderate: if solubility is between 0.01 and 0.06 mg/ml, high: if solubility is > 0.06 mg/ml;

ZINC code	MW	HBD	HBA	NROT	logP	Solubility category[6]	Targets							
							AChE		BACE1		GSK3β		SERT	
							Binding energy, kcal/mol	LE	Binding energy, kcal/mol	LE	Binding energy, kcal/mol	LE	Binding energy, kcal/mol	LE
ZINC3805477	284.3	3	3	1	0.533	Moderate	-8.9	0.42	-9.6	0.46	-8.4	0.40	-10.1	0.48
ZINC3814414	290.4	2	2	0	2.7987	High	-9.5	0.45	-10.4	0.49	-8.4	0.40	-8.8	0.42
ZINC3977996	288.4	3	3	0	2.671	High	-9.2	0.44	-10.2	0.49	-9.1	0.43	-10.1	0.48
ZINC4028010	273.3	1	3	3	1.486	Moderate	-9.6	0.48	-9.6	0.48	-8.7	0.44	-9.6	0.48
ZINC4028387	271.3	1	3	0	1.348	High	-8.4	0.42	-9.8	0.49	-8.1	0.41	-9.5	0.48
ZINC4041614	269.3	1	2	0	0.232	High	-9.1	0.46	-9.8	0.49	-8.8	0.44	-9.7	0.49
ZINC4082030	274.4	1	2	0	2.662	High	-9.3	0.47	-10.3	0.52	-8.5	0.43	-8.3	0.42
ZINC8623851	268.4	1	2	2	2.981	High	-8.2	0.41	-8.8	0.44	-8.0	0.40	-8.3	0.42
ZINC14684865	270.3	1	3	2	2.212	High	-8.5	0.43	-9.3	0.47	-8.8	0.44	-9.0	0.45
ZINC35446833	266.3	1	3	2	2.347	High	-8.8	0.44	-8.9	0.45	-8.0	0.40	-9.4	0.47
ZINC49169727	268.3	2	3	3	0.949	High	-10.0	0.50	-9.1	0.46	-8.1	0.41	-8.9	0.45
ZINC49170581	275.3	0	3	3	2.217	High	-9.9	0.50	-8.7	0.44	-8.0	0.40	-9.3	0.47
ZINC49170716	275.3	0	3	2	0.623	High	-9.9	0.50	-8.9	0.45	-8.3	0.42	-9.2	0.46
ZINC49170953	271.3	0	3	2	1.041	High	-10.3	0.52	-9.8	0.49	-8.3	0.42	-9.5	0.48
ZINC225482257	272.3	0	3	2	2.193	High	-8.7	0.44	-8.0	0.40	-8.5	0.43	-8.8	0.44
ZINC35466008	280.4	1	3	1	2.536	High	-8.1	0.41	-8.8	0.44	-8.1	0.41	-8.4	0.42
ZINC286766	273.3	0	3	1	3.095	High	-8.6	0.43	-8.9	0.45	-8.3	0.42	-9.2	0.46
ZINC338038	270.3	1	4	2	2.635	High	-9.8	0.49	-9.1	0.46	-8.3	0.42	-9.7	0.49
ZINC391977	270.2	3	5	1	2.427	High	-9.4	0.47	-8.5	0.43	-8.3	0.42	-9.7	0.49
ZINC402720	314.3	0	5	1	1.572	High	-9.3	0.40	-10.4	0.45	-9.4	0.41	-9.5	0.41
ZINC518541	262.4	1	1	2	3.469	Moderate	-9.5	0.48	-9.6	0.48	-8.4	0.42	-10.2	0.51
ZINC519113	309.4	0	3	0	3.385	Moderate	-9.7	0.42	-10.8	0.47	-9.3	0.40	-9.7	0.42
ZINC525976	284.4	1	2	1	3.214	High	-9.9	0.47	-10.0	0.48	-8.7	0.41	-8.4	0.40
ZINC565682	285.4	2	3	1	3.363	High	-9.3	0.47	-9.6	0.48	-8.5	0.43	-9.1	0.46
ZINC898768	272.3	3	5	1	2.185	High	-10.4	0.52	-9.1	0.46	-8.3	0.42	-9.9	0.50
ZINC1609684	291.3	1	4	1	2.133	Moderate	-10.2	0.46	-10.2	0.46	-8.9	0.40	-9.6	0.44
ZINC1801081	274.3	0	3	4	1.925	Moderate	-9.1	0.46	-9.6	0.48	-8.1	0.41	-8.8	0.44
ZINC2131836	275.3	2	5	2	0.977	High	-8.3	0.42	-9.3	0.47	-8.0	0.40	-8.5	0.43
ZINC3814360	290.4	1	2	0	3.411	Moderate	-10.2	0.49	-10.7	0.51	-8.8	0.42	-9.1	0.43
ZINC4023561	284.3	1	4	0	2.15	High	-9.6	0.46	-10.4	0.50	-9.2	0.44	-9.3	0.44
ZINC4023795	292.4	2	2	0	3.204	Moderate	-9.2	0.44	-10.1	0.48	-8.5	0.41	-8.6	0.41
ZINC4025911	314.4	0	4	0	0.953	Moderate	-10.3	0.47	-11.0	0.50	-9.3	0.42	-9.5	0.43
ZINC4027357	278.4	1	1	0	3.476	Moderate	-9.3	0.44	-10.0	0.48	-8.6	0.41	-10.3	0.49

ZINC code	MW	HBD	HBA	NROT	logP	Solubility category[6]	Targets							
							AChE		BACE1		GSK3β		SERT	
							Binding energy, kcal/mol	LE	Binding energy, kcal/mol	LE	Binding energy, kcal/mol	LE	Binding energy, kcal/mol	LE
ZINC4028576	313.4	0	4	1	2.042	High	-9.4	0.41	-10.0	0.43	-9.3	0.40	-10.7	0.47
ZINC4038093	275.3	2	4	1	-0.238	High	-8.0	0.40	-9.2	0.46	-8.0	0.40	-8.2	0.41
ZINC4040762	292.3	0	2	1	3.145	Moderate	-9.9	0.45	-10.0	0.45	-9.1	0.41	-9.8	0.45
ZINC4090104	285.3	2	4	3	1.819	Moderate	-9.8	0.47	-9.6	0.46	-8.5	0.40	-9.7	0.46
ZINC4538008	288.4	1	2	0	3.365	Moderate	-9.5	0.45	-10.7	0.51	-9.1	0.43	-8.7	0.41
ZINC4654724	322.3	2	5	0	2.623	Moderate	-10.5	0.44	-11.1	0.46	-9.9	0.41	-9.6	0.40
ZINC4655109	272.3	3	5	1	2.728	High	-9.9	0.50	-9.0	0.45	-8.2	0.41	-9.5	0.48
ZINC5179869	271.4	0	2	2	3.319	Moderate	-9.4	0.47	-9.7	0.49	-8.1	0.41	-9.5	0.48
ZINC5733557	268.3	1	4	2	2.506	Moderate	-9.6	0.48	-9.2	0.46	-8.2	0.41	-9.6	0.48
ZINC6525252	270.2	3	5	1	2.427	High	-9.4	0.47	-9.1	0.46	-8.1	0.41	-9.1	0.46
ZINC14719962	312.3	2	4	0	1.919	Moderate	-10.0	0.43	-10.4	0.45	-9.2	0.40	-10.0	0.43
ZINC14829817	276.3	2	5	1	2.167	High	-9.5	0.48	-9.0	0.45	-8.3	0.42	-8.3	0.42
ZINC31158814	310.3	3	4	0	2.68114	Moderate	-11.1	0.48	-11.0	0.48	-9.5	0.41	-9.4	0.41
ZINC35446839	294.4	1	3	2	3.079	High	-9.1	0.41	-9.7	0.44	-9.0	0.41	-10.1	0.46
ZINC38304118	288.3	3	4	0	2.996	Moderate	-9.7	0.46	-10.5	0.50	-9.2	0.44	-8.9	0.42
ZINC100074489	266.3	1	4	1	1.078	High	-9.8	0.49	-9.5	0.48	-8.3	0.42	-9.1	0.46
ZINC225417079	272.3	2	3	0	3.42	High	-9.0	0.45	-10.1	0.51	-9.1	0.46	-9.2	0.46
ZINC252463663	280.3	2	4	1	-0.015	High	-8.3	0.42	-8.6	0.43	-8.3	0.42	-8.1	0.41

Table S3. Predicted LogIC50 values of selected compounds for ANN and BMLR models

ZINC code	Targets																Average $logIC_{50}$	AD
	AChE				BACE1				GSK3β				SERT					
	MLR model	AD	ANN model	AD	MLR model	AD	ANN model	AD	MLR model	AD	ANN model	AD	MLR model	AD	ANN model	AD		
ZINC1219	2.456	1	3.690	1	3.740	1	3.958	1	2.652	1	3.160	1	0.376	0	1.715	0	2.718	0
ZINC116738	2.949	1	3.789	1	3.732	1	3.592	1	2.964	1	2.938	1	1.589	1	0.865	1	2.802	1
ZINC286766	3.333	1	3.896	1	3.563	1	3.429	1	4.334	1	3.142	1	0.918	1	0.617	1	2.904	0
ZINC338038	2.814	1	3.767	1	3.997	1	3.779	1	4.141	1	3.193	1	2.538	1	1.118	1	3.168	1
ZINC391977	2.468	1	3.676	1	3.870	1	3.897	1	3.274	1	3.158	1	0.776	0	1.692	0	2.851	0
ZINC402720	2.563	1	3.691	1	3.147	1	3.381	1	2.328	1	2.514	1	-0.770	0	0.567	0	2.178	0
ZINC518541	2.739	1	3.658	1	4.529	1	3.766	1	4.588	0	3.160	1	1.305	1	0.783	1	3.066	0
ZINC519113	3.430	1	3.938	1	3.724	1	3.412	1	4.983	1	3.542	1	0.470	1	0.610	1	3.014	1
ZINC525976	3.676	1	4.007	1	3.064	1	3.234	1	4.244	0	3.338	1	1.676	1	0.619	1	2.982	0
ZINC565682	3.354	1	3.870	1	3.923	1	3.645	1	3.150	1	2.677	1	3.001	0	0.800	0	3.053	0
ZINC898768	2.587	1	3.699	1	4.026	1	3.844	1	3.949	1	3.254	1	3.799	0	1.601	0	3.345	0
ZINC1034491	2.715	1	3.665	1	4.114	1	3.776	1	2.637	1	2.684	1	1.107	0	1.044	0	2.718	0
ZINC1609684	3.218	1	3.934	1	2.452	0	3.565	1	1.548	1	2.385	1	-0.084	0	1.164	0	2.273	0
ZINC1763229	2.729	1	3.659	1	3.499	1	3.631	1	3.795	1	2.997	1	2.768	0	0.990	0	3.008	0
ZINC1801081	2.544	1	3.675	1	4.028	1	3.739	1	2.933	1	2.636	1	0.273	0	0.759	0	2.573	0
ZINC2129833	2.952	1	3.782	1	3.820	1	3.453	1	3.043	1	2.997	1	1.885	1	0.884	1	2.852	1
ZINC2131836	2.847	1	3.763	1	3.446	1	3.551	1	4.136	1	3.320	1	1.002	0	0.843	0	2.863	0
ZINC2132617	3.270	1	3.819	1	3.135	1	3.239	1	4.868	1	3.396	1	0.834	0	0.600	0	2.895	0
ZINC3805477	2.822	1	3.657	1	4.001	1	3.593	1	4.308	0	3.312	1	2.187	1	0.765	1	3.081	0
ZINC3814360	3.115	1	3.805	1	4.736	1	3.688	1	6.247	0	3.805	1	2.023	0	1.180	0	3.575	0
ZINC3814414	3.223	1	3.841	1	4.205	1	3.511	1	6.281	0	3.714	1	1.478	0	0.844	0	3.387	0
ZINC3977996	2.931	1	3.763	1	3.637	1	3.380	1	5.046	0	3.403	1	1.575	0	0.778	0	3.064	0
ZINC4023561	3.335	1	3.934	1	3.392	1	3.528	1	3.467	1	2.665	1	2.645	0	0.893	0	2.982	0
ZINC4023795	3.144	1	3.809	1	5.280	0	3.830	1	9.760	0	4.166	1	1.935	0	1.123	0	4.131	0
ZINC4025911	3.194	1	3.908	1	3.641	1	3.554	1	2.272	1	2.745	1	0.667	0	0.607	0	2.573	0
ZINC4027357	2.824	1	3.677	1	3.518	1	3.356	1	4.683	1	3.291	1	0.763	0	0.577	0	2.836	0
ZINC4028010	2.207	1	3.467	1	3.661	1	3.625	1	2.366	1	2.647	1	1.628	0	1.075	0	2.585	0
ZINC4028387	3.167	1	3.866	1	3.625	1	3.572	1	3.856	1	3.151	1	2.574	0	0.921	0	3.092	0
ZINC4028576	2.947	1	3.798	1	3.186	1	3.265	1	3.578	1	3.097	1	1.270	1	0.579	1	2.715	1
ZINC4038093	2.771	1	3.737	1	3.270	1	3.520	1	3.393	1	3.132	1	1.130	0	0.804	0	2.720	0
ZINC4040762	3.631	1	4.026	1	3.615	1	3.534	1	4.766	1	3.294	1	0.969	1	0.707	1	3.068	0

ZINC code	Targets																Average $\log IC_{50}$	AD
	AChE				BACE1				GSK3β				SERT					
	MLR model	AD	ANN model	AD	MLR model	AD	ANN model	AD	MLR model	AD	ANN model	AD	MLR model	AD	ANN model	AD		
ZINC4041614	3.223	1	3.819	1	3.725	1	3.643	1	4.402	1	3.137	1	2.137	0	0.912	0	3.125	0
ZINC4082030	3.151	1	3.825	1	4.196	1	3.536	1	6.116	0	3.854	1	2.036	0	1.078	0	3.474	0
ZINC4090104	1.975	1	3.407	1	3.577	1	3.722	1	2.817	1	2.828	1	2.234	0	1.365	0	2.741	0
ZINC4538008	3.217	1	3.845	1	4.155	1	3.516	1	4.641	1	3.572	1	1.887	0	0.966	0	3.225	0
ZINC4654724	2.888	1	3.799	1	2.715	1	3.315	1	2.630	1	3.219	1	1.898	0	1.036	0	2.688	0
ZINC4655109	2.549	1	3.686	1	4.154	1	3.873	1	3.846	1	3.250	1	3.224	0	1.478	0	3.258	0
ZINC5179869	3.147	1	3.847	1	4.136	1	3.627	1	2.934	1	2.978	1	1.539	1	0.772	1	2.872	0
ZINC5733557	2.804	1	3.777	1	3.977	1	3.840	1	3.422	1	3.154	1	1.597	0	1.307	0	2.985	0
ZINC6525252	2.453	1	3.671	1	3.938	1	3.910	1	3.505	1	3.297	1	0.912	0	1.737	0	2.928	0
ZINC8623851	3.882	1	4.082	1	3.613	1	3.528	1	3.849	1	3.283	1	1.060	1	0.743	1	3.005	0
ZINC14684865	2.961	1	3.714	1	3.122	1	3.462	1	4.134	1	3.360	1	1.696	0	0.868	0	2.915	0
ZINC14719962	2.491	1	3.663	1	2.570	1	3.220	1	2.064	1	2.886	1	1.445	0	0.792	0	2.391	0
ZINC14829817	2.429	1	3.628	1	3.659	1	3.607	1	3.757	1	3.185	1	1.426	0	0.970	0	2.833	0
ZINC31158814	2.957	1	3.800	1	2.592	1	3.149	1	3.233	1	3.212	1	1.858	1	0.743	1	2.693	1
ZINC35446833	2.855	1	3.704	1	4.256	1	3.685	1	3.532	1	3.182	1	1.517	1	0.952	1	2.960	1
ZINC35446839	2.659	1	3.627	1	4.305	1	3.569	1	3.770	1	3.285	1	2.642	1	0.916	1	3.097	1
ZINC35466008	2.837	1	3.729	1	3.795	0	3.597	0	3.723	1	3.130	1	2.532	0	1.085	0	3.054	0
ZINC38304118	2.866	1	3.757	1	3.453	0	3.553	0	3.271	1	3.168	1	1.250	0	0.930	0	2.781	0
ZINC49169727	2.988	1	3.739	1	4.041	1	3.659	1	3.434	1	2.758	1	1.421	1	0.846	1	2.861	0
ZINC49170581	3.221	1	3.893	1	4.213	1	3.721	1	2.841	1	2.857	1	1.673	1	0.728	1	2.893	1
ZINC49170716	3.106	1	3.873	1	4.237	1	3.819	1	3.622	1	2.903	1	0.681	1	0.758	1	2.875	1
ZINC49170953	3.271	1	3.911	1	4.286	1	3.740	1	3.021	1	2.995	1	0.641	1	0.738	1	2.825	1
ZINC100074489	3.307	1	3.956	1	2.699	1	3.655	1	1.751	1	2.553	1	0.469	0	0.889	0	2.410	0
ZINC225417079	3.024	1	3.805	1	2.978	1	3.373	1	3.514	1	3.360	1	2.005	0	0.814	0	2.859	0
ZINC225482257	3.478	1	3.958	1	4.128	1	3.623	1	4.377	0	3.114	1	1.122	1	0.719	1	3.065	0
ZINC252463663	2.643	1	3.678	1	3.382	1	3.524	1	3.843	1	3.083	1	2.740	0	1.122	0	3.002	0

Table S4. The analysis of the interactions between receptors and small molecule ligands

ZINC code	Structure	AChE	BACE1	GSK3β	SERT
ZINC1219	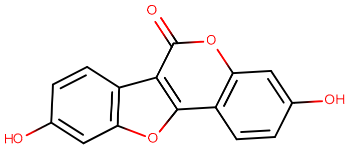	Asp74, Trp86, Tyr124, Ser125, Glu202, Tyr337, Tyr341, Gly448	Tyr71, Phe108	Ala83, Lys85, Leu132, *Val135 (NH…OH)*, Leu188, Cys199, Asp200	Ile172, Tyr176, Gly338, Phe341, Ser439
ZINC116738	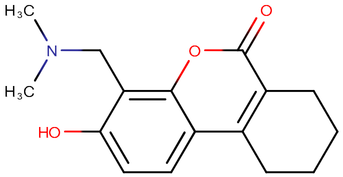	Trp86, Asn87, Gly121, Tyr124, Ser125, Tyr337, Tyr341	Tyr71, Thr72, Phe108, Ile118	Ile62, Val70, Ala83, *Lys85 (NH_3^+…O)*, Tyr134, Val135, Leu188	Gly100, Gln332, *Phe335 (O…H_2N)*, Gly498, Phe556
ZINC286766	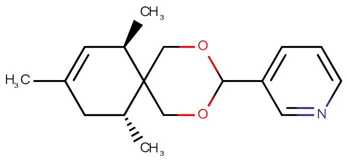	*Tyr72 (O…HN)*, Val73, Asp74, Leu76, Tyr124, Trp286, Tyr341	Tyr71, Phe108, Ile118	Val70, Ala83, Val135, Leu188, Cys199	Tyr95, Ile172, Ser438, Gly442
ZINC338038	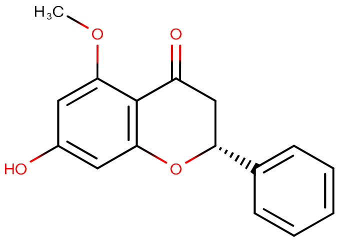	Asp74, Trp86, Gly121, Tyr124, Ser125, Tyr337, Tyr341	Tyr71, Phe108, Ile118, Thr231, *Arg235 (NH_2…OH)*	*Asn64 (NH…OH, O…HO)*, Val70, Ala83, Lys85, Leu132, Asp133, Val135, Leu188, Cys199	Tyr95, Ile172, Tyr176, Gly338, Phe341, Ser438, Ser439, Gly442

ZINC code	Structure	AChE	BACE1	GSK3β	SERT
ZINC391977	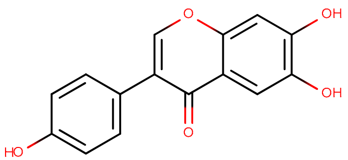	Asp74, Trp86, Tyr124, Trp286, Tyr341	Tyr71, Lys224	Ile62, *Lys85 (NH$_3^+$…OH)*, Leu132, Thr138, Arg141, Leu188, Cys199, Asp200	Tyr95, Ile172, Ala173, Tyr176, Asn177, Phe335, Ser336, Ser438, Ser439, Gly442
ZINC402720	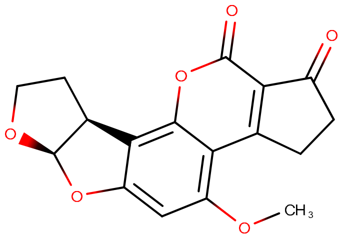	Asp74, *Trp86 (O…HO)*, Gly121, Tyr124, Ser125, Gly126, Tyr337, Tyr347, His447, Gly448	Ser35, Tyr71, Gln73, Phe108, Ile118	*Val135 (NH…O)*, Arg141, Leu188, Cys199	Arg104, *Tyr175 (OH…O)*, Phe335, *Thr497 (OH…O)*, Phe556
ZINC518541	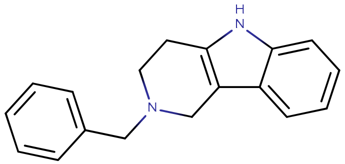	Asp74, Tyr124, Ser125, Trp286, Tyr337, Phe338, Tyr341	Tyr71, Phe108, Lys224, Ile226	Ile62, Val70, Ala83, Lys85, Leu188	Ile172, Phe341, Ser438, Gly442, Val501
ZINC519113	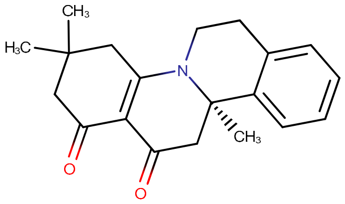	Tyr72, Asp74, Leu76, Tyr124, Trp286, Tyr341	Tyr71, Thr72, Tyr198, Ile226, Thr329	Gly63, *Asn64 (NH…O)*, Val70, Ala83, Thr138, Leu188, Cys199	Ile172, Tyr176, Gly338, Phe341, Ser439, Gly442

ZINC code	Structure	AChE	BACE1	GSK3β	SERT
ZINC525976	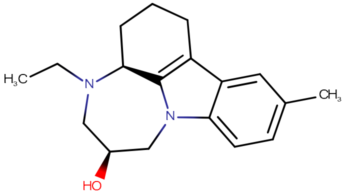	Asp74, Tyr124, Trp286, Leu289, Tyr341	Gly34, Tyr71, Phe108, Val332	Ile62, Ala83, Lys85, Leu132, Vla135, Thr138, Gln185, Leu188, Cys199, Asp200	Tyr95, Ile172, Phe335, Gly442
ZINC565682	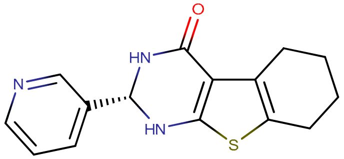	Tyr72, Asp74, Trp86, Asn87, Tyr124, Trp286, Phe338, Tyr341	Tyr71, Phe108, Ile226	Ile62, Leu132, Val135, Leu188	Ile172, Ala173, Tyr176, Asn177, *Phe335 (O…HN)*, Gly338, Phe341, Ser439
ZINC898768	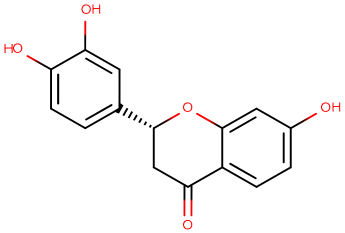	Val73, Asp74, Trp86, Asn87, Tyr124, Trp286, Phe338, Tyr241	Ser35, Tyr71, Ile118, Arg235, *Thr329 (OH…HO)*	Lys85, Leu132, Arg141, Leu188	Tyr95, Ala96, Ile172, Tyr176, Phe341, Ser439, Gly442
ZINC1034491	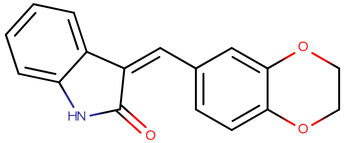	Asp74, Trp86, Gly121, *Tyr124 (HO…HN)*, *Ser125 (OH…O)*, Glu202, Tyr337, Tyr347, His447	Tyr71, Phe108, Ile226, *Asp228 (O…HN)*, Thr231, Val332	Ile62, Val70, *Lys85 (NH_3^+…O)*, Val135, Lys183 *(NH_3^+…O)*, Leu188, Asp200	Ile172, *Tyr175 (OH…O)*, Asn177, *Phe335 (O…HN)*, Gly338, Phe341, Ser439

ZINC code	Structure	AChE	BACE1	GSK3β	SERT
ZINC1609684	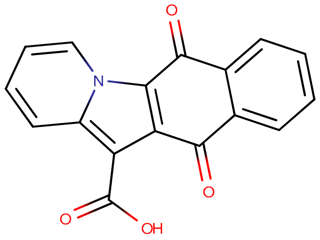	Asp74, Trp86, Gly121, Tyr124, Ser125, Gly126, *Tyr337 (OH…O)*, Tyr341	Tyr71, Thr72, Phe108, Tyr198	Ala83, Lys85, Leu132, Val135, Leu188	Arg104, Tyr176, Phe335, Thr497, Gly498, Phe556
ZINC1763229	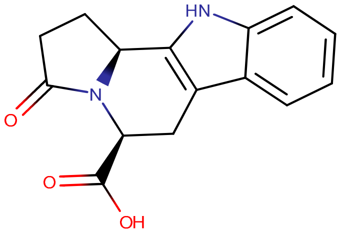	Tyr124, Trp286, Phe338	Tyr71, Phe108	Ala83, Tyr134, Val135, Thr138	Tyr95, Ile172, Phe341, Ser438
ZINC1801081	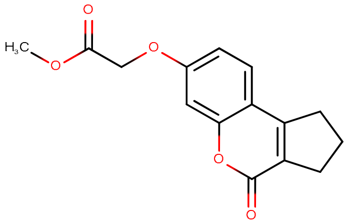	Asp74, Thr75, Trp86, Gly121, Tyr124, *Ser125 (HO…O)*, Tyr341	*Ser35 (OH…O)*, Asn37, *Tyr71 (OH…O)*, Gln73, *Trp76 (NH…O)*, Ile118	Asn64, Gly68, Val70, Ala83, *Lys85 (NH_3^+…OH)*, Leu132, Val135, Leu188, Asp200	Ile172, Tyr176, *Asn177 (NH2…O)*, Phe341, Ser439, Gly442
ZINC2129833	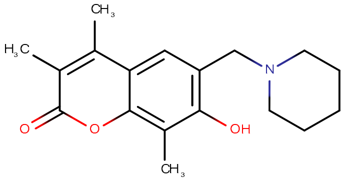	Asp74, *Thr75 (NH…O)*, Tyr124, Trp286, Phe295, Phe338, Tyr341	Ser35, Tyr71, Gln73, Phe108, Ile110	Asn64, Gly65, Val70, *Lys85 (NH_3^+…O)*, Leu132, Val135, Asp200	Ile172, Tyr175, Tyr176, Phe335, Gly442, Glu493, Thr497

ZINC code	Structure	AChE	BACE1	GSK3β	SERT
ZINC2131836	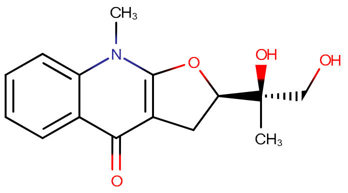	Asp74, Trp86, Gly121, Gly1222, Ser125, Ser203, *Tyr*337, Tyr341, His447, Gly448	*Gly34 (O…HO),* Tyr71, Phe108, Thr231	Ile62, Lys85, Arg141, Leu188	Gly100, Trp103, Arg104, Phe335, Thr497
ZINC2132617	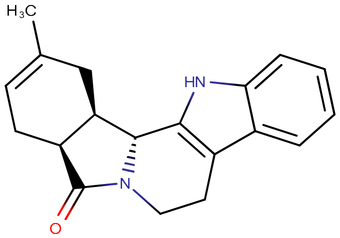	Trp286, Val294, Tyr341	Tyr71, Tyr198	Val70, Ala83, Leu132, Tyr134, Leu188, Cys199	Ile172, Phe341, Gly442
ZINC3805477	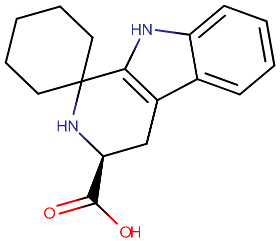	Trp286, Val294, Phe297, Phe338, Tyr341	Tyr71, Phe108, Ile118	Ile62, Gly63, Val70, Ala83, Leu132, Val135, Leu188, Cys199	Ile172, Tyr176, Phe341, Gly442, Val501
ZINC3814360	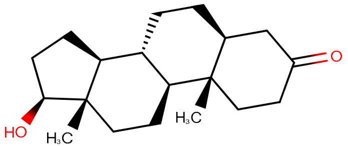	Tyr72, Trp286, *Ser341 (NH…OH),* Tyr341	Tyr71, Gln73, Phe108	Ile62, Val70, Ala83, Lys85, Val135, Lys183, Leu188, Cys199	Tyr95, *Asp98 (COO-…HO),* Ile172, Tyr176, Gly338, Phe341, Ser438, Ser439

ZINC code	Structure	AChE	BACE1	GSK3β	SERT
ZINC3814414	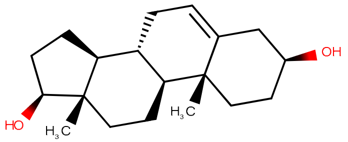	Tyr72, Asp74, *Thr75 (NH…OH)*, Trp286, Leu289, *Ser293 (NH. OH)*, Tyr341	Tyr71, Gln73, Phe108	Ile62, Val70, Ala83, Lys85, Val135, Leu188, Cys199, Asp200	Tyr95, Ala169, Ile172, Ala173, Tyr176, *Phe335 (O…HO)*, Phe341, Ser439, Gly442
ZINC3977996	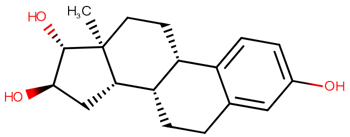	*Asp74 (O…HO)*, Thr75, Leu76, *Trp286 (O…HO)*, *Ser293 (NH…OH)*	Asp32, Tyr71, *Phe108 (O…HO)*, Asp228	Ile62, Val70, *Lys85 ($N^{+}H_3$…OH)*, Leu132, Val135, *Pro136 (O…HO)*, Leu188, Cys199, *Asp200 (NH…O)*	Tyr95, Ala169, Ile172, Ala173, Gly338, Phe341, *Ser439 (O…HO, HO…HO)*, Gly442, Leu443
ZINC4023561	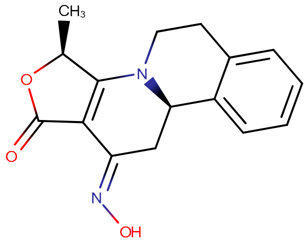	Asp74, Trp86, Gly120, Gly121, Tyr124, Ser125, Tyr337	Gly34, Tyr71, Thr72, Phe108, *Tyr198 (OH…O)*	Ile62, Gly63, Asn64, Ala83, Lys85, Tyr134, Val135, Leu188, Cys199	Ile172, *Tyr175 (OH…N)*, Tyr176, Phe335, *Thr497 (OH…O)*, Val501
ZINC4023795	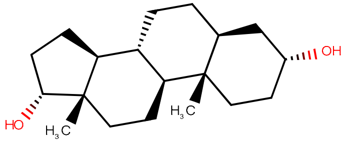	Tyr72, Leu76, Trp286, Leu289, Tyr341	Tyr71, *Gln73 (O…HO)*, Phe108	Ile62, Val135, *Thr138 (OH…OH)*, Leu188	Tyr95, Ile172, Tyr175, Tyr176, Phe335, Phe341, Glu493, *Thr497 (OH…OH)*

ZINC code	Structure	AChE	BACE1	GSK3β	SERT
ZINC4025911	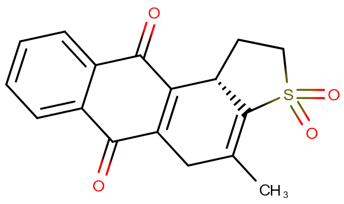	Trp286, Tyr341	Tyr71, Phe108, Arg235, Thr329	*Asn64 (NH…O$_2$S)*, Ala83, Leu132, Asp133, Val135, Leu188	Tyr95, Ile172, Ala173, Phe341, Ser438, Ser439, Gly442, Leu443
ZINC4027357	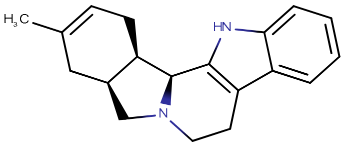	Tyr72, *Asp74 (COO$^-$ …HN$^+$)*, Trp86, Tyr124, Ser125, Trp286, Tyr337, *Tyr341(HO…HN)*	Leu30, *Asp32 (COO- …HN$^+$)*, Tyr71, Phe108, Ile110	Ile62, *Gly63 (O…HN)*, Val70, Val135, Thr138, Tyr140, Arg141, Gln185, Leu188	*Tyr95 (HO…HN, HN$^+$)*, Ile172, Tyr176, Phe335, Ser438, Gly442
ZINC4028010	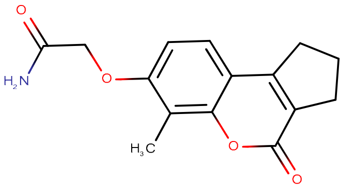	Tyr72, Trp86, Gly121, Ser125, Tyr337, Tyr341	Tyr71, Phe108, Asp228, Thr231, Arg235	Ile62, Val70, Lys85, Val135, Leu188, Asp200	Tyr95, Ala96, Ala169, Ile172, Ala173, Tyr176, Asn177, Leu337, Gly338, Phe341, Ser439, Gly442
ZINC4028387	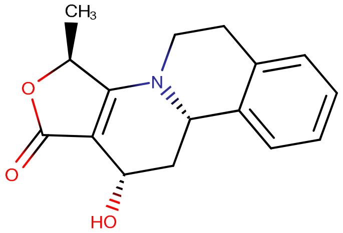	Tyr72, Thr75, Tyr124, Trp286, Phe338, Tyr341	Tyr71	Ile62, Gly63, Val70, Ala83, Val135, Thr138, Leu188	Tyr95, Asp98, Ile172, Tyr176, Phe335, Phe341, Ser438, Ser439, Gly442

ZINC code	Structure	AChE	BACE1	GSK3β	SERT
ZINC4028576	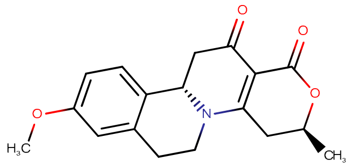	Tyr72, Asp74, Trp86, Tyr124, Ser125, Tyr341	Tyr71, Lys224, Arg235, Thr329	Ile62, Val70, Lys85, *Val135 (NH…O)*, Thr138, Leu188, Cys199	Tyr95, Ile172, Ala173, Tyr176, *Phe335 (O…O)*, Gly338, Phe341, Ser438, Gly442, Leu443
ZINC4038093	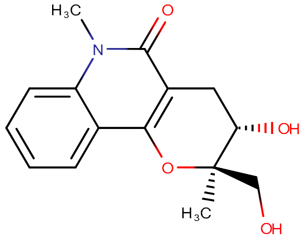	Trp86, Gly121, Ser125, Tyr337, Tyr341	Tyr71, Thr72, Phe108	Ile62, Ala83, Leu132, Asp133, Tyr134, Val135, Thr138, Leu188, Cys199	Gly100, Arg104, *Phe335 (O…HO)*, Gly498
ZINC4040762	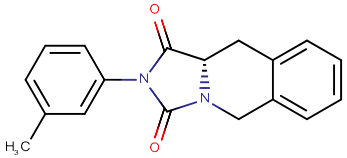	Asp74, Trp86, Gly120, Gly121, Tyr124, Ser125, Trp286, Tyr337, Tyr341	Tyr71, Phe108, Lys224	Ile62, Gly65, Gly68, Val70, *Lys85 (NH_3^+…O)*, Cys199	Tyr95, Tyr176, Phe335, Thr497, Gly498
ZINC4041614	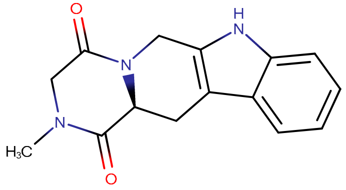	Tyr72, Asp74, Trp86, Gly121, Tyr124, Ser125, *Tyr337 (HO…O)*, Tyr341	Tyr71, Thr72, Phe108, Tyr198	Ala83, *Lys85 (NH_3^+…O)*, Leu132, Val135, Leu188	Tyr95, Ala169, Ile172, Ala173, Tyr176, Gly338, Ser438, Ser439, Gly442

ZINC code	Structure	AChE	BACE1	GSK3β	SERT
ZINC4082030	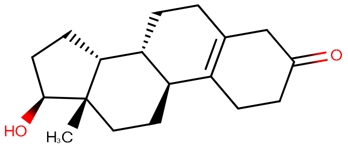	Asp74, Trp86, Gly121, Tyr124, Ser125, Glu202, Ser203, Tyr337, Phe338, His447, Gly448	Tyr71, Gln73, Lys107, Phe108,	Ile62, Val70, Ala83, Lys85, Leu132, *Val135 (NH…O)*, Asn186, Leu188	Asp98, Gly100, Phe335, Glu493, Thr497, Gly498
ZINC4090104	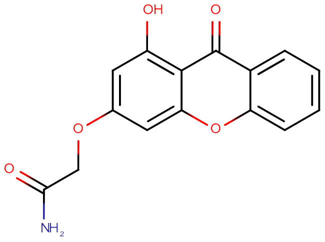	Asp74, Trp86, Gly121, Ser125, *Tyr337 (OH…O)*, Tyr341	*Ser35 (OH…O)*, Asn37, *Tyr71 (OH…O)*, Trp76, Ile118	Ile62, Val70, *Lys85 (NH_3^+…O)*, Val135, Leu188, Cys199, Asp200	Ile172, Asn177, Phe335, Gly338, Phe341, Leu443, Val501
ZINC4538008	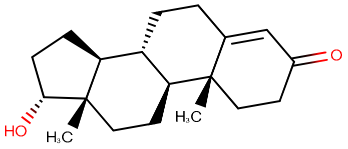	Tyr72, Asp74, Tyr124, Trp286, Leu289	Tyr71, Gln73, Phe108, Ile118	Ile62, Val135, *Thr138 (OH…OH)*, Arg141, Gln185, Leu188	Ala96, Asp98, Ile172, Tyr176, Leu337, Gly338, Phe341, Ser438, Ser439
ZINC4654724	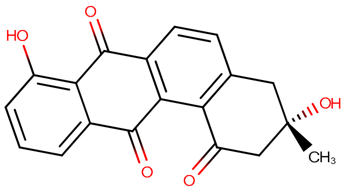	Tyr72, Asp74, Thr75, Tyr124, Trp286, Ser293, Tyr341	Tyr71, Gln73, Phe108, Ile226, Asp228, Thr231, Val332	Ile62, Asn64, Val70, Ala83, Lys85, Leu132, Tyr134, Val135, Asp200	*Tyr175 (OH…O)*, Tyr176, Phe335, Phe341, Ser438, Gly442, Glu493, *Thr497 (O…HO)*

ZINC code	Structure	AChE	BACE1	GSK3β	SERT
ZINC4655109	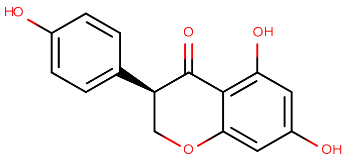	Asp74, Trp86, Tyr124, Ser125, *Glu202 (COO$^-$…HO)*, Tyr337, Tyr341	Gly34, Tyr71, *Lys107 (O…HO)*, Phe108	Val70, Ala83, Lys85, Leu132, *Val135 (NH…OH)*, Leu188, Asp200	Tyr95, Ala169, Ile172, Ala173, Tyr176, Asn177, Phe335, Gly338, Ser438, Ser439, Gly442
ZINC5179869	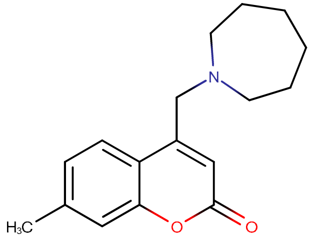	Asp74, Trp86, Tyr124, *Ser125 (HO…O)*, Trp286, Tyr337, Tyr341	Ser35, Tyr71, Gln73, Phe108, Tyr198	Val70, Ala83, Lys85, Val135, Gln185, Leu188, Asp200	Ile172, Phe341, Gly442, Val501
ZINC5733557	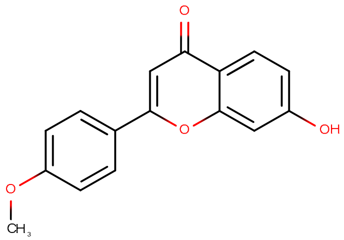	Asp74, Trp86, Tyr124, Ser125, Trp286, *Phe295 (NH…O)*, Phe338, Tyr341	Ser35, Tyr71, Tyr198	Ile62, Leu132, *Val135 (NH…O)*, *Arg141 (NH2…OH)*	Ala169, Ile172, Tyr176, Phe341, Ser439, Gly442, Val501
ZINC6525252	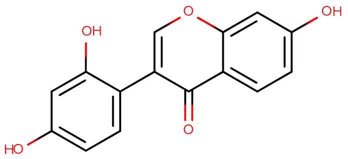	Tyr72, Val73, Asp74, Thr83, Trp86, Tyr124, Trp286, Tyr341	Tyr71, Phe108	Val70, *Val135 (NH…O)*, Leu188	Tyr95, Ile172, Tyr176, Ser336, Ser438, Ser439, Gly442

ZINC code	Structure	AChE	BACE1	GSK3β	SERT
ZINC8623851	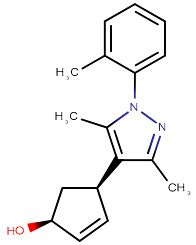	Tyr72, Val73, Asp74, Thr75, Tyr124, Trp286, Tyr341	Tyr71	Ile62, Val70, Lys85, Leu132, Val135, *Pro136 (O…HO)*, Leu188, Cys199, Asp200	Ile172, Tyr175, Tyr176, Phe335, Gly338, *Thr497 (OH…OH)*
ZINC14684865	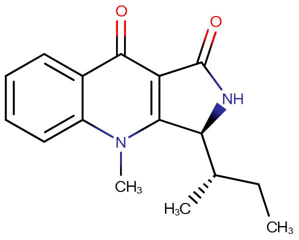	Tyr72, Asp74, Trp86, Asn87, Gly121, Tyr124, Ser125, Tyr337, Tyr347	Tyr71, Thr72, *Gln73 (NH…O)*, Ile118	Val70, Lys85, Leu132, *Val135 (NH…O)*, Thr138, Leu188	Ile172, Tyr176, Phe335, Gly338, Phe341, Ser438
ZINC14719962	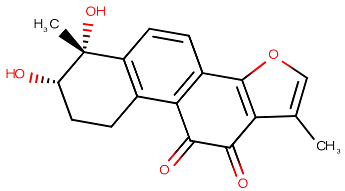	Asp74, Trp286, Ser293, Tyr341	Tyr71, Phe108, Tyr198	*Ile62 (O…HO)*, Lys85, Leu132, Thr138, Arg141	Tyr95, Asp98, Ala169, Ile172, Ala173, Tyr176, Ser336, Gly338, Phe341, Ser438, Ser439
ZINC14829817	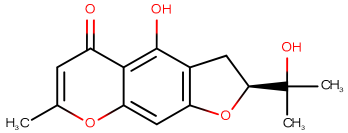	Tyr72, Asp74, Trp86, Gly121, Tyr124, Ser125, Tyr337, Tyr341	Tyr71, Phe108, Ile226, Asp228	Ile62, Leu132, Tyr134, *Val135 (NH…OH)*, Thr138, Arg141	*Tyr95 (HO…HO)*, Ile172, Tyr176, Phe335, Ser439, Gly442

ZINC code	Structure	AChE	BACE1	GSK3β	SERT
ZINC31158814	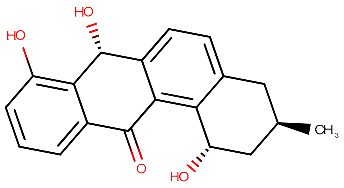	Asp74, Tyr124, Trp286, Ser293, Val294, Tyr341	*Gly34 (O…HO),* Tyr71, Gln73, Phe108	Val70, Lys85, Leu132, Val135, Pro136, Thr138, Arg141	Tyr95, Ile172, Alal173, Tyr176, Phe335, Phe341, *Ser438 (O…HO),* Ser439, Gly442
ZINC35446833	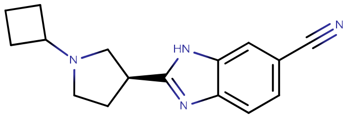	Asp74, Trp86, Tyr124, Trp286, Tyr337, Tyr341	Gly34, Tyr71, Phe108, Lys224, Thr329	Ile62, Val70, Ala83, Lys85, Val135, Lys183, Leu188, Cys199	Asp98, Ala169, Ile172, Ala173, Asn177, Ser336, Phe341, Ser439, Gly442, Leu443
ZINC35446839	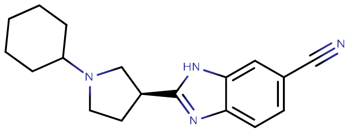	Asp74, Trp86, Tyr124, Trp286, Ser293, Tyr337, Tyr341	Tyr71, Phe108, Trp115, Lys224, Arg235, Thr329	Ile62, Gly65, Val70, Ala83, Lys85, Tyr134, Val135, Leu188, Cys199	Ala169, Ile172, Ala173, Asn177, Phe334, Phe335, Gly338, Phe341, Ser439, Gly442, Leu443, Val501
ZINC35466008	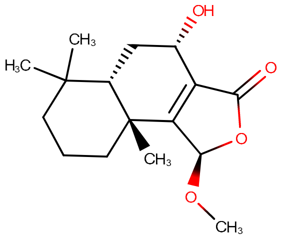	Tyr72, Leu76, Tyr124, Trp286, Phe295, Phe297, Phe338, Tyr341	Ser35, Tyr71, *Gln73 (NH…OH)*	Ile62, Val70, *Lys85 (NH_3^+…O),* Thr138, Leu188, Cys199, Asp200	Tyr95, Ile172, *Phe335 (O…HO),* Ser336, Gly338, Phe341, Val501

ZINC code	Structure	AChE	BACE1	GSK3β	SERT
ZINC38304118	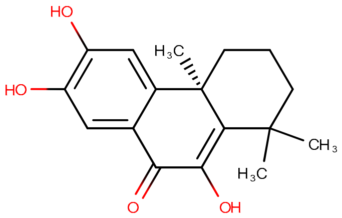	Tyr124, Trp286, Tyr341	Tyr71, Thr72, *Gln73 (NH…O, O…HO),* Phe108	Gly63, Asn64, Val70, Val110, Leu132, *Val135 (NH…O),* Leu188	Asp98, Ile172, Tyr176, Phe335, Gly338, Phe341, Ser438, Ser439, Gly442
ZINC49169727	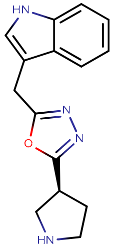	Tyr72, Asp74, Trp86, Tyr124, Ser203, Tyr337, Tyr347	Tyr71, *Thr72 (NH…N),* Phe108, Lys224, Thr329	Ile62, Ala83, Lys85, Val135, Leu188	Tyr95, Ala96, Asp98, Ala169, Ile172, Ala173, Leu337, Gly338, Ser439, Phe341, Gly442
ZINC49170581	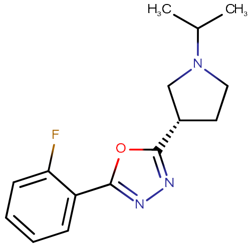	Tyr72, Asp74, Trp86, Gly121, Tyr124, Trp286, Tyr337, Tyr341	Gly34, Tyr71, Phe108, Val332, Thr329	Ile62, Ala83, Lys85, Val135, Asn186, Leu188, *Asp200 (COO-…HN^{+})*	Tyr95, Ala169, Ile172, Ala173, Phe335, Gly338, Phe341, Ser439, Gly442, Leu443, Val501
ZINC49170716	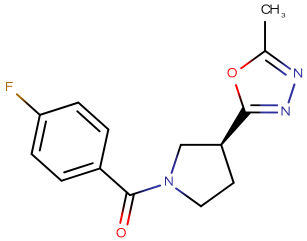	Asp74, *Tyr124 (OH…O),* Trp286, *Tyr337 (OH…N, HO…HN), Tyr341 (HO…HN)*	Gly13, Tyr71, Ile110	Ile62, Val70, Lys85, Leu188, Asp200	Tyr95, Ile172, Tyr176, Phe335, Phe341, *Thr497 (OH…N)*

ZINC code	Structure	AChE	BACE1	GSK3β	SERT
ZINC49170953	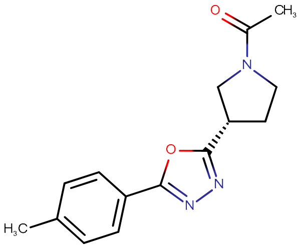	Tyr72, Asp74, Trp86, Gly121, *Tyr124 (HO…O)*, Trp286, Tyr337, Tyr341	Tyr71, Phe108, Arg235, Thr239	Ala83, *Lys85 (NH_3^+…N)*, Val135, Leu188	Tyr95, Ile172, Tyr175, Phe335, *Thr497 (HO…HN, OH…N)*, Gly498, Val501
ZINC100074489	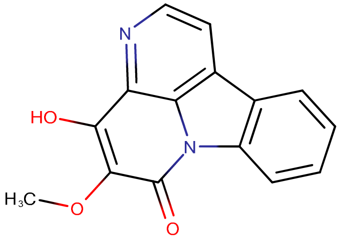	Trp86, Glu120, Gly121, Ser125, *Tyr337 (OH…O)*	Tyr71, Thr72, Gln73, Phe108, Ile118, Tyr198	Lys85, Cys199	Arg104, Gln332, Phe335, Thr497, Gly498
ZINC225417079	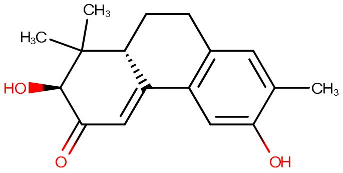	Tyr72, Asp74, Trp86, Tyr124, Trp286, Tyr337, Tyr341	Ser35, Tyr71, Gln73, Phe108, Ile118	Ile62, Ala83, Leu132, Asp133, Tyr134, *Val135 (NH…OH)*, Thr138, Arg141, Leu188	Ile172, Ala173, Tyr176, Asn177, Phe341, Ser439, Gly442, Leu443
ZINC225482257	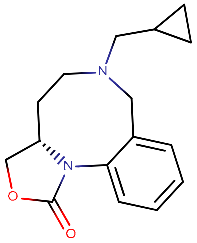	Tyr72, Asp74, *Thr75 (NH…O)*, Leu76, Trp286, Val294, Phe295, Phe338, Tyr341	Tyr71, Gln73, Phe108, Ile118	Ile62, Val70, Ala83, Leu132, Val135, Leu188, Cys199	*Tyr95 (O... O)*, Asp98, Ile172, Phe341, Ser439, Gly442

ZINC code	Structure	AChE	BACE1	GSK3β	SERT
ZINC252463663	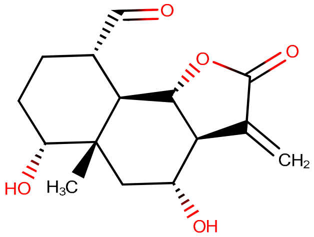	Asp74, Trp86, Gly120, Gly121, Tyr124, Ser125, Tyr133, Tyr337, Tyr341	Ser35, Tyr71, *Thr72 (NH…O)*, Ile118, Asp228	Ile62, Gly63, Val70, Lys85, Cys199, Asp200	Asp98, Ile172, Tyr176, Phe341, Ser438, Ser439

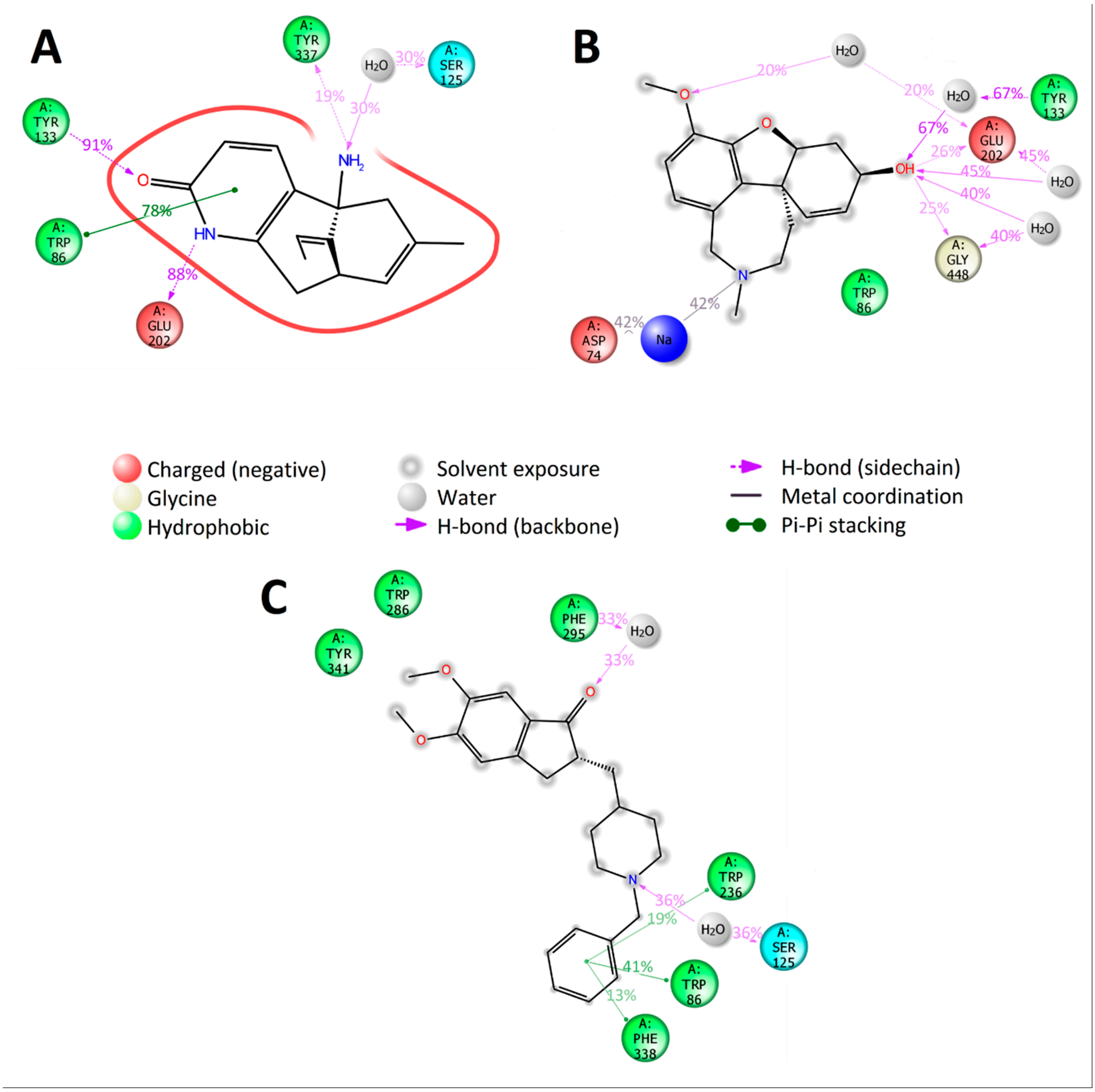

Figure S1. 2D summary diagram of molecular dynamics calculated contacts between AChE and inhibitors **(A)** (-) – huperzine A, **(B)** (-)-galantamine and **(C)** donepezil. Interactions that occur more than 10% of the simulation time are shown.

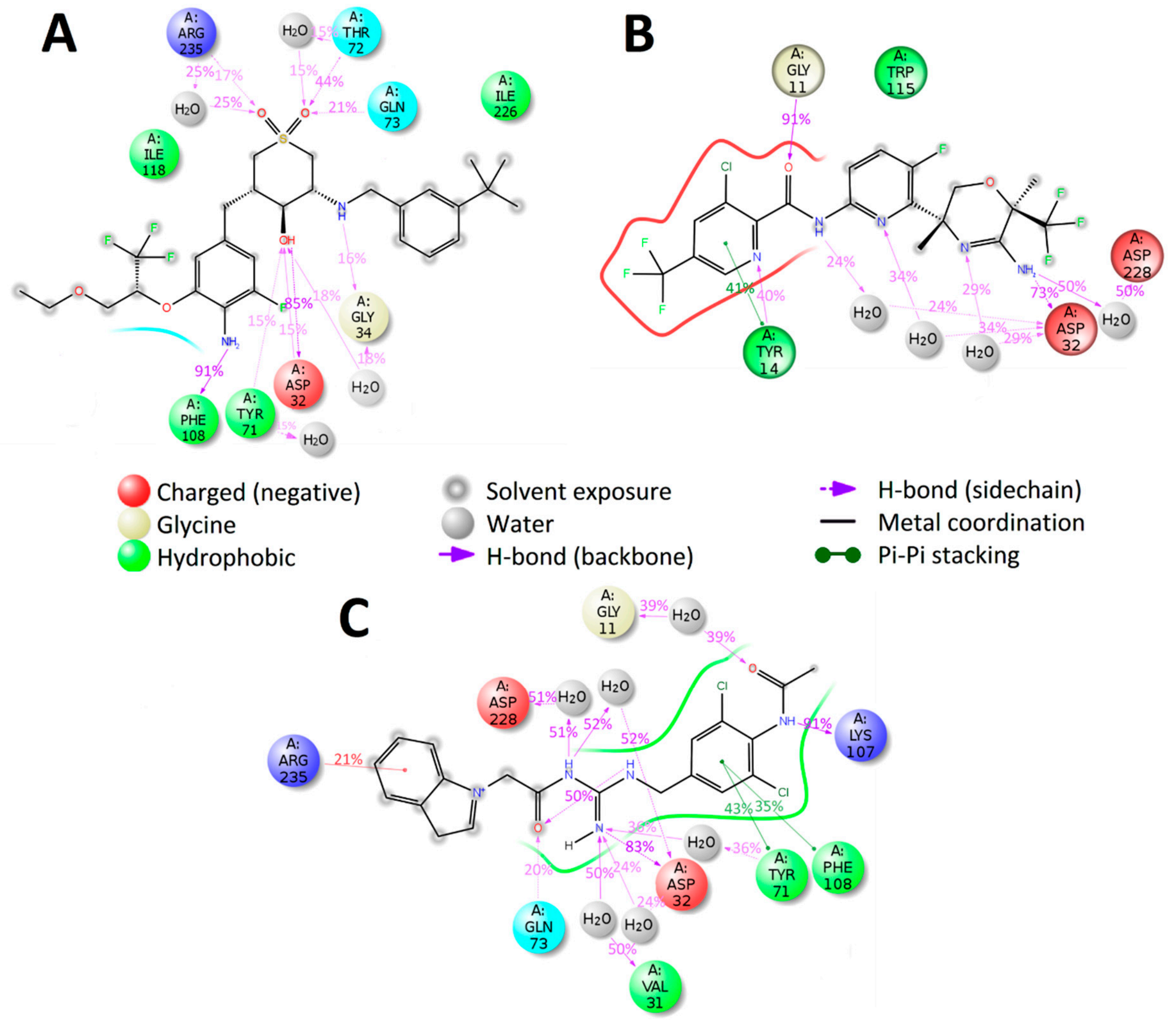

Figure S2. 2D summary diagram of molecular dynamics calculated contacts between BACE1 and inhibitors (A) NVP-BXD552, (B) CNP520 and (C) VTI. Interactions that occur more than 10% of the simulation time are shown.

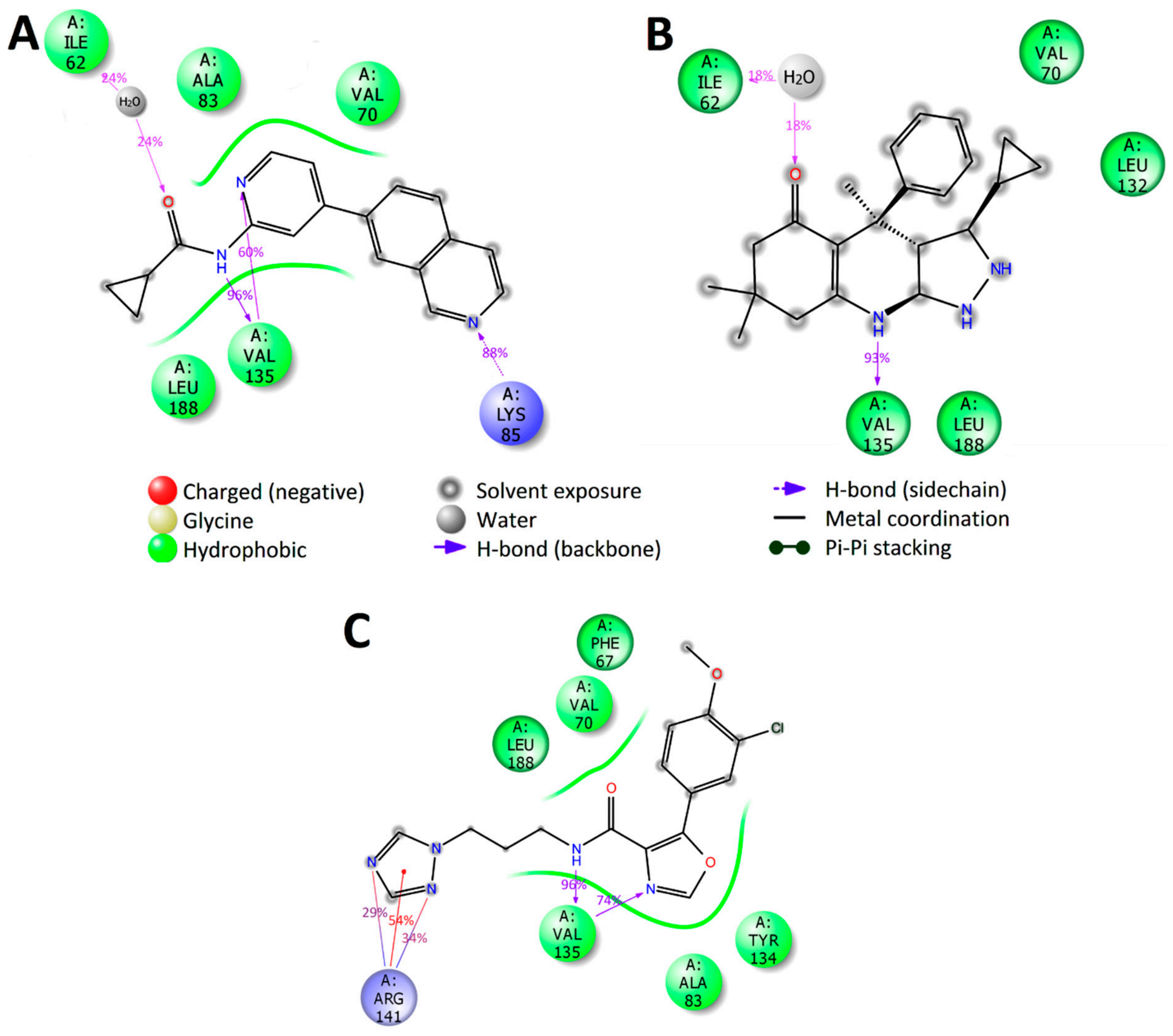

Figure S3. 2D summary diagram of molecular dynamics calculated contacts between GSK3β and inhibitors (A) N-[4-(isoquinolin-7-yl)pyridin-2-yl]cyclopropanecarboxamide, (B) BRD0209 and (C) PF-04802367. Interactions that occur more than 10% of the simulation time are shown.

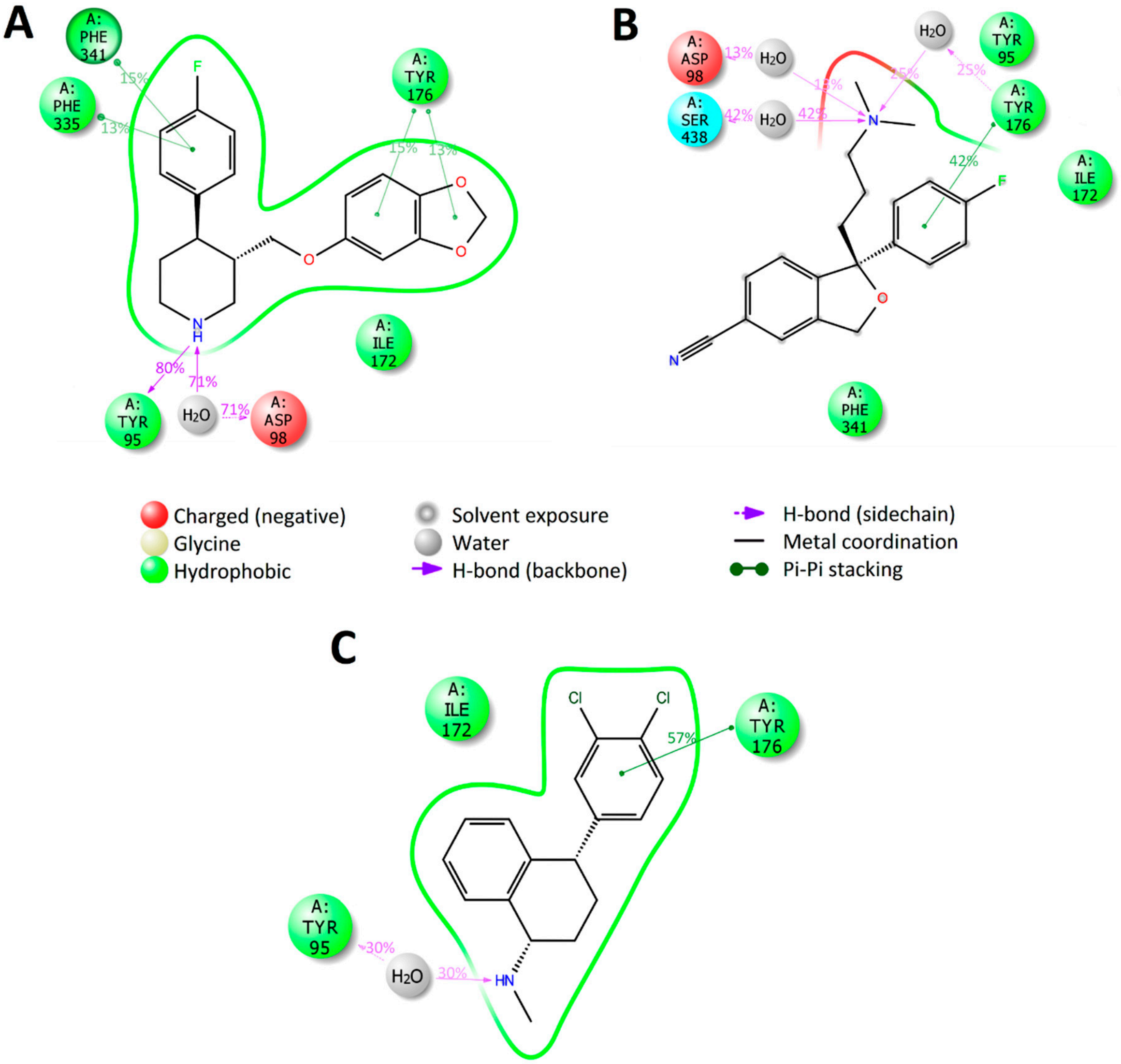

Figure S4. 2D summary diagram of molecular dynamics calculated contacts between SERT and inhibitors (A) paroxetine, (B) s-citalopram and (C) sertraline. Interactions that occur more than 10% of the simulation time are shown.

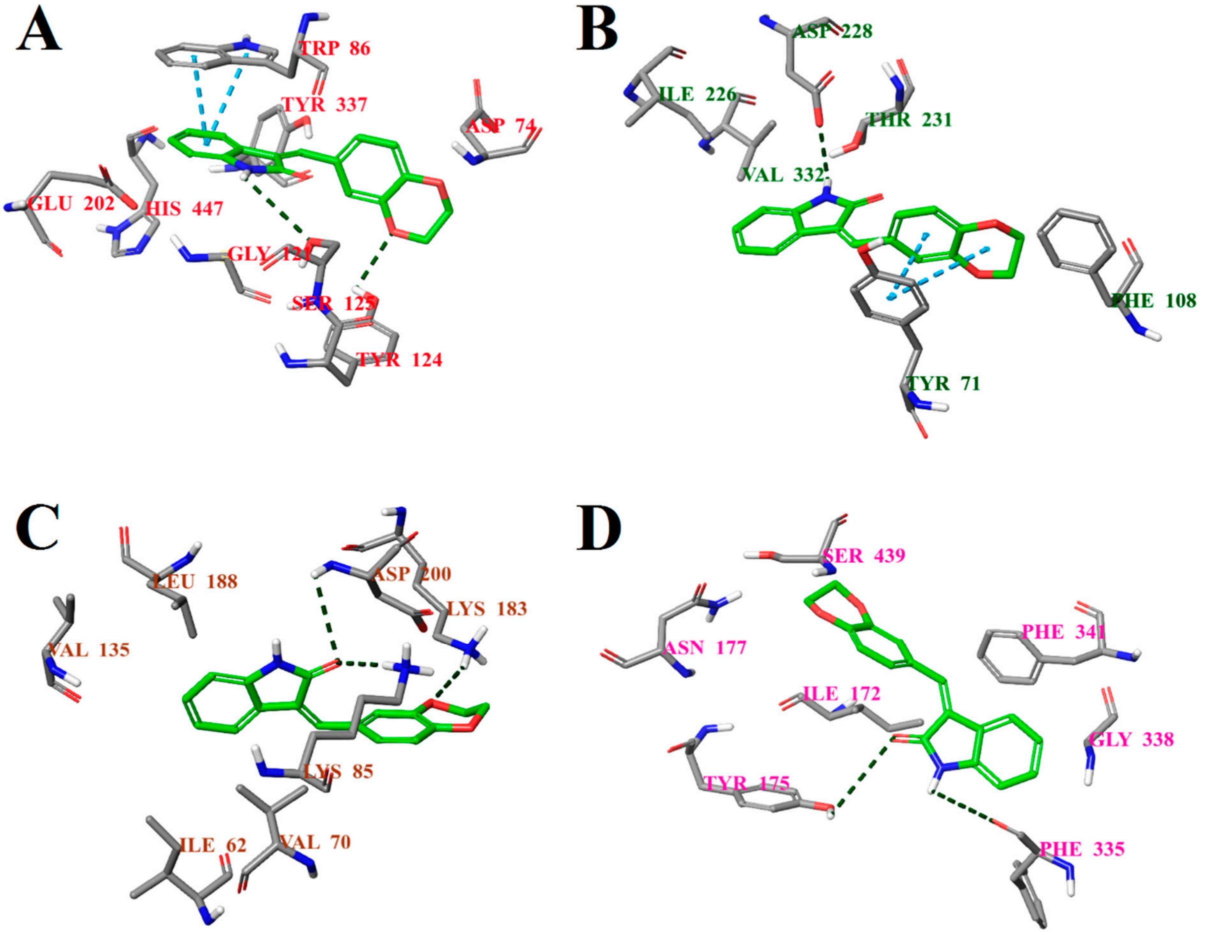

Figure S5. Calculated binding modes of compound ZINC1034491 **(A)** – in the active site of AChE (ID: 4EY6); **(B)** – in the active site of BACE1 (ID: 6EQM); **(C)** – in the active site of GSK3β (ID: 1PYX); **(D)** – in the central active site of SERT (ID: 5I6X). The amino acid residues of target proteins are colored as gray (carbon), blue (nitrogen), red (oxygen), and white (hydrogen). Hydrogen bonds formed between compound and residues of target proteins are represented by green dashed lines, pi-pi stacking is represented by blue dashed line.

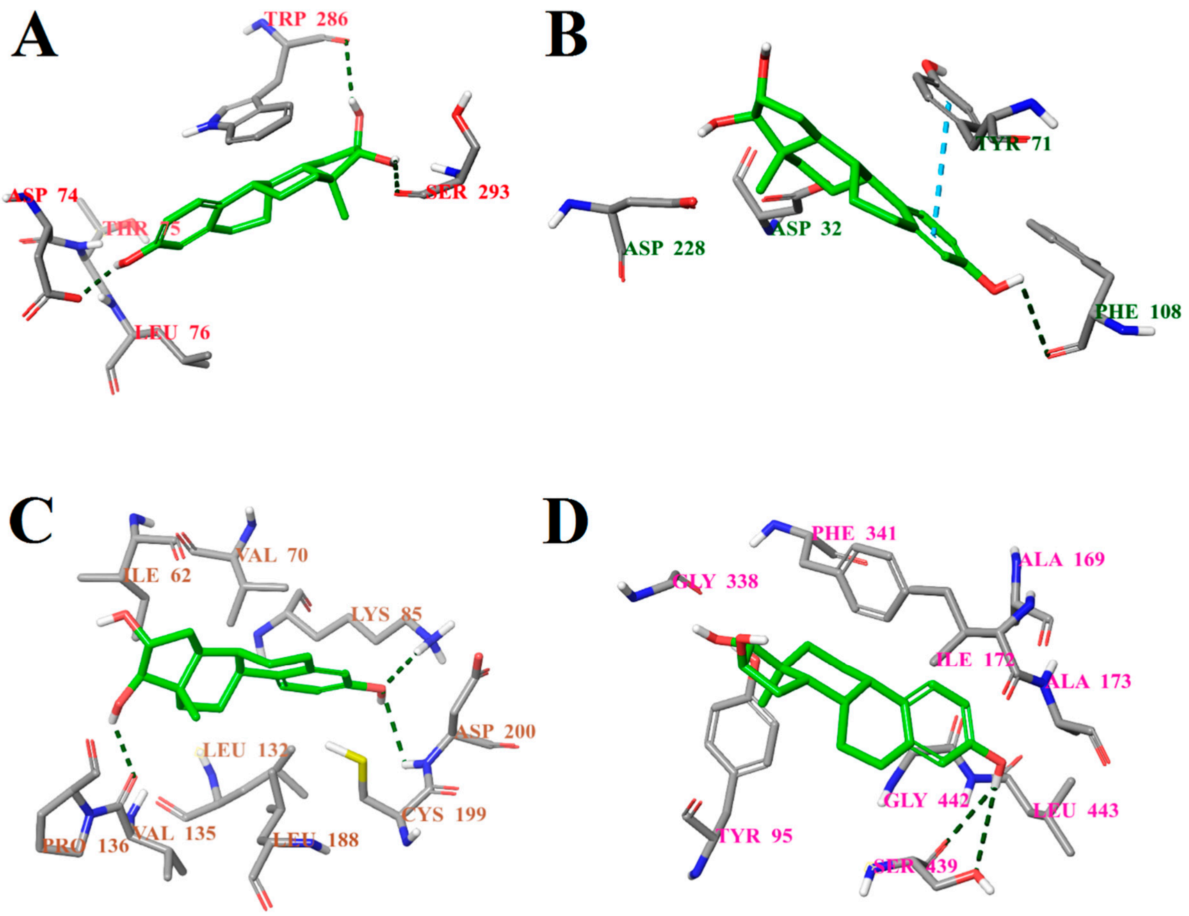

Figure S6. Calculated binding modes of compound ZINC3977996 **(A)** – in the active site of AChE (ID: 4EY6); **(B)** – in the active site of BACE1 (ID: 6EQM); **(C)** – in the active site of GSK3β (ID: 1PYX); **(D)** – in the central active site of SERT (ID: 5I6X). The amino acid residues of target proteins are colored as gray (carbon), blue (nitrogen), red (oxygen), and white (hydrogen). Hydrogen bonds formed between compound and residues of target proteins are represented by green dashed lines, pi-pi stacking is represented by blue dashed line.

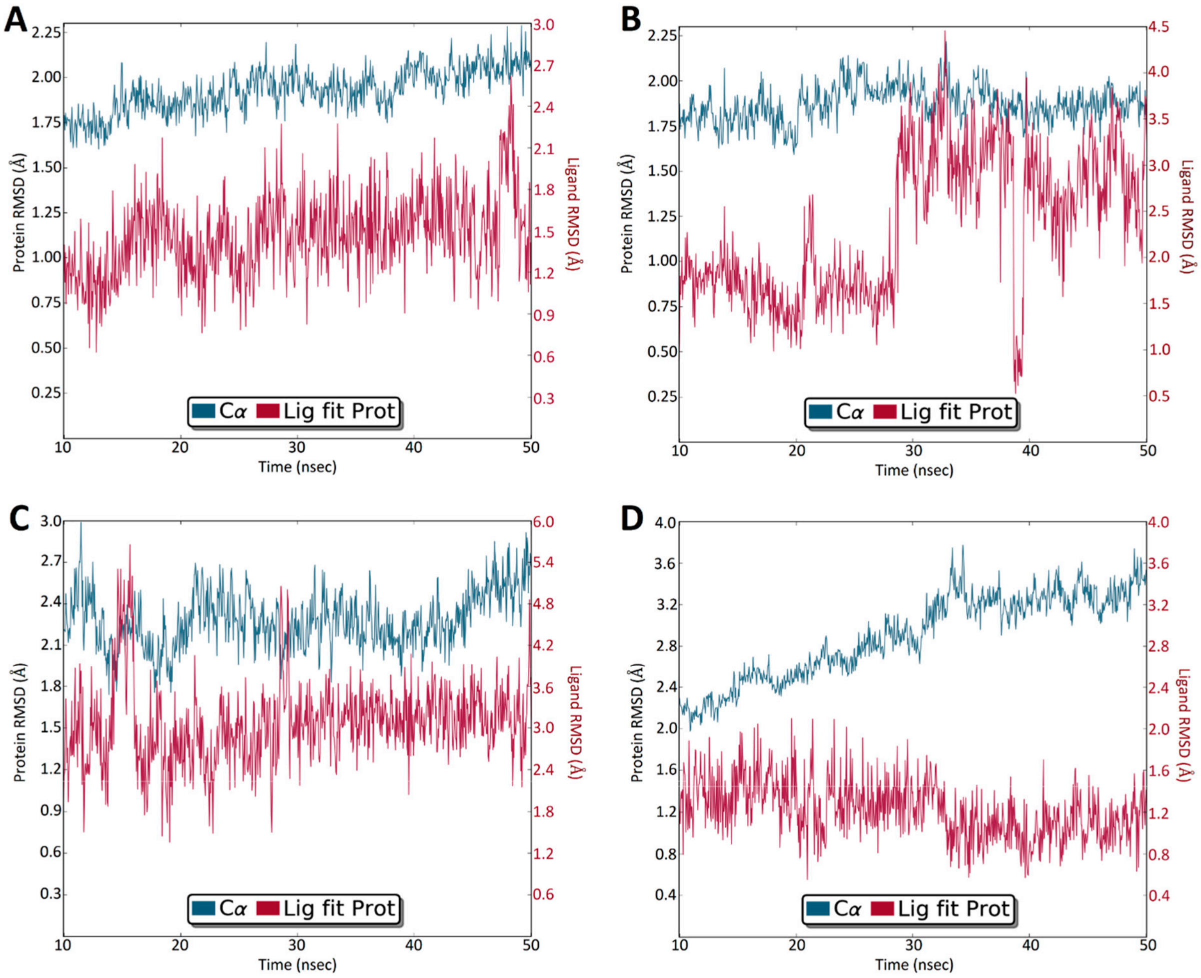

Figure S7. RMSD of the atomic positions for the compounds ZINC4027357 (in red) and the target proteins (in blue): **(A)** – in the active site of AChE (ID: 4EY6); **(B)** – in the active site of BACE1 (ID: 6EQM); **(C)** – in the active site of GSK3β (ID: 1PYX); **(D)** – in the central active site of SERT (ID: 5I6X) of the 50 ns molecular dynamics simulations.

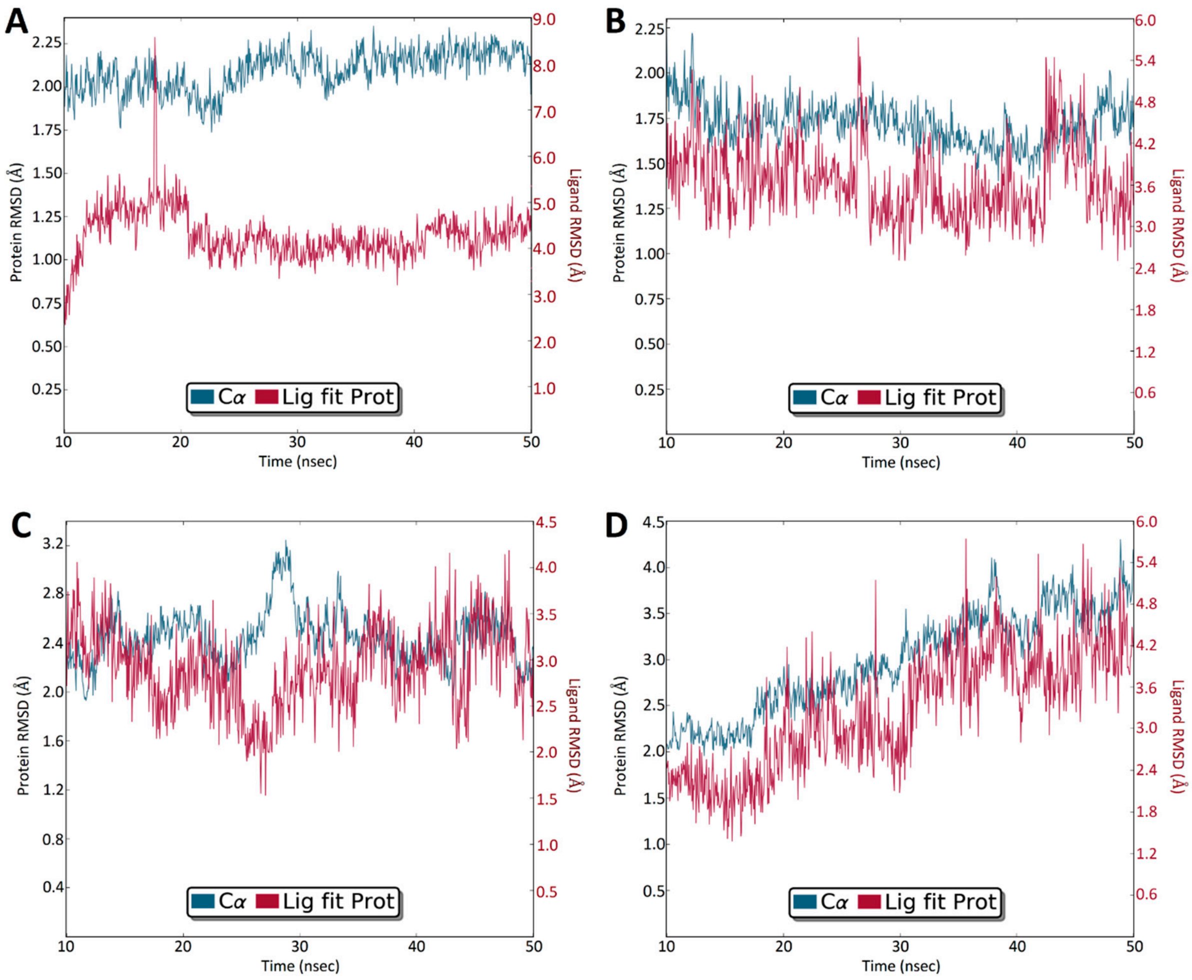

Figure S8. RMSD of the atomic positions for the compounds ZINC1034491 (in red) and the target proteins (in blue): **(A)** – in the active site of AChE (ID: 4EY6); **(B)** – in the active site of BACE1 (ID: 6EQM); **(C)** – in the active site of GSK3β (ID: 1PYX); **(D)** – in the central active site of SERT (ID: 5I6X) of the 50 ns molecular dynamics simulations.

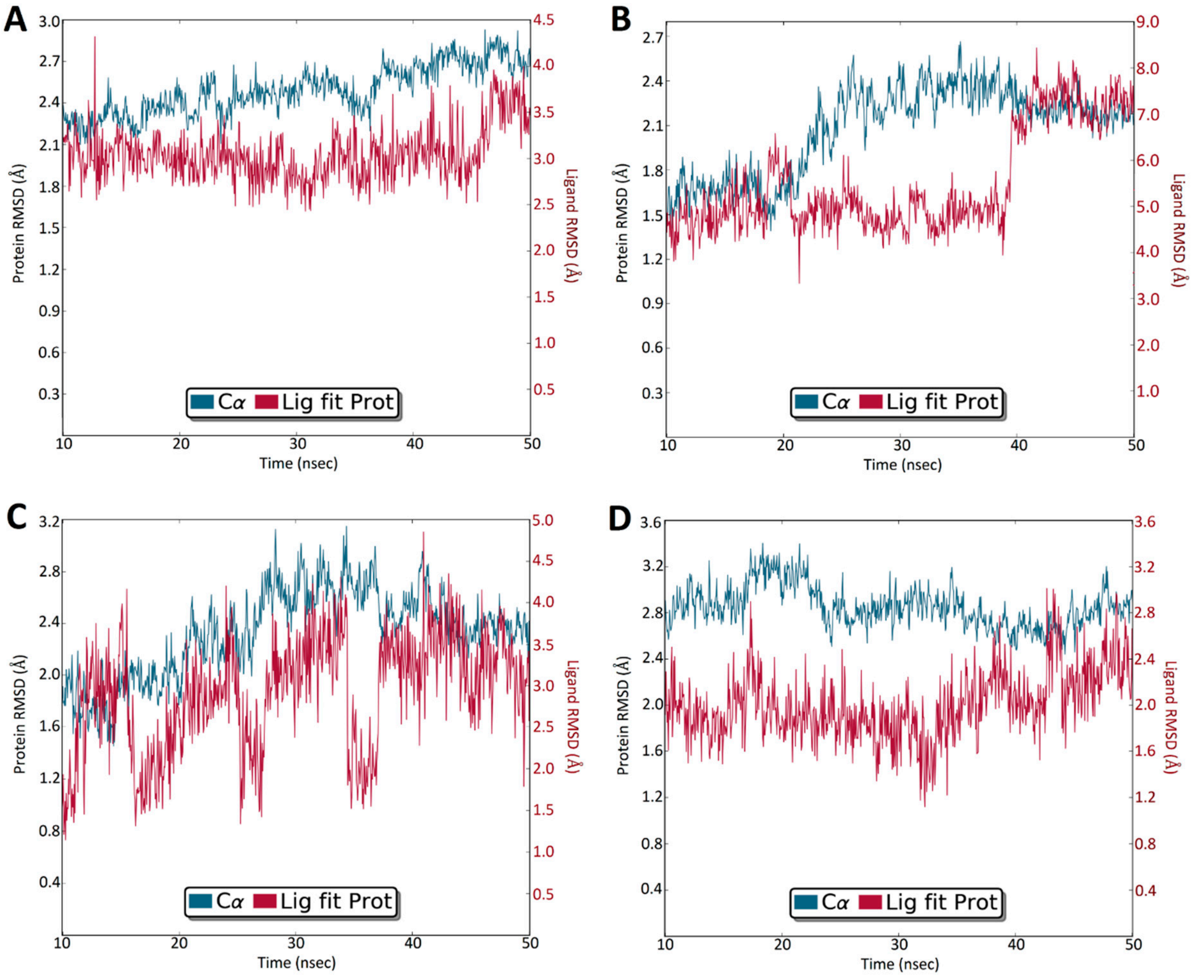

Figure S9. RMSD of the atomic positions for the compounds ZINC3977996 (in red) and the target proteins (in blue): **(A)** – in the active site of AChE (ID: 4EY6); **(B)** – in the active site of BACE1 (ID: 6EQM); **(C)** – in the active site of GSK3β (ID: 1PYX); **(D)** – in the central active site of SERT (ID: 5I6X) of the 50 ns molecular dynamics simulations.

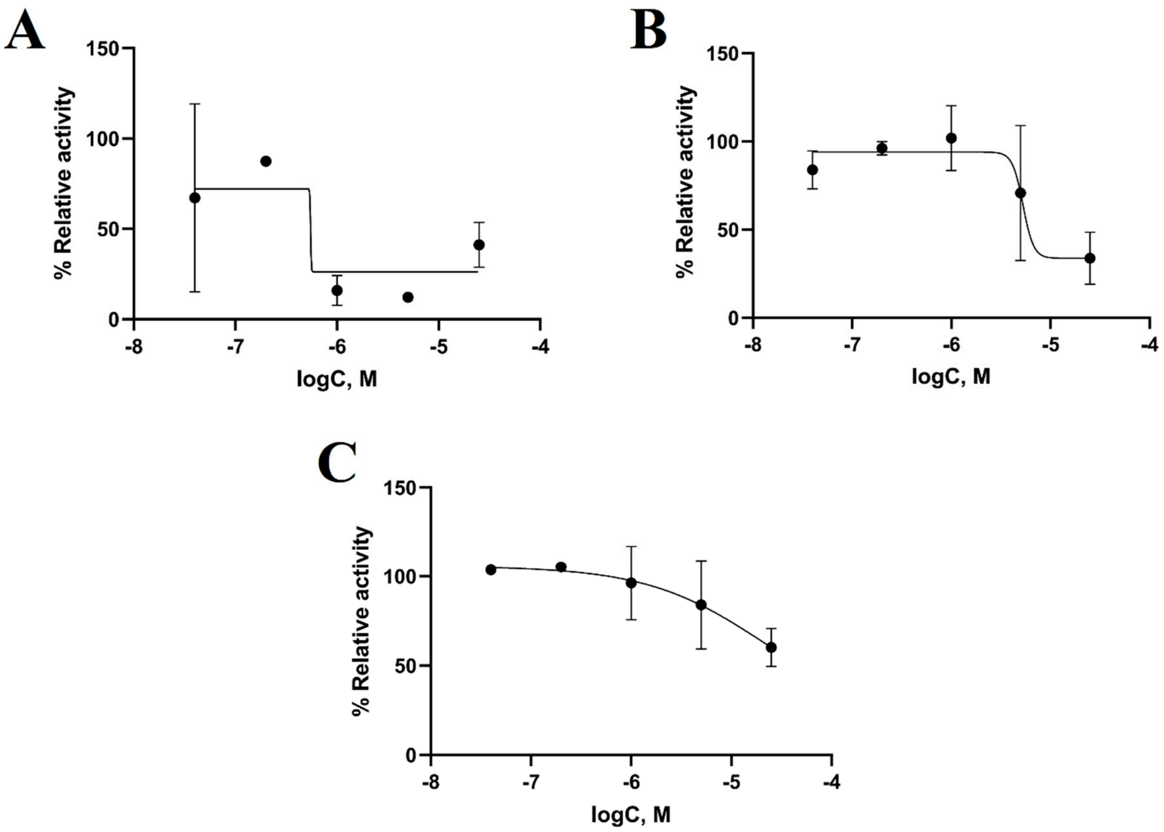

Figure S10. Inhibition of (A) AChE and (B) BACE1 by ZINC4027357 (IC_{50} = 0.55 µM and 5.2 µM, respectively); (C) – Inhibition of AChE by ZINC1801081 (IC_{50} = 20.9 µM).
